# Unravelling the Glycan Code: Molecular Dynamics and Quantum Chemistry Reveal How O‐Glycan Functional Groups Govern OgpA Selectivity in Mucin Degradation by *Akkermansia muciniphila*

**DOI:** 10.1111/1751-7915.70091

**Published:** 2025-04-03

**Authors:** Mohammad Khavani, Aliyeh Mehranfar, Mohammad R. K. Mofrad

**Affiliations:** ^1^ Molecular Cell Biomechanics Laboratory, Department of Bioengineering and Mechanical Engineering University of California Berkeley Berkeley California USA; ^2^ Molecular Biophysics and Integrative Bioimaging Division Lawrence Berkeley National Lab Berkeley, CA USA

**Keywords:** *Akkermansia muciniphila*, enzyme mechanism, gut microbiome, mucin, *O*‐glycan, simulation

## Abstract

Mucins, heavily *O*‐glycosylated glycoproteins, are a key component of mucus, and certain gut microbiota, including *Akkermansia muciniphila* , can utilise mucin glycans as a carbon source. *Akkermansia muciniphila* produces the *O*‐glycopeptidase enzyme OgpA, which cleaves peptide bonds at the N‐terminus of serine (Ser) or threonine (Thr) residues carrying *O*‐glycan substitutions, with selectivity influenced by the *O*‐glycan functional groups. Using molecular dynamics (MD) simulations and quantum chemistry calculations, we explored how different *O*‐glycan groups affect OgpA's selectivity. Our results show that peptides bind to the enzyme via hydrogen bonds, π–π interactions, van der Waals forces and electrostatic interactions, with key residues, including Tyr90, Val138, Gly176, Tyr210 and Glu91, playing important roles. The primary determinant of selectivity is the interaction between the peptide's functional group and the enzyme's binding cavity, while peptide–enzyme interface interactions are secondary. Quantum chemistry calculations reveal that OgpA prefers peptides with a lower electrophilic character. This study provides new insights into mucin degradation by gut microbiota enzymes, advancing our understanding of this critical biological process.

## Graphical abstract

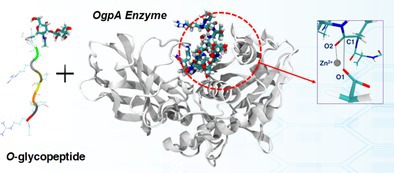

## 1 Introduction

The intricate and diverse community of microorganisms in the human gut, known as the gut microbiota, plays a critical role in maintaining human health (Hansson 2020). This microbiota engages in interactions with the protective mucus layer that coats the intestinal epithelium (Johansson et al. 2008). Interestingly, when we compare the small intestine to the colon, we observe a distinction in the organisation of the mucus layer. In the colon, there are two distinct layers: the inner layer, which adheres closely to the epithelium, is densely packed and typically lacks a significant bacterial presence, while the outer layer of mucus is more loosely structured and hosts a thriving population of microbiota (Martens, Chiang, and Gordon 2008). The dual‐layered arrangement of mucus in the colon plays a vital role in maintaining the host's health. The outer mucus layer provides an ideal environment for gut bacteria to thrive, while the inner layer behaves as a barrier against the bacteria to protect the intestinal epithelium from inflammation and close contact (Sommer et al. 2014; Johansson et al. 2014). Previous studies have investigated the critical role of mucus in maintaining intestinal homeostasis and the complex interplay between mucus and gut microbiota (Calvigioni et al. 2024). The structural and functional characteristics of mucins, the glycoproteins that form the mucus layer, along with their dynamic glycosylation profiles, directly influence the composition and localisation of gut microbial communities. Moreover, mucus serves as both a protective barrier and a nutrient source for commensal bacteria, mediating microbial interactions and preventing pathogen colonisation. Furthermore, alterations in mucus properties, such as changes in glycosylation or thickness, are associated with dysbiosis and various diseases, including inflammatory bowel disease and colorectal cancer.

Mucins form the most composition of the mucus, which are constructed of heavily *O*‐glycosylated glycoproteins. These host glycans play a crucial role in determining which bacteria can successfully colonise the host (Desai et al. 2016; Schroeder et al. 2018). The gut microorganisms can utilise the mucin as a source of nutrients, in which the degradation of mucin can lead to changes in the composition of the microbiota and a thinner, more permeable mucus barrier (Everard et al. 2013; Png et al. 2010; Layunta et al. 2021; Tailford et al. 2015; Robbe et al. 2004). Many reports show that mucus thickness and protein composition perturbation are associated with various metabolic diseases, cardiovascular diseases, cancers and inflammatory bowel diseases, and type‐II diabetes (Ley et al. 2006; Ridaura et al. 2013; Sonnenburg and Bäckhed 2016; Jonsson and Bäckhed 2017).

The mucin family has 20 members, which are divided into non‐gel‐forming secreted, gel‐forming secreted and membrane‐bound (Kudelka et al. 2020; Hansson 2020). Mucin 2 (MUC2) is a member of the mucin (MUC) family that forms the gut mucus by forming a polymeric network of the glycoprotein (Wang, Wu, and Ribbeck 2021). Moreover, the MUC2, MUC5AC, MUC6 and MUC5B belong to the gel‐forming secreted group with a considerable contribution to the gut mucus composition (Grondin et al. 2020). Compared to the other types of mucins, the MUC2 in the colons of mice and humans is the major gel‐forming mucin in the mucus system (Linden et al. 2008). This MUC2 structure is exclusively synthesised by the goblet cells found in the colonic epithelial of the intestines and colon. Within these cells, the proline, serine and threonine‐rich (PTS) domains of the monomers of MUC2 undergo glycosylation. At the C‐terminus, these monomers dimerise, and at the N‐termini, they come together to form trimers (Van Klinken et al. 1999; Johansson, Sjövall, and Hansson 2013; Godl et al. 2002). Then, the trimer dimers covalently bind to each other to form a polymeric network. Once they are released by the goblet cells, they form the gel through a hydration process that acts as a barrier and lubricant to facilitate the passage of luminal contents without damaging tissues and contribute to the overall function of the intestinal system (Birchenough et al. 2015; Bergstrom and Xia 2013). MUC2 is composed of at least five monosaccharides including fucose (Fuc), galactose (Gal), N‐acetylglucosamine (GlcNAc), N‐acetylgalactosamine (GalNAc) and sialic acid (Sia) (Koropatkin, Cameron, and Martens 2012; Ten Hagen, Fritz, and Tabak 2003). The *O*‐linked is the main glycosylation in the MUC2, which occurs due to the functionalisation of hydroxyl groups of serine and threonine residues with GalNAc (An et al. 2007).

Certain members of the microbiota have the ability to use mucin glycan groups as a source of carbon. In order to break down these host glycans, bacteria express a variety of carbohydrate‐active enzymes, including glycoside hydrolases, sulphatases and esterase, each of which acts on specific types of linkages (Raba and Luis 2023). *Akkermansia muciniphila* is a mucin‐degrading bacterium commonly present in the human gut, which produces an *O*‐glycopeptidase enzyme that specifically cleaves the peptide bond located at the N‐terminus of serine (Ser) or threonine (Thr) residues carrying an *O*‐glycan substitution (Collado et al. 2007). *Akkermansia muciniphila* is a Gram‐negative bacterium belonging to the phylum Verrucomicrobia (Derrien et al. 2008). In healthy adults, it typically makes up approximately 5% of the total bacterial population in the large intestine (Canfora and Blaak 2020). Several reports indicated that this bacterium is effective on the gut barrier function and other physiological phenomena during obesity and type‐II diabetes (Dao et al. 2016; Plovier et al. 2017). *Akkermansia muciniphila* through degrading mucin regulates the mucus thickness and gut barrier integrity, which shows beneficial effects on human health (Derrien et al. 2004). The hydroxyl substitution of Ser and Thr with GalNAc is the first step of the *O*‐glycosylation process. The GalNAc group can be functionalised by other sugars such as sialic acid, fucose and galactose (Martens, Neumann, and Desai 2018). Therefore, there are various mucins with different branches of glycans. The *A. muciniphila* can produce specific enzymes to hydrolyse peptide and glycosidic bonds of the mucin (Luis and Martens 2018). The OgpA (*O*‐glycopeptidase) from *A. muciniphila* can hydrolyse the peptide bond N‐terminal to Thr or Ser residues functionalised with an *O*‐glycan (Yang et al. 2018). This enzyme detects the peptide bonds to hydrolyse based on the type of *O*‐glycan functional groups; however, the role of the functional groups in the enzymatic process still remains a challenge. Therefore, in this study, computational methods such as full atomistic molecular dynamic (MD) simulations and quantum chemistry calculations were applied to determine the selectivity of the OgpA enzyme at the molecular level. The results of this study can provide a new insight into the mechanism of mucin degradation by the gut microbiota. In essence, by elucidating the molecular‐level role of different *O*‐glycan functional groups in the selectivity of the OgpA enzyme, the study sheds light on how specific bacterial enzymes recognise and target particular glycan structures within mucins. This understanding is crucial for deciphering the initial steps of mucin degradation by gut bacteria. Moreover, the study identifies specific molecular interactions involved in the binding of functionalised peptides within the OgpA enzyme's binding cavity. These interactions, including hydrogen bonds, π–π interactions, van der Waals (vdW) forces and electrostatic interactions, provide valuable insights into how the enzyme recognises and cleaves glycosylated peptide bonds in mucins. Furthermore, by demonstrating that peptides with certain *O*‐glycans form more stable complexes with OgpA compared to others, the study highlights the importance of *O*‐glycan structural diversity in dictating the efficiency of mucin degradation by gut bacteria. This understanding enhances our knowledge of how variations in mucin glycan composition can influence bacterial colonisation and metabolic activity in the gut. On the other hand, given the critical role of mucins in maintaining gut barrier function and host–microbiota interactions, insights gained from this study have implications for understanding the pathophysiology of various metabolic diseases, inflammatory conditions and gastrointestinal disorders associated with alterations in mucin composition and mucus barrier integrity. Understanding the molecular mechanisms of mucin degradation by gut microbiota enzymes could lead to the development of novel therapeutic strategies targeting the gut microbiome to promote mucosal health and ameliorate gut‐related diseases.

## 2 Experimental Procedures

### 2.1 Molecular Dynamics Simulation

**FIGURE 1:**
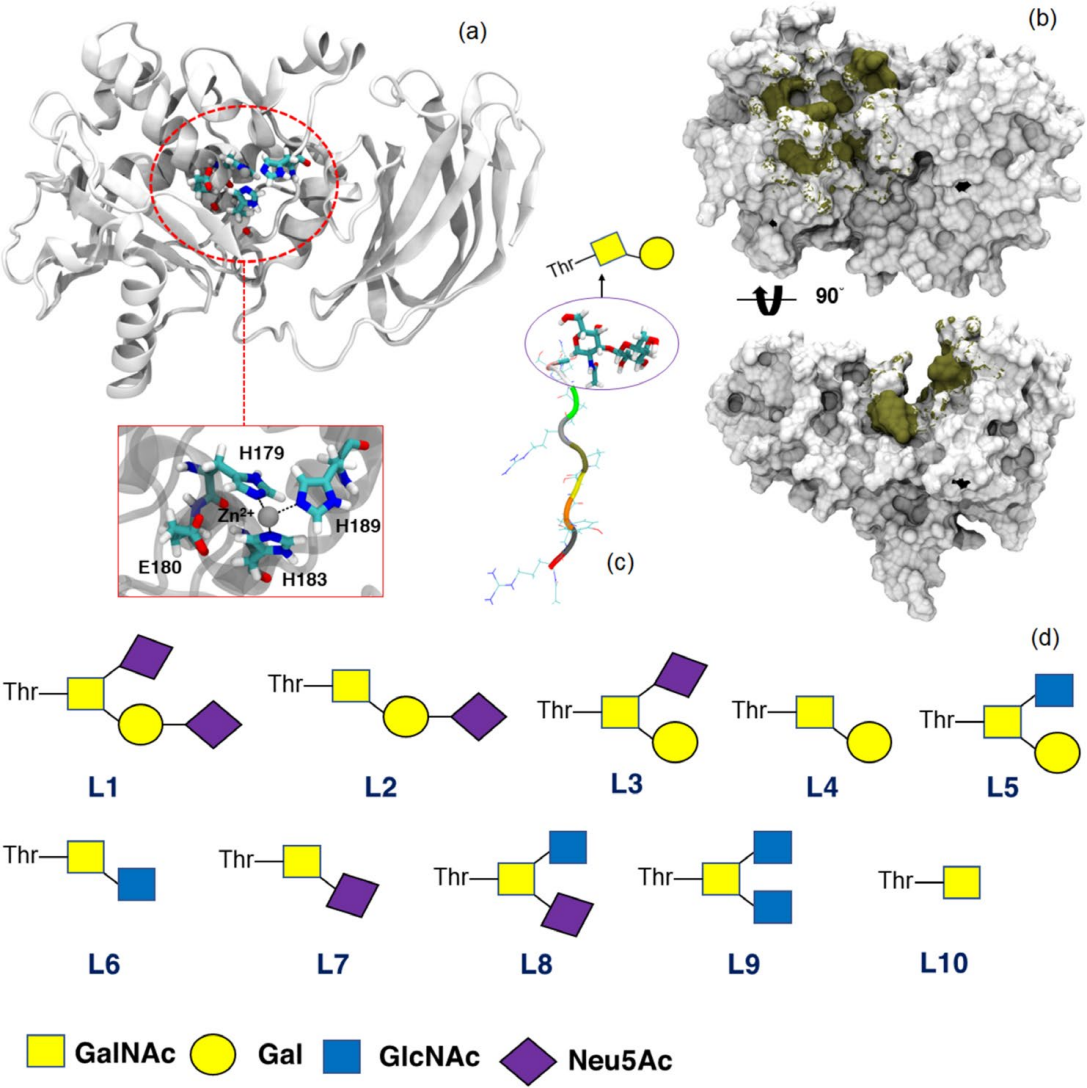
The structure of the OgpA enzyme and its active site (a). The active site of the enzyme is composed of the three His and one Glu residues. The binding site of the enzyme in complexation with the RPYSPRPT(Gal‐GalNAc)SH peptide is highlighted in tan colour in panel (b). The active site and binding site of the enzyme in panels a and b are according to Trastoy et al. 2020. The functionalised RPYSPRPTSH peptide with the Gal‐GalNAc *O*‐glycan is presented in panel (c). A schematic presentation of the selected *O*‐glycan compounds (L) is shown in panel (d) (for the nomenclature of the selected compounds, see Table 1).

The OgpA from *A. muciniphila* as an *O*‐glycopeptidase selectively hydrolyses mucin based on the *O*‐glycan functional groups on N‐terminal Ser and Thr residues. To determine the role of the *O*‐glycan groups on the selectivity and mechanism of the OgpA full atomistic MD simulations were performed. The crystal structure of the OgpA enzyme in complexation with *O*‐glycopeptide (RPYSPRPT(Gal‐GalNAc)SH) is available on the protein data bank with the 6Z2P code. To model the active form of the enzyme, the A179H and A180E mutations were made in the structure of the OgpA (Trastoy et al. 2020). The standard protonation state for all amino acids of the OgpA at pH 7.0 was adopted and checked using the H++ web server (Anandakrishnan, Aguilar, and Onufriev 2012). Three histidine residues including His179, His183 and His189, which are located in the active site of the enzyme, are neutral (see Figure 1a). These residues are coordinated to the Zn^2+^ ion, which the parameters for the Zn^2+^ ion and coordinating His residues were obtained using MCPB.py module in Amber software package, with the structure optimised using B3LYP/6‐31G(d) as part of the procedure (Li and Merz Jr 2016). To analyse the role of the *O*‐glycan groups on the interactions between the peptide and enzyme, the corresponding peptide was substituted with 10 different *O*‐glycan functional groups (Table 1). The schematic structures of the selected *O*‐glycan compounds are presented in Figure 1. To design the structure of the OgpA complexes (using the reported structure by Trastoy et al. (2020)) with different functionalised peptides, AutoDock Vina software was applied (Trott and Olson 2010). The obtained complexes from the docking simulation with the highest binding affinity were employed for the MD simulations.

**TABLE 1:** The selected *O*‐glycan compounds in this study to functionalise the RPYSPRPTSH peptide for complexation with the OgpA enzyme.

*O*‐glycan	Name
L1	Neu5Ac‐α2,3‐Gal‐β1,3‐Neu5Ac‐α2,6‐GalNAc
L2	Neu5Ac‐α2,3‐Gal‐β1,3‐GalNAc
L3	Gal‐β1,3‐Neu5Ac‐α2,6‐GalNAc
L4	Gal‐β1,3‐GalNAc
L5	Gal‐β1,3‐GlcNAc‐β1,6‐GalNAc
L6	GlcNAc‐β1,3‐GalNAc
L7	Neu5Ac‐β1,3‐GalNAc
L8	Neu5Ac‐β1,3‐GlcNAc‐α2,6‐GalNAc
L9	GlcNAc‐β1,3‐GlcNAc‐β1,6‐GalNAc
L10	β1,3‐Gal
L11	Peptide without glycan groups

Abbreviations: Gal, galactose; Glc, glucose; Neu5Ac, N‐Acetylneuraminic acid.

The structure of the OgpA (Figure 1) in the presence and absence of the functionalised peptides was fully solvated with water molecules using the TIP3P solvent model in a cubic box (15 × 15 × 15 nm^3^) within the periodic boundary conditions in all directions (Jorgensen et al. 1983; Khavani, Izadyar, and Housaindokht 2019). To neutralise the charge of the simulated systems, Na^+^ and Cl^−^ counterions were added. The GLYCAM06 force field and FF14SB force field parameters were employed for the *O*‐glycan compounds and the enzyme, respectively (Kirschner et al. 2008; Maier et al. 2015). All the MD simulations were performed using the Amber 22.0 Software package (Case et al. 2020). To start the MD simulations, all the structures were minimised through 100,000 steps of energy minimisation. Then, the temperature of the minimised systems was increased from 0 to 300 K in an NVT ensemble during 5 ns (1 fs time step) by applying a typical force constant of 2.5 kcal.mol^−1^.Å^−2^ for the solute structures. In the next step, 10 ns NPT simulations (1 bar and 300 K, 1 fs time step) were applied for all the systems as the equilibration step without any positional restraint. Finally, 500 ns MD simulations (2 fs time step) were performed using an NPT ensemble on all the equilibrated structures as the product step. To check the effects of the peptide and functional groups on the dynamical properties and structural stability of the OgpA, other two simulations including free OgpA and OgpA complex with the peptide without functional groups were performed.

In the equilibration and product steps, the pressure and temperature were controlled using the Berendsen method (1 ps relaxation time) and Langevin thermostat (1 ps^−1^ collision frequency), respectively (Sindhikara et al. 2009; Uberuaga, Anghel, and Voter 2004). To calculate long‐range electrostatic interactions, the particle mesh Ewald (PME) method with a 12 Å direct cutoff was used (Darden, York, and Pedersen 1993). The SHAKE algorithm was applied to constrain all bonds involving hydrogen atoms during the MD simulations (Ryckaert, Ciccotti, and Berendsen 1977). The binding energies of the complexation process between OgpA and different functionalised peptides have been evaluated using molecular mechanics generalised Born surface area (MM‐GBSA) (Miller III et al. 2012). The final 50 ns of the product step was selected to calculate the binding energies of the complexation process.

### 2.2 DFT‐D3 Calculations

Since the OgpA selects the mucin chains in the degradation process based on the functional groups, *O*‐glycan substitutions should be important in the chemical properties and reactivity features of the peptides (chains) in complexation with the enzyme. Therefore, the density functional theory dispersion‐corrected (DFT‐D3) method was employed to calculate molecular features and quantum reactivity indices of the peptides with/without O‐glycan functional groups. The structure of the peptides with/without substitutions has been optimised by the B3LYP‐D3BJ functional using the Def2‐SVP basis set in water (Becke 1992; Grimme et al. 2010). The previous studies indicated that the B3LYP functional could provide satisfactory results for the peptide‐based compounds in comparison with other methods such as the hybrid meta‐exchange–correlation functional M06‐2X (Zhao and Truhlar 2008; Roy et al. 2012). The effects of water as a solvent on the geometrical features of the peptides have been considered using the conductor‐like polarisable continuum model (CPCM) (Cossi et al. 1996). To check the structural stability of the corresponding structures and have an estimation of the zero‐point vibrational energies, frequency calculations were performed at the same level. The frequency results indicated the stability of the peptides with real frequencies. All the quantum chemistry calculations were performed using the Gaussian 16 computational package (Frisch et al. 2020).

## 3 Results and Discussion

### 3.1 Functionalised Peptide Complexes With OgpA

**FIGURE 2:**
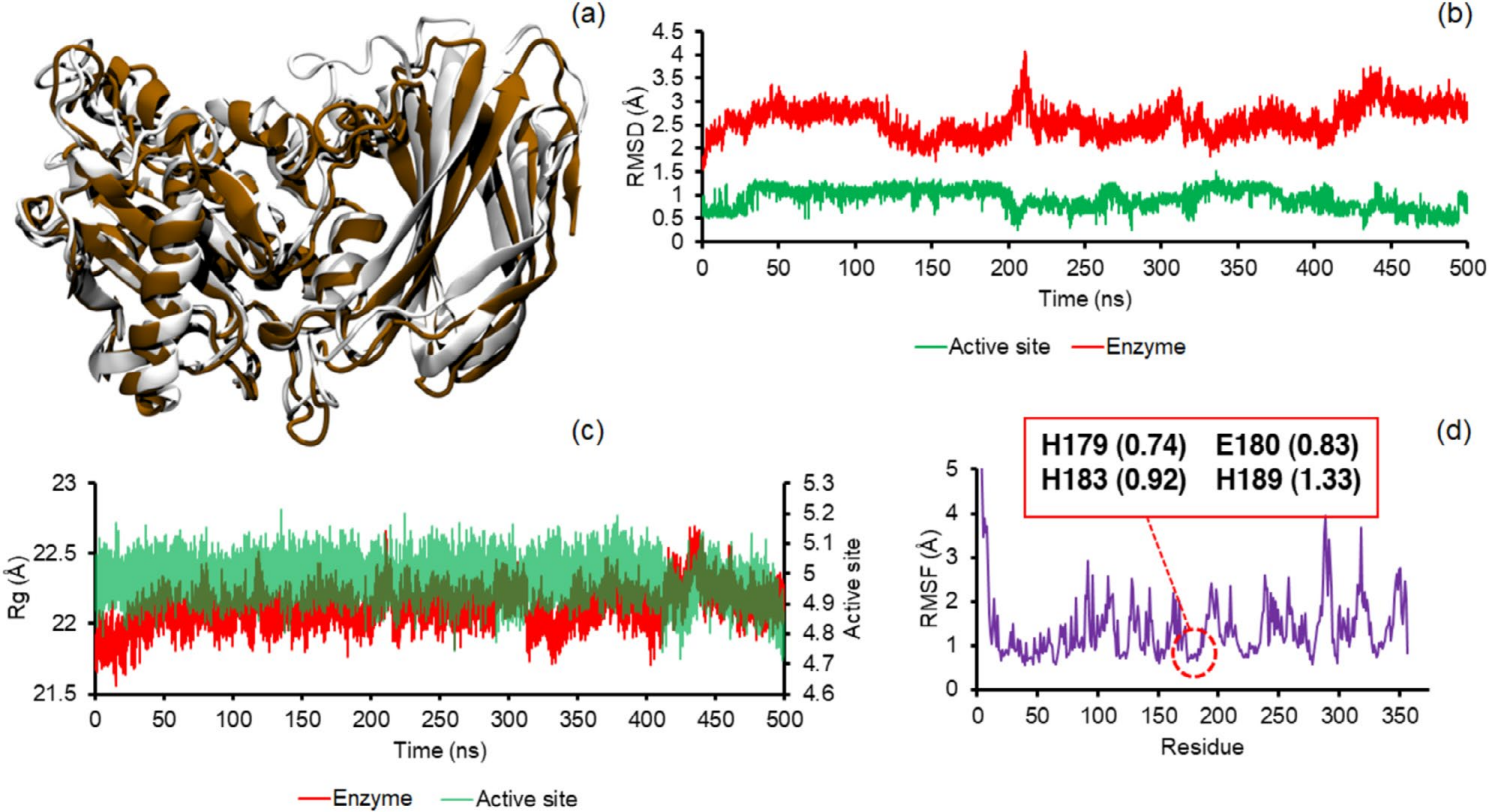
The aligned structure of the OgpA after simulation time (white) to its crystal structure (brown, a) and the calculated RMSD (b) and Rg (c) values for the entire protein and its active site and the calculated RMSF of the protein (d). The RMSF of the involved residues of the OgpA in the active site is highlighted in panel (d).

The *O*‐glycan‐based functional groups of the mucin branches are effective in the activity of the OgpA enzyme. It means that this enzyme selects and degrades specific chains of the mucin based on their *O*‐glycan groups. To analyse the role of the substituted groups of the peptides on the complexation process, the dynamical behaviour of the OgpA in the presence and absence of peptides has been investigated through 500 ns MD simulations. The crystal structure of the OgpA is presented in Figure 1a. According to the experimental results (Trastoy et al. 2020), the active site of this enzyme is composed of His179, Glu180, His183 and His189, which three His residues are coordinated to the Zn^2+^ ion and lie in the centre of the protein approximately. The obtained structure of the OgpA after the simulation time is shown in Figure 2a. According to this figure, the β‐sheet and α‐helix contribute more to the protein's secondary structure (SS). Based on the results, β‐sheet, α‐helix, bend and turn SS are 35%, 32%, 15% and 13% of its skeletal structure, respectively (see Figure S1).

The obtained OgpA structure after simulation time was aligned to the crystal structure of the protein. As can be seen in Figure 2a, the simulated structure does not show significant differences with the crystal structure. The calculated root‐mean‐square deviation (RMSD) for the MD structure relative to the crystal geometry is about 1.61 Å, confirming the stability of the protein along the simulations in water. To check the stability of the OgpA, the RMSD values were computed during 500 ns MD simulations for the entire protein and the active site of the enzyme. As can be seen in Figure 2b, the trend of the calculated RMSD and their small values (average value 2.59 ± 0.31 Å) reveal the structural stability of the protein and confirm that the system reached the equilibrium. Similar to the entire protein, the active site of the enzyme shows remarkable stability and negligible fluctuations. This considerable stability is due to coordinating the His residues to the Zn^2+^ ion, which reduces the flexibility of the active site. The trend of the radius of gyration (Rg) values for both the enzyme and its active site shows a similar result and confirmed an interesting stability during the simulations. Because a greater value of Rg reveals that the protein has lower compactness or more fluctuation in the simulations (Figure 2c). The dynamics of individual residues can be analysed using root‐mean‐square fluctuation (RMSF) values. As can be seen in Figure 2d, Asn92, Asn289, Glu318 and Glu351 of the OgpA show greater RMSF values or fluctuations in comparison with other amino acids. The structural analysis reveals that these residues are on the surface of the protein and higher interactions with solvent molecules can be a possible explanation for this result. In agreement with previous results, RMSF analysis shows stability for the involved residues in the active site. However, the His189 residue presents a higher fluctuation compared with other amino acids of the active site, since this amino acid is in more contact with solvent molecules.

To investigate the stability of the OgpA complexes with different functionalised peptides (Figure 1a), the dynamical behaviour of the designed complexes by docking simulations was analysed during 500 ns MD simulations. As can be seen in Figure S2, the obtained structures for the OgpA complexes after simulation time confirm that all the peptides could make stable complexes with the enzyme and enzyme could maintain the functionalised peptides inside its cavity through a combination of hydrogen bonds (H‐bonds), electrostatic, vdW and π–π interactions. Moreover, the structural analysis confirmed that the conformational features of the peptide inside the binding site cavity depend on the functional groups. In other words, functionalised groups can change the orientation of the peptide relative to the active site.

**FIGURE 3:**
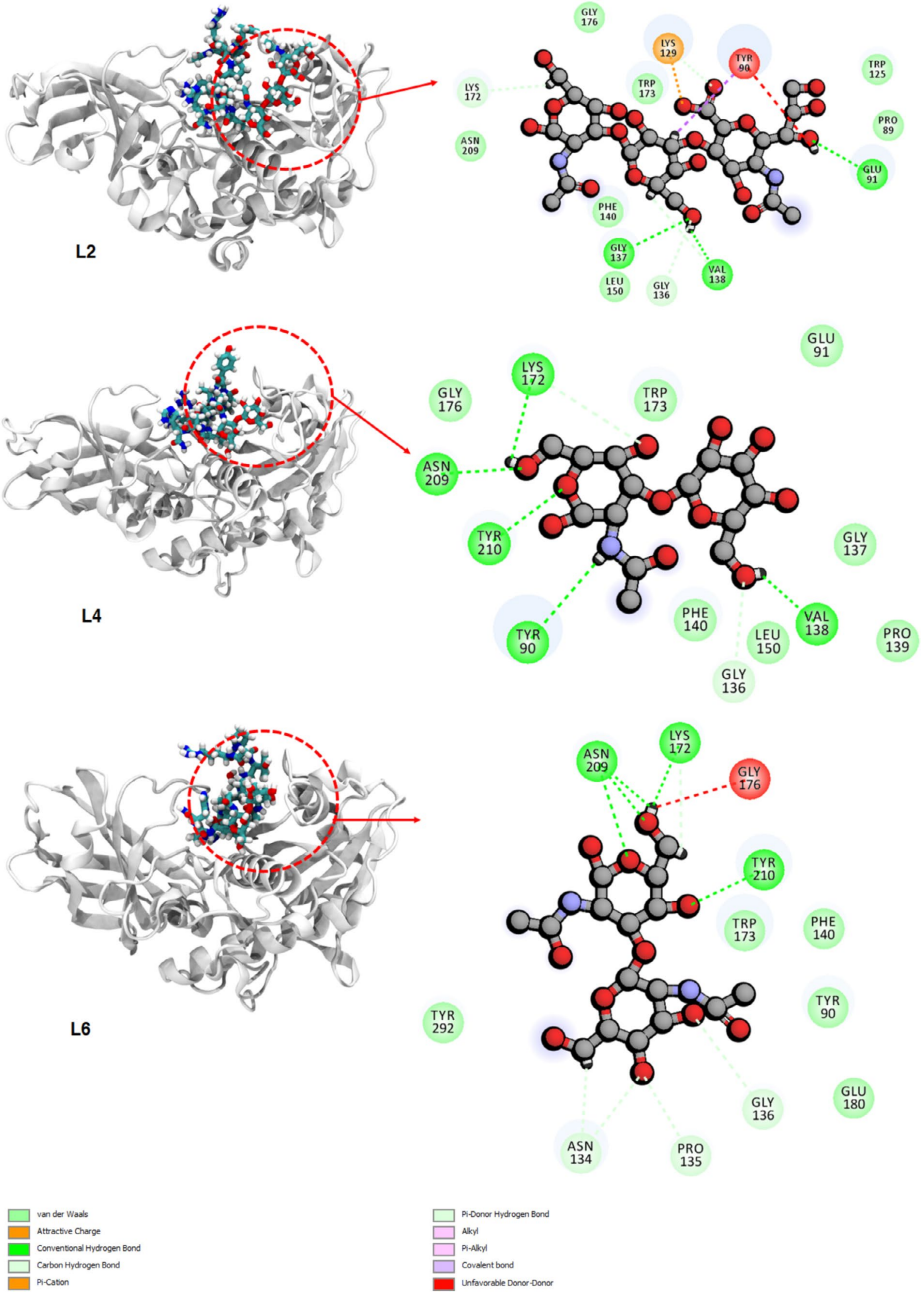
The obtained structures of the OgpA in complexation with the peptide with L2, L4 and L6 functional groups after simulation time and the corresponding binding interaction profiles for each complex. The binding interaction profiles for other *O*‐glycan compounds are presented in the Appendix S1.

To determine the role of the *O*‐glycan functional groups on the complexation process and possible interactions between the peptide and OgpA, the binding interaction profiles are presented in Figure 3 and Figure S3. According to these figures, the functionalised peptides are trapped in the binding cavity through H‐bond, π–π, vdW and electrostatic interactions. Based on the experimental results, the OgpA can degrade peptides with L2, L4 and L6 *O*‐glycan‐based functional groups (see Table 1 and Figure 1d). However, the maximum activity of the enzyme was observed in the presence of a substituted peptide with the L4 group. Therefore, the type of interactions between functional groups and the binding site of the enzyme is crucial to the activity of the OgpA in the hydrolysation process. The carboxyl groups of the L1 compound show strong salt bridge interactions with Lys129 and Lys172 of the OgpA, and there is an unfavourable interaction between the hydroxyl group of the L1 and Tyr90. Moreover, the L1 can form considerable H‐bond interactions with Glu91, Gly137, Asn139, Val138, Tyr210 and Asn209. In addition to these interactions, there are some vdW engagements between L1 and residues of the binding site for instance with Phe140, Pro89, Gly136 and Trp173 that contribute to the stabilisation process. In contrast to the L1 compound, the L2 structure does not form a salt bridge interaction with Lys172; however, this compound shows strong attractive and repulsive interactions with Lys129 and Try90, respectively (see Figure 3). Moreover, the hydroxyl groups of the L2 form some strong H‐bond engagements with Glu91, Val138 and Gly137. The peptide with the L3 functional group is stabilised in the binding site through salt bridge interactions with Lys172 and strong H‐bond interactions with Val138 and Asn209. As can be seen from the obtained binding interaction profile for L3, the vdW interactions are dominated between this compound and the enzyme. The analysis of the interactions between L4 and OgpA indicates that H‐bond and vdW interactions play the main role in the complexation process. The L4 forms five strong H‐bonds with Val138, Tyr90, Tyr210, Lys172 and Asn209, in which Tyr210 and Asn209 behave as hydrogen bond donors in the complexation process. As mentioned, Tyr90 has considerable repulsive interactions with L1 and L2 compounds. However, this residue not only does not have repulsive interactions with the L4 but also forms strong H‐bond interaction with the amine group of the L4. Therefore, it can be considered that Tyr90 interactions with the *O*‐glycan functional group could play an important role in the selectivity of the enzyme. The L5 does not show any H‐bond or electrostatic engagements with the OgpA; this means that this functional group participates in increased vdW interactions between the peptide and the binding site. The hydroxyl and carbonyl groups of the L6 structure form considerable H‐bond interactions with Lys172, Asn209 and Tyr210, and there is a strong repulsive interaction between Gly176 and this compound. The Tyr90 does not form a H‐bond interaction with the L6 in contrast to the L4. This residue in collaboration with other residues such as Trp173, Phe140 and Tyr292 stabilises the substituted peptide with L6 through vdW interactions.

The L7 forms a stable complex with the binding site through vdW and H‐bond interactions, while attractive interaction (salt bridge) between L8 and Lys172 residues plays a key role in the complexation process. In complexation with the L9 structure, Ser207, Asn209, Tyr210 and Glu290 form interesting H‐bond interactions with the hydroxyl and carbonyl groups of this *O*‐glycan. Moreover, other residues including Lys172, Phe140, Gly208, Gly175 and Gly176 participate in the interactions between L9 and the binding site of the OgpA. The L10 (β1,3‐Gal) as the simplest *O*‐glycan participates in the peptide interactions with the enzyme through H‐bond formation with Lys172, Asn209 and Tyr210 along with vdW engagements with Tyr90, Gly175 and Gly176. The binding interaction profile for the L11 structure as the pure peptide (without the *O*‐glycan group) presents considerable vdW, salt bridge, H‐bond and π–π interactions between the amino acids of the peptide and OgpA (see Figure S3). Therefore, it can be considered that the interactions between the functional group of the peptide and binding site play the main role in the selectivity mechanism of the OgpA enzyme, not the interactions between the chains of the mucin and enzyme. In other words, the OgpA selects the mucin chains based on the structural features of their functional groups, and interactions between the substituted chains and the binding site play the main role in the selectivity mechanism of the OgpA. Overall, the binding interaction profiles reveal that Tyr90, Val138, Gly176, Tyr210 and Glu91 residues of the binding site play a more important role in the complexation process compared with other residues.

**FIGURE 4:**
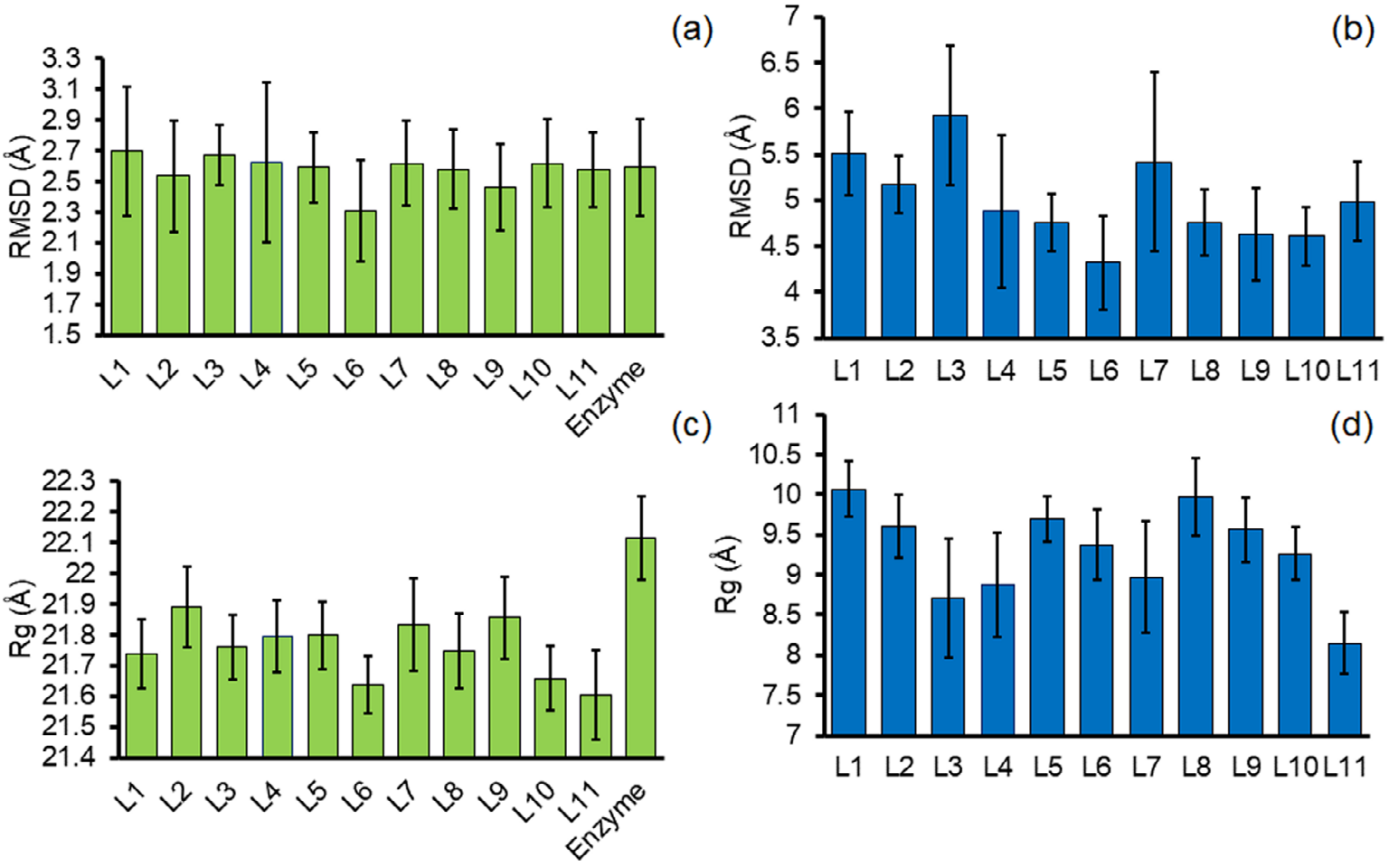
The calculated average RMSD of the OgpA (a) and functionalised peptide with different *O*‐glycan‐based compounds (b) and average values of Rg for the enzyme (c) and corresponding peptides in complexation with the enzyme (d).

The RMSD is one of the important parameters calculated from MD trajectories, which represents interesting results about the structural stability and dynamical behaviour of the enzyme in complexation with the functionalised peptides. The calculated RMSD values of the OgpA (see Figure S4) confirm that the complexation process does not have significant effects on the structural stability of the enzyme, while the computed RMSD of the peptides indicated that the complexation process is effective on their stability (see Figure S4). To have a better comparison of the role of the functionalised groups of the peptide on the stability of the enzyme, the average values of RMSD of the OgpA and functionalised peptides in the presence of each other are reported in Figure 4a,b, respectively. In comparison with the free OgpA, the L2, L6 and L9 reduce the structural fluctuations of the enzyme, confirming that these functionalised peptides form more stable complexes with the receptor than other peptides. However, the calculated difference for the RMSD of the OgpA in the presence and absence of the functionalised peptides is negligible. In contrast to the RMSD values of the enzyme, the computed corresponding values for the peptides reveal considerable differences in the complexation process. As can be seen in Figure 4b, the *O*‐glycan functional group is effective in the stability and dynamical behaviour of the peptide inside the binding cavity. According to the computed results, the peptide with L6 and L3 functional groups has minimum and maximum fluctuations in complexation with the enzyme, respectively. It means that the *O*‐glycan functional groups of the peptides play an important role in the stability of the peptide‐OgpA complexes.

It is possible to check the overall changes in the enzyme structure along the simulations based on the Rg parameter. The consistent trend of the Rg of the enzyme and the peptide in the presence of each other represents the stability of the peptide–enzyme complexes (see Figure S5). Moreover, as can be seen in Figure 4c, the Rg value of the OgpA shows a remarkable reduction in complexation with the substituted peptides. This result indicates that the complexation process reduces the structural flexibility of the enzyme and elevates its compactness. Moreover, the enzyme shows more rigidity in the presence of L6 and L8 in comparison with other selected compounds. In agreement with RMSD results, the Rg values of the peptide revealed that the functional group changes the structural features of the peptide inside the enzyme (see Figure 4d). Based on the computed results, the functionalised peptide with L1, L5 and L8 would have lower structural compactness and higher flexibility inside the binding site of the enzyme.

The individual residue of the OgpA could be important in the interactions between the functionalised peptide and enzyme as well as the mechanism of the degradation process. The fluctuation of each amino acid can be analysed by computing RMSF values, which reveals the average of the fluctuation of the amino acids over the simulation relative to the reference. According to the RMSF results, each amino acid of the OgpA that is involved in the interactions or in direct contact with the peptides has lower fluctuation compared with other amino acids (see Figure S6). Moreover, the fluctuation of the entire protein shows a considerable reduction in the complexation form to the free state. Furthermore, the calculated RMSF values of the residues in the active site have significantly reduced in the presence of the functionalised peptides. It confirms that there are considerable interactions between Glu180, His179, His183 and His189 residues and the *O*‐glycan functionalised peptides.

**FIGURE 5:**
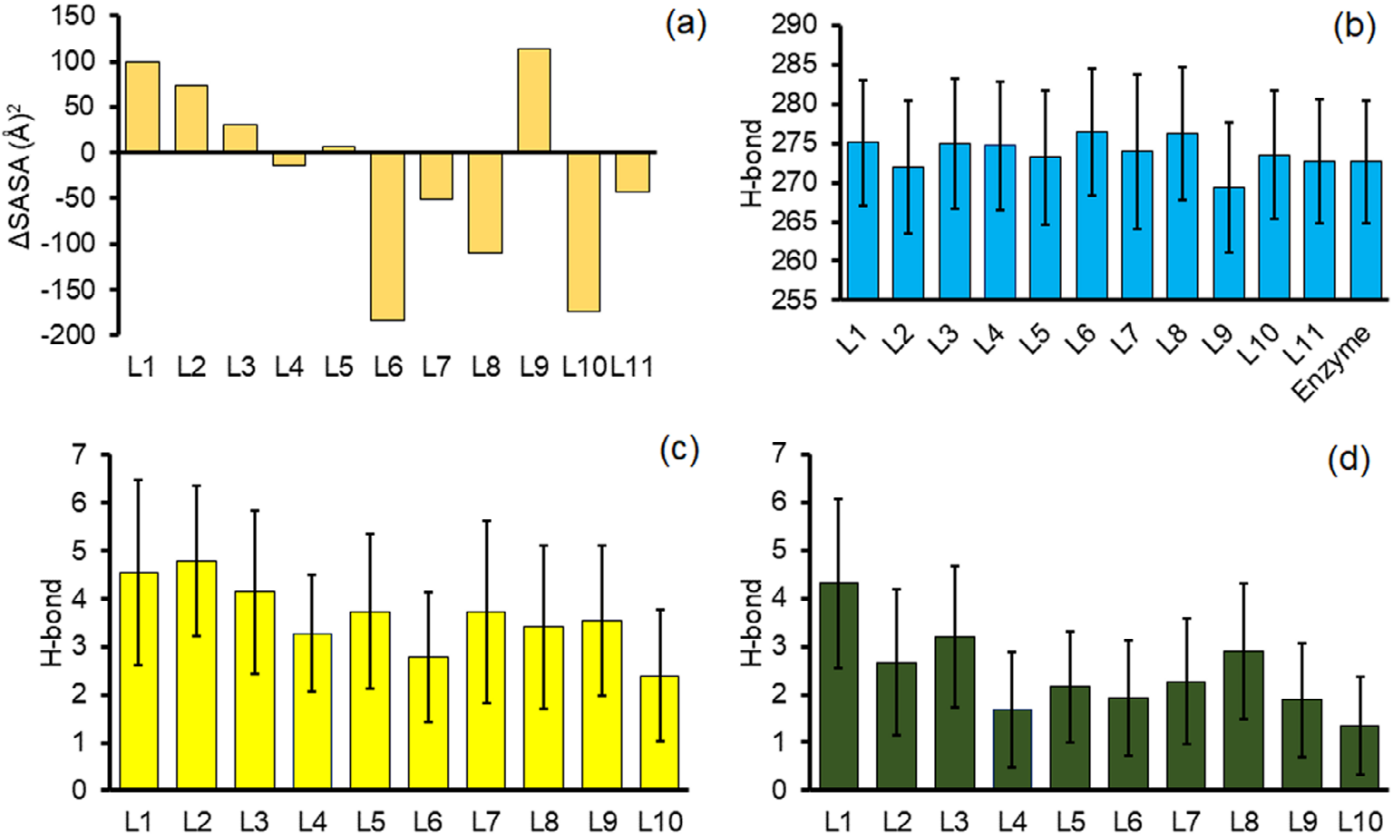
The calculated SASA values of the OgpA in complexation with the peptides relative to the free OgpA (a) and the calculated average internal H‐bond of the enzyme (b), H‐bond interactions between O‐glycan groups (donor) and residues of the enzyme (acceptor, c), and H‐bond interactions between corresponding compounds and enzyme, in which enzyme's residues behave as donors (d).

In the absence of the peptide, the binding site of the enzyme has been occupied by water molecules. Therefore, it is predictable that there is a competition between water molecules and peptides to interact with the enzyme. As can be seen in Figure 5a, the solvent‐accessible surface area (SASA) of the OgpA shows differences in facing different peptides. According to this figure, the accessible surface of the enzyme has been increased by L1, L2, L3 and L9, while other functionalised peptides with L6, L7, L8 and L10 have a contrast effect on the enzyme–water interactions. It is well worth mentioning that the SASA values of the enzyme show considerable reduction even in the presence of small *O*‐glycan‐based compounds like L10, meaning that the complexation process can change the structural features and conformational properties of the enzyme. The H‐bond interaction is not only important for the structural stability of the enzyme but also effective in the stability of the OgpA–peptide complexes and can be considered as a driving force for the complexation process. The internal H‐bond interactions of the OgpA have been decreased by complex formation with L9 and L2 compared with the free enzyme. This result confirms that *O*‐glycan groups can play an effective role in the internal interactions of the enzyme (see Figure 5b). The functional groups in this study can behave as donors or acceptors in H‐bond formation. The average H‐bond engagements between functional groups (donor) and residues of the OgpA (acceptor) are reported in Figure 5c. According to this figure, L2 and L10 have maximum and minimum H‐bond interactions with the OgpA, respectively. In other words, L1, L2 and L3 are better H‐bond donor compounds for complex formation with the binding site. Moreover, the obtained results represent that L1 and L3 *O*‐glycans have more capacity as H‐bond acceptors than other compounds (see Figure 5d). In other words, compared with other *O*‐glycans, the L1 and L3 are more desirable to form H‐bond interactions with the OgpA.

Overall, the obtained results indicated that among the selected *O*‐glycans, there are some candidates that show more affinity to the OgpA enzyme than the L4 compound, but experimental results reported that this enzyme has maximum activity against the mucin chains with the L4 functionalised groups. To make a connection between experimental and computational results, we can consider that the structural feature of the functional group is an important factor in the selectivity of the enzyme. On the other hand, some *O*‐glycans form stable complexes with the OgpA to some extent that they behave as inhibitors against the enzyme. The OgpA exhibits the ability to hydrolyse peptides featuring L2, L4 and L6 functional groups. This capability arises from the fact that these *O*‐glycans possess a diminished affinity for the enzyme's binding site. Consequently, the resulting degradation compounds can readily leave the active site in comparison to other *O*‐glycans.

### 3.2 Role of the *O*‐Glycan on the Peptide–OgpA Interactions

**FIGURE 6:**
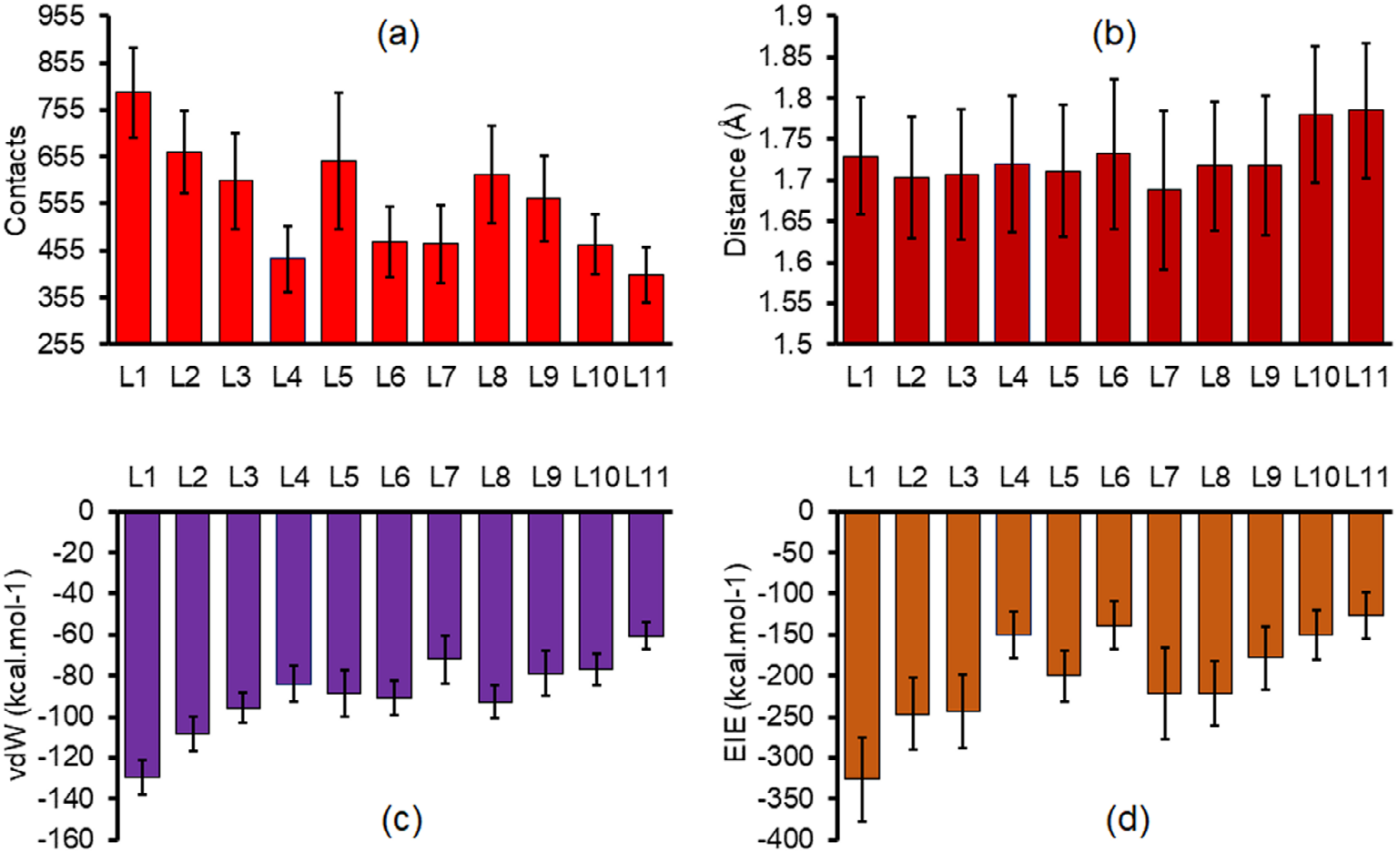
The calculated average number of contacts (a), minimum distance (b), vdW interactions (c) and electrostatic interactions (d) between peptides with/without *O*‐glycan groups and the OgpA enzyme. A 4 Å cutoff was applied for calculating the number of contact values in panel a.

To investigate the role of the *O*‐glycan groups on the affinity of the OgpA against the peptide, the possible interactions during complex formation were investigated. The number of contacts between the peptide and the binding site of the OgpA can be a criterion for the affinity of the enzyme towards the functionalised peptide. As can be seen in Figure 6a, in comparison with the naked peptide, functional groups play a pivotal role in the peptide–OgpA interactions. On the basis of the obtained results, all the *O*‐glycan groups increase the affinity of the enzyme against the peptide, in which a greater affinity was observed in the presence of the L1, L2, L5 and L8 groups. Compared with other functional groups, the L4‐substituted peptide is in less contact with the active site of the enzyme, in agreement with previous results. According to the calculated number of contacts, the peptides with L4, L6 and L2 lie in the first, second and third ranks with minimum interaction with the OgpA, respectively. As mentioned before, the experimental data confirmed that the activity of the enzyme against the functionalised peptides in the degradation process is as follows: L4 > L6 > L2. Therefore, the OgpA shows greater activity against the peptides with these functional groups because these *O*‐glycans can leave the active site of the enzyme after the degradation faster due to a lower affinity against the enzyme, compared with other groups such as L1 and L5. The calculated minimum distance values between the *O*‐glycan and the active site are in the range of electrostatic and H‐bond interactions. As can be seen in Figure 6b, the naked peptide has the maximum distance from the binding site of the enzyme. In other words, the functionalisation process decreases the distance between the peptide and the enzyme. Therefore, the functionalised peptides are subjected to more interactions with the active site of the enzyme.

The enzyme traps the peptide inside its cavity through electrostatic and vdW interactions. The linear interaction energy (LIE) method was applied to calculate electrostatic (ELE) and vdW interactions between peptides with/without *O*‐glycans and the OgpA. The obtained results significantly reveal that *O*‐glycans elevate the vdW interactions inside the peptide–enzyme complexes because the functionalisation process promotes π–π and other noncovalent engagements (see Figure 3 and Figure S3). Moreover, the peptide with L1 shows maximum vdW interactions because this functional group is in greater contact with Pro89, Trp135, Leu150, Phe140 and Trp173 compared with other *O*‐glycans (see Figure 6c). Furthermore, in comparison with the naked peptide, the L4 and L7 groups do not have remarkable effects on the peptide's vdW interactions, in contrast to L1, L2 and L8. The obtained electrostatic interaction energy values for the peptide–enzyme complexes indicate that electrostatic interactions dominate in the complexation process (see Figure 6d). The calculated ELE values represent that the peptide with L6 and L4 *O*‐glycans has lower electrostatic interactions with the enzyme, in agreement with previous results. As can be seen in Figure 6d, the L1 group increases the electrostatic interactions inside the peptide–enzyme complex at least two times in comparison with the naked peptide. Because the peptide in the presence of this *O*‐glycan forms two strong salt bridge interactions with Lys172 and Lys129 residues. The L4 and L6 groups have lower electrostatic interactions with the enzyme because the binding interaction profile (Figure 3) does not show any salt bridge interactions and confirms that complexes of these *O*‐glycans stabilise through vdW and H‐bond interactions.

**FIGURE 7:**
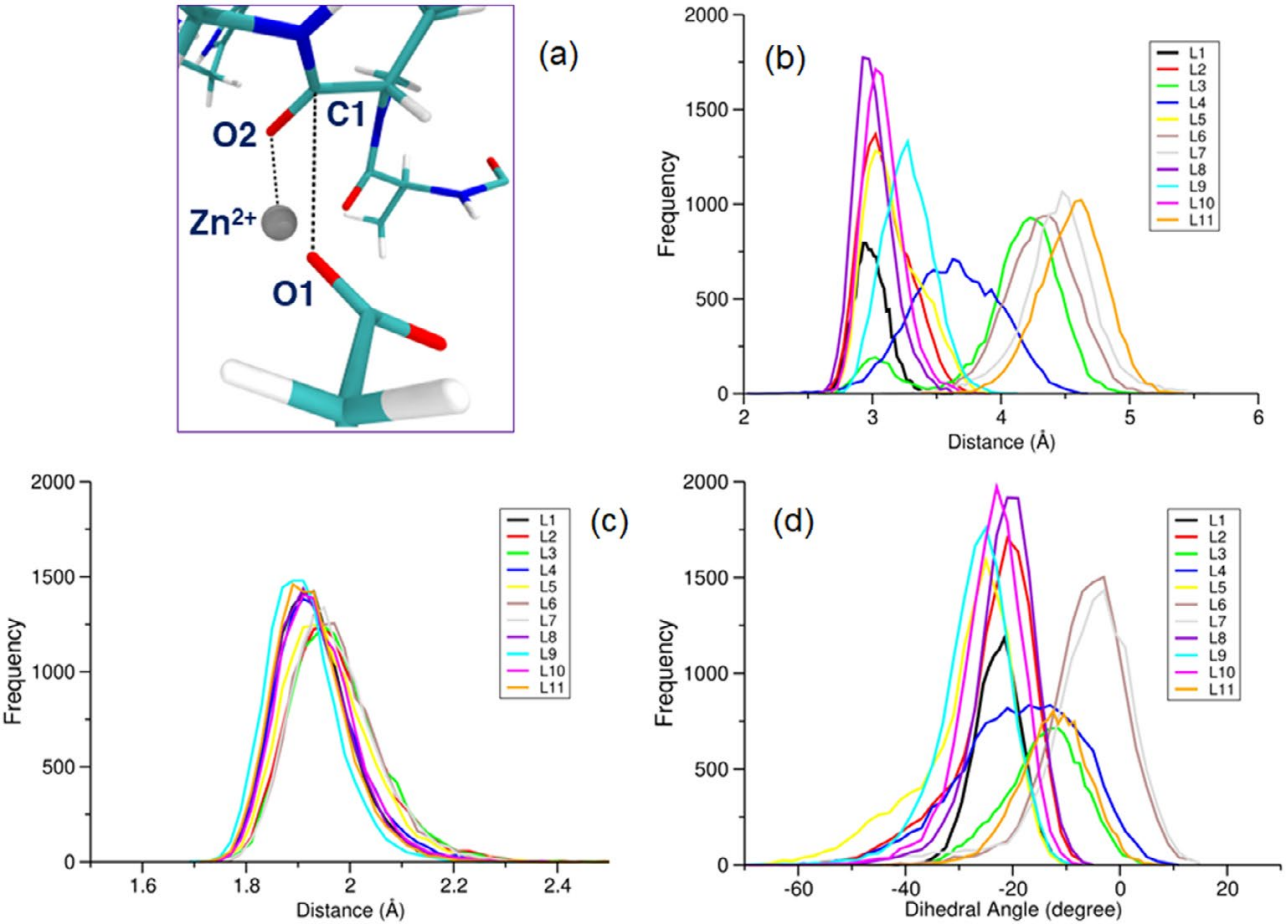
A presentation of the interactions between O1 (Glu180) and C1 (Thr residue of the peptide) atoms that play a key role in the degradation process (a). The calculated frequency of the observed C1‐O1 distance (b), O2‐Zn^2+^ distance (c) and O1‐C1‐O2‐Zn^2+^ dihedral angle (d) of the peptide interactions (with/without functional groups) with the active site of the OgpA.

The mucin degradation process starts with the interaction between the COO^−^ group of the Glu180 (active site) and the CO group of the functionalised Thr residue with *O*‐glycans. Therefore, the orientation and distance between these groups inside the active site for starting the degradation process are of importance. In this context, the role of the functional groups on the distance between COO^−^ (Glu180) and CO (Thr of the peptide) and their dihedral angle relative to each other was investigated (see Figure 7a). On the basis of the obtained results, the O1‐C1 distance (O1 of Glu180 and C1 of Thr residue of the peptide) has been affected by the type of the functional group. In other words, the *O*‐glycan groups play a pivotal role in the interactions between the involved residues in the centre of the degradation reaction. As can be seen in Figure 7b, the functionalisation process reduces the C1‐O1 distance because the CO group of the naked peptide has the maximum distance relative to the Glu180 residue. It means that *O*‐glycan groups facilitate the degradation process. The calculated C1‐O1 distance for the naked peptide is about 4.55 Å, which reduces to 2.99 Å in the presence of the L1 group. Based on Figure 7b, the observed frequency distribution of C1‐O1 distances varies in the presence of various *O*‐glycans, displaying two distinct peaks approximately at 3.0 and 4.5 Å. Notably, the C1‐O1 distance for the peptide with the L4 group falls within this range, forming a distinct peak at the midpoint. Therefore, we can define a theoretical criterion, meaning that there are some *O*‐glycans that increase the affinity of the peptide against the enzyme to the extent that they behave as inhibitors, while there are other candidates with a contrast effect. Since the L4 *O*‐glycan lies in the middle range or optimum condition, we observe the maximum activity for the enzyme in the presence of this compound.

Another pivotal interaction in the degradation process is O2‐Zn^2+^ (O2 of the CO group of the Thr residue). Our obtained results indicate that the *O*‐glycan group can be effective on the O2‐Zn^2+^ distance (see Figure 7c). Some candidates such as L7, L3 and L2 reduce the oxygen interactions with the Zn^2+^ ion, while other groups including L9 and L8 show a contrast effect, compared with the naked peptide. In comparison with the O2‐Zn^2+^ distance, the functional groups are more effective in the C1‐O1 interaction, which is crucial as the first step of the reaction. In addition to the distance between the Thr residue and the active site of the enzyme, the orientation of the *O*‐peptide bond relative to the residues of the active site could be important from the energetic viewpoint. The calculated dihedral angle for the O1‐C1‐O2‐Zn^2+^ (Glu and Thr residues) of the naked peptide is about −12.0°, which changes to 0.0° and −30.0° in the presence of L7 and L9 *O*‐glycan groups, respectively (see Figure 7d). The corresponding dihedral angles for the peptide with L2, L4 and L6 are −18.0°, −22.0° and −6.0°, respectively. Therefore, it can be considered that the optimum distance and angles between Glu180 and the functionalised Thr residue of the peptide are 3.64 Å and −22.0°, respectively. Because the enzyme shows maximum activity in the presence of this functionalised peptide.

**FIGURE 8:**
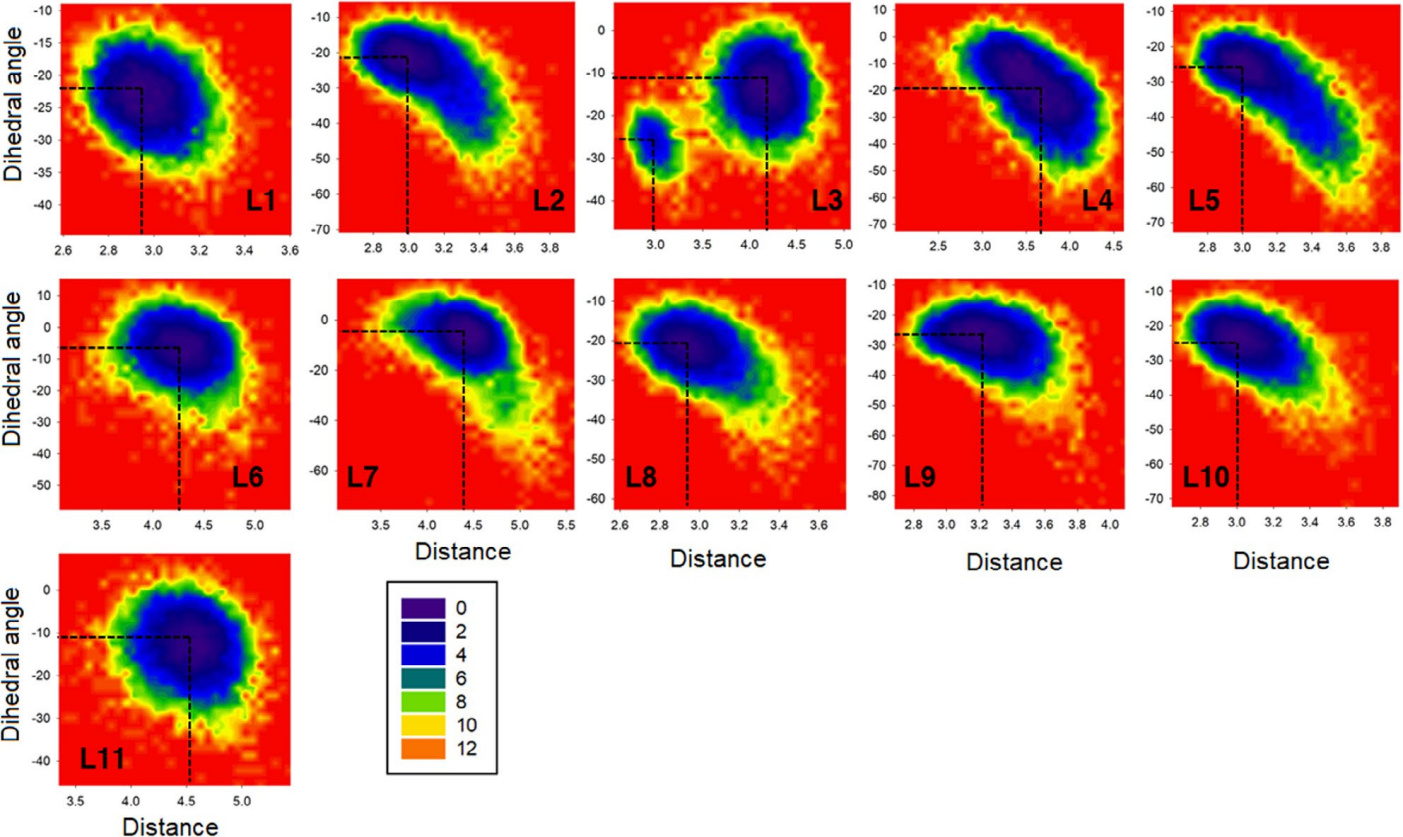
The calculated FELs for the functionalised peptides with different *O*‐glycan groups as a function of the C1‐O1 distance (Å) and O1‐C1‐O2‐Zn^2+^ dihedral angle.

The changes in the conformational properties of the functionalised peptides revealed that the dynamical behaviour has been affected by the type of the *O*‐glycan groups. To investigate the role of the *O*‐glycan groups on the stability and dynamical features of the peptide inside the binding cavity of the enzyme, free‐energy landscape (FEL) analysis has been applied (Moritsugu, Terada, and Kidera 2017). By employing this method, the role of the *O*‐glycans on the peptide interactions with the active site as a function of the C1‐O1 distance and O1‐C1‐O2‐Zn^2+^ dihedral angle was investigated. As can be seen in Figure 8, the shape of the FELs has been changed in the presence of different *O*‐glycans, which indicates that these groups play a key role in the peptide‐active site (Thr–Glu180) interactions. The changes in the FELs reveal that the affinity of the OgpA to the peptides is different and each functionalised peptide has unique dynamics inside the active site. Based on the minimum point of the free‐energy profile, it is possible to determine the most stable configuration along the simulation time. The obtained free‐energy profiles indicate that functionalised peptides have many conformational states in complexation with the enzyme. The energy profile of the L3 shows two minimum energy points at the distance of 3.0 and 4.3 Å relative to the Glu180 residue. This result illustrated that the peptide with the L3 group has lower stability of greater fluctuation inside the binding cavity in comparison with other functionalised peptides. Based on the calculated FELs, the peptides with L2, L4 and L6 *O*‐glycans have the maximum interactions with the active site at the distance of 3.10, 3.60 and 4.20 Å (Thr–Glu180 distance).

Since the complexation process changes the structural features of the enzyme, SS analysis was applied to investigate the role of the functional groups on the structural features of the OgpA. To have a better comparison, the percentage of each SS of the OgpA is reported in Figure S7 in the presence and absence of the peptides. The contribution of the β‐strands in the SS of the enzyme does not show a significant difference in complexation form compared with the free enzyme, while the β‐bridge SS shows a contrasting result. The notable point is that the contribution of the π‐helix SS reduces due to the complexation process with all the functionalised peptides except in the presence of a peptide with L8 *O*‐glycan. Moreover, the π‐helix, β‐bridge and α‐helix SSs of the OgpA have changed to α‐3‐10 structures in complexation with L2, L4 and L6 functionalised peptides. The SS analysis reveals that the complexation process changes the SS of the enzyme to α‐3‐10 and Turn structures. Since the SS of the OgpA changes in the presence of each *O*‐glycan, it is predictable that the enzyme takes a specific configuration against each functionalised peptide, which could be effective in the activity of the enzyme. It means that structural features of the *O*‐glycan are important in the conformational properties of the enzyme and can be considered as a factor in the activity of the enzyme and the enzyme's selectivity mechanism in the degradation process.

### 3.3 Complexation Energy

To study the role of the *O*‐glycan groups of the peptide on the stability of the peptide–enzyme complexes, the complexation energies were calculated using the MM‐GBSA method. The calculated vdW interaction energies (ΔE_vdW_) reveal that *O*‐glycan groups change the stability of the corresponding complexes. According to Table 2, the peptide with L1 and L7 has the maximum and minimum vdW interactions with the enzyme, respectively, in comparison with the naked peptide. Among the L2, L4 and L6, the L4 functionalised peptide shows lower affinity towards the OgpA based on ΔE_vdW_ values, in agreement with previous results. Some functional groups such as L10, L6 and L4 reduce the electrostatic interaction between the peptide and binding site of the enzyme, while other *O*‐glycan groups including L1, L2, L3 and L8 improve the stability of the corresponding complex through promoting electrostatic interactions. This result is due to the strong salt bridge formation between these *O*‐glycans and Lys129 and Lys172 residues of the binding cavity. Both MM‐GBSA and LIE methods provide a similar trend for the role of the *O*‐glycans on the electrostatic and vdW interactions between the peptide and OgpA. The stability of the corresponding complexes can be verified from the thermodynamic viewpoint based on the calculated Gibbs binding energies in the gas phase (ΔG_gas_). Moreover, the L1 functionalised peptide forms the most stable complex with the OgpA compared with other functionalised peptides. The calculated Gibbs solvation energies (ΔG_sol_) represent that solvent decreases the stability of the peptide–enzyme complexes, confirming the remarkable role of the water molecules in the interactions between peptides and the enzyme. The obtained total Gibbs binding energies (ΔG_tot_) confirm that the stability of the peptides inside the active site depends on their *O*‐glycan functional groups. The *O*‐glycan groups play a key role in the stability of the complexes. For example, functionalising the naked peptide with L1 *O*‐glycan elevates the stability of the system up to three times. Moreover, the substituted peptides with L2, L4 and L6 show considerable stability according to the computed ΔG_tot_ values (−93.03, −64.55 and −59.35 kcal.mol^−1^, respectively). In summary, the calculated complexation energies demonstrate that the enzyme's affinity towards the peptide varies depending on the functional groups. This variability arises from the significant impact of functional groups on both the conformational characteristics of the peptide within the binding cavity and the orientation of the peptides relative to the active site.

**TABLE 2:** The calculated thermodynamic parameters (kcal.mol^−1^) for the peptide–enzyme complexes using the MM‐GBSA method.

*O*‐glycan	ΔE_vdW_	SD	ΔE_ele_	SD	ΔG_gas_	SD	ΔG_sol_	SD	ΔG_tot_	SD
L1	−135.50	6.27	−287.61	32.62	−423.12	32.58	309.48	28.17	−113.64	9.29
L2	−114.31	7.43	−184.85	29.69	−299.16	30.52	206.13	25.58	−93.03	9.47
L3	−105.80	6.02	−237.92	22.59	−343.72	22.45	252.63	20.78	−91.08	7.06
L4	−86.77	7.57	−60.61	20.72	−147.38	21.42	82.83	18.67	−64.55	7.68
L5	−89.58	10.11	−142.29	22.93	−231.83	22.77	161.03	21.24	−70.80	10.28
L6	−94.91	7.00	−70.52	30.93	−165.44	31.45	106.09	28.36	−59.35	7.73
L7	−72.45	8.63	−155.75	35.54	−228.21	36.25	181.50	30.56	−46.70	10.32
L8	−101.41	9.00	−214.71	28.13	−316.12	27.39	242.26	26.89	−73.86	11.35
L9	−73.88	8.98	−135.52	44.54	−209.40	44.32	158.86	38.21	−50.54	10.53
L10	−87.21	7.57	−46.32	20.11	−133.54	21.40	71.18	18.27	−62.35	9.22
L11	−65.22	5.78	−80.19	36.29	−145.41	36.67	96.52	32.17	−48.89	7.18

These results are in agreement with several previous studies (Medley et al. 2022; Taleb et al. 2022) that have reported similar analyses of mucinases from other mucus‐degrading bacteria, providing a basis for comparison with OgpA. For instance, mucinases such as BT4244 from *Bacteroides thetaiotaomicron* and AM0627 from *Akkermansia muciniphila* have been structurally and mechanistically characterised, revealing their ability to cleave mucin *O*‐glycans with specificity for particular glycan structures (Taleb et al. 2022). Like OgpA, these enzymes exhibit a preference for bis‐T/Tn substrates and employ conserved catalytic mechanisms involving key residues such as Thr and a nucleophilic water molecule. Structural studies have also identified conserved features, such as sugar–π interactions mediated by tyrosine residues, which are critical for substrate recognition. These findings highlight a shared strategy among mucinases from different bacteria in recognising and processing *O*‐glycan‐decorated mucins, enabling a meaningful comparison of OgpA with its analogues.

### 3.4 Role of the *O*‐Glycan on the Chemical Reactivity of the Peptide

According to the obtained results from MD simulations, *O*‐glycan groups have considerable effects on the peptide–enzyme complexation energies as well as the affinity of the enzyme against the functionalised peptides. Therefore, to determine the role of the *O*‐glycans on the chemical reactivity of the peptide in complexation with the OgpA, DFT‐D3 calculations at the B3LYP‐D3BJ/Def2‐SVP level of theory have been employed. The optimised coordinates of the functionalised peptide with different *O*‐glycans are reported in the Appendix S1. Since the peptides were optimised in the absence of the OgpA, their conformational features show some difference compared with the complexation state. However, the optimised structures clearly reveal that *O*‐glycan groups are effective on the structural properties and conformational features of the peptides.

The frontier molecular orbital (FMO) method was employed to determine the quantum reactivity indices of the functionalised peptides including the band‐gap energy (η), electronic chemical potential (μ) and global electrophilicity index (ω). The FMO method provides valuable data in quantum chemistry computations. It focuses on the analysis of the highest occupied molecular orbital (HOMO) and the lowest unoccupied molecular orbital (LUMO) (Izadyar, Khavani, and Housaindokht 2017). HOMO and LUMO orbitals are linked to the capacity for donating and accepting electrons, respectively. The computed HOMO and LUMO energies (E_HOMO_ and E_LUMO_) represent the role of the *O*‐glycan substitutions and a remarkable difference for the functionalised peptides compared with the naked one (Table 3). Since the selected *O*‐glycans have different electronic charges and chemical functional groups, the *O*‐glycan‐peptide's molecular orbitals exhibit distinct electron distribution and orbital energy levels than the naked peptide. Electronic transitions in molecular systems typically take place from the HOMO to LUMO as any alternative transitions require more energy than the difference between the HOMO and LUMO energy levels (η = E_LUMO_—E_HOMO_), which increasing this energy difference reduces the reactivity of the molecule. According to the obtained η values, the reactivity of the naked peptide has increased significantly in the presence of L1 and L3 *O*‐glycans. It means that a peptide with these functional groups exhibits a greater affinity towards the OgpA compared with other *O*‐glycans. There is an interesting linear correlation between the calculated η values by the DFT‐D3 method and ΔE_ele_ obtained from MM‐GBSA calculations (see Figure S8). According to the obtained results, by increasing the η values of the peptides, the electrostatic interactions between functionalised peptides and the OgpA reduce. It means that the affinity of the enzyme towards the peptide or branches of mucin in the degradation mechanism significantly depends on the nature of the *O*‐glycan functional groups.

**TABLE 3:** The calculated quantum reactivity indices (eV) of the peptides with/without *O*‐glycans at the B3LYP‐D3BJ/Def2‐SVP level of theory.

*O*‐glycans	E_HOMO_	E_LUMO_	η	μ	ω
L1	−5.531	−0.745	4.786	−3.138	1.029
L2	−5.880	−0.666	5.214	−3.273	1.027
L3	−5.531	−0.621	4.910	−3.076	0.964
L4	−6.147	−0.639	5.509	−3.393	1.045
L5	−6.223	−0.780	5.443	−3.501	1.126
L6	−6.285	−0.626	5.659	−3.455	1.055
L7	−5.854	−0.688	5.166	−3.271	1.036
L8	−5.886	−0.690	5.196	−3.288	1.041
L9	−6.127	−0.691	5.436	−3.409	1.069
L10	−6.222	−0.776	5.446	−3.499	1.124
L11	−6.199	−0.591	5.607	−3.395	1.028

The electronic chemical potential (μ) is another chemical reactivity parameter that indicates the molecule's propensity to acquire an extra electronic charge. The L2 and L6 *O*‐glycans are more effective on the electronic chemical potential of the peptide than the L4 group. The electrophilicity index (ω) quantifies the extent of stabilisation energy gained by the system when it acquires an extra charge from the surrounding environment. The calculated ΔG_tot_ values indicate that L5 and L10 groups reduce the stability of the peptide–enzyme complexes compared with the L10 *O*‐glycan. On the other hand, the peptide with L5 and L10 exhibits a greater electrophilicity character than with L1, meaning that the OgpA enzyme shows more affinity against a peptide with a lower electrophilicity character. Overall, DFT‐D3 calculations illustrate that *O*‐glycan groups not only change the structural features of the peptide but also are effective on the peptide's reactivity, which plays a key role in the affinity of the enzyme against the peptide in the degradation process.

## 4 Conclusion

In this article, MD simulations and quantum chemistry calculations were applied to investigate the role of the *O*‐glycan groups on the affinity of the OgpA enzyme against a peptide as a model of the mucin branches. The obtained results indicated that among the selected *O*‐glycans, there are some candidates that show more affinity to the OgpA enzyme than the L4 compound, but experimental results reported that this enzyme has maximum activity against the mucin chains with the L4 functionalised groups. Therefore, we can consider that the structural feature of the functional group is an important factor in the selectivity of the enzyme. On the other hand, some *O*‐glycans form stable complexes with the OgpA to some extent that they behave as inhibitors against the enzyme. The OgpA exhibits the ability to hydrolyse peptides featuring L2, L4 and L6 functional groups. This capability arises from the fact that these *O*‐glycans possess a diminished affinity for the enzyme's binding site. Consequently, the resulting degradation compounds can readily leave the active site in comparison to other *O*‐glycans. Moreover, the obtained results indicated that L1 and L3 *O*‐glycans have more capacity as H‐bond acceptors than other compounds. In other words, compared with other *O*‐glycans, the L1 and L3 are more desirable to form H‐bond interactions with the OgpA. The functionalisation process decreases the distance between the peptide and the enzyme, meaning that the functionalised peptides are subjected to more interactions with the active site of the enzyme. The enzyme maintains the functionalised peptides inside its cavity through electrostatic and vdW interactions, in which the electrostatic interactions dominate the complexation process. According to the DFT‐D3 results, by increasing the chemical hardness values of the peptides, the electrostatic interactions between functionalised peptides and the OgpA reduce. It means that the affinity of the enzyme towards the peptide or branches of mucin in the degradation mechanism significantly depends on the nature of the *O*‐glycan functional groups.

It is well worth mentioning that this study employed computational techniques to provide atomic‐level insights into the interactions between *O*‐glycan groups and the OgpA enzyme. These in silico methods are powerful tools for exploring enzyme mechanisms, revealing critical details about molecular selectivity and binding that are difficult to capture experimentally. The ability to simulate multiple *O*‐glycan functional groups in complex environments provides valuable hypotheses to guide future experimental studies. These approaches provide critical insights into the structural and energetic factors influencing enzyme selectivity, complementing experimental techniques. While computational models inevitably involve simplifications, such as the use of specific force fields and parameters, these are carefully chosen to ensure reliability and relevance to the biological system under study. By focusing on specific functional groups and their interactions with OgpA, this study highlights how computational approaches can drive hypothesis generation and enhance our understanding of complex biological processes.

Moreover, the findings of this study have significant potential for biotechnological applications. Understanding the molecular mechanisms of mucin degradation by gut microbiota enzymes like OgpA can contribute to the development of innovative therapeutic strategies targeting the gut microbiome. Modulating OgpA activity, for instance, could serve as a means to manage dysbiosis‐related conditions or enhance mucosal health. Furthermore, the ability of certain *O*‐glycans to inhibit OgpA suggests opportunities to design enzyme inhibitors that protect the mucosal barrier in diseases characterised by excessive mucin degradation. Beyond therapeutic applications, these insights could be harnessed in biotechnological fields such as drug delivery, where enzymes like OgpA might be engineered for site‐specific mucin degradation, improving the efficacy of drug formulations. Additionally, these findings could aid in designing prebiotic or probiotic strategies that promote beneficial microbial activity in the gut, contributing to overall host health.

## Author Contributions

**Mohammad Khavani:** conceptualization, investigation, methodology, validation, visualization, writing – review and editing, software, formal analysis, data curation, writing – original draft. **Aliyeh Mehranfar:** methodology, visualization, writing – review and editing, writing – original draft, validation, software, formal analysis. **Mohammad R. K. Mofrad:** conceptualization, software, project administration, supervision, resources, investigation, funding acquisition, writing – review and editing, validation, formal analysis, methodology, visualization.

## Conflicts of Interest

M.R.K.M. is a co‐founder of Nexilico Inc., a start‐up developing AI‐driven microbiome engineering technologies. Other co‐authors declare no conflicts of interest.

## Supporting information


Appendix S1

## Data Availability

Data sharing is not applicable to this article as no datasets were generated or analysed during the current study.
