# Supplementary material for: Unravelling the Glycan Code: Molecular Dynamics and Quantum Chemistry Reveal How O‐Glycan Functional Groups Govern OgpA Selectivity in Mucin Degradation by Akkermansia muciniphila
Source: Microb Biotechnol. 2025 Apr 3;18(4):e70091. doi: 10.1111/1751-7915.70091 (PMC11968330; doi:10.1111/1751-7915.70091)
Supplement: Supplementary file 1 — Appendix S1 [file MBT2-18-e70091-s001.docx]

**Supporting Information**

**Unraveling the Glycan Code: Molecular Dynamics and Quantum Chemistry Reveal How O-Glycan Functional Groups Govern OgpA Selectivity in Mucin Degradation by *Akkermansia muciniphila***

Mohammad Khavani­, Aliyeh Mehranfar, Mohammad R. K. Mofrad*

Molecular Cell Biomechanics Laboratory, Departments of Bioengineering and Mechanical Engineering, University of California Berkeley, Berkeley, California 94720, USA

[mofrad@berkeley.edu](mailto:mofrad@berkeley.edu)

**
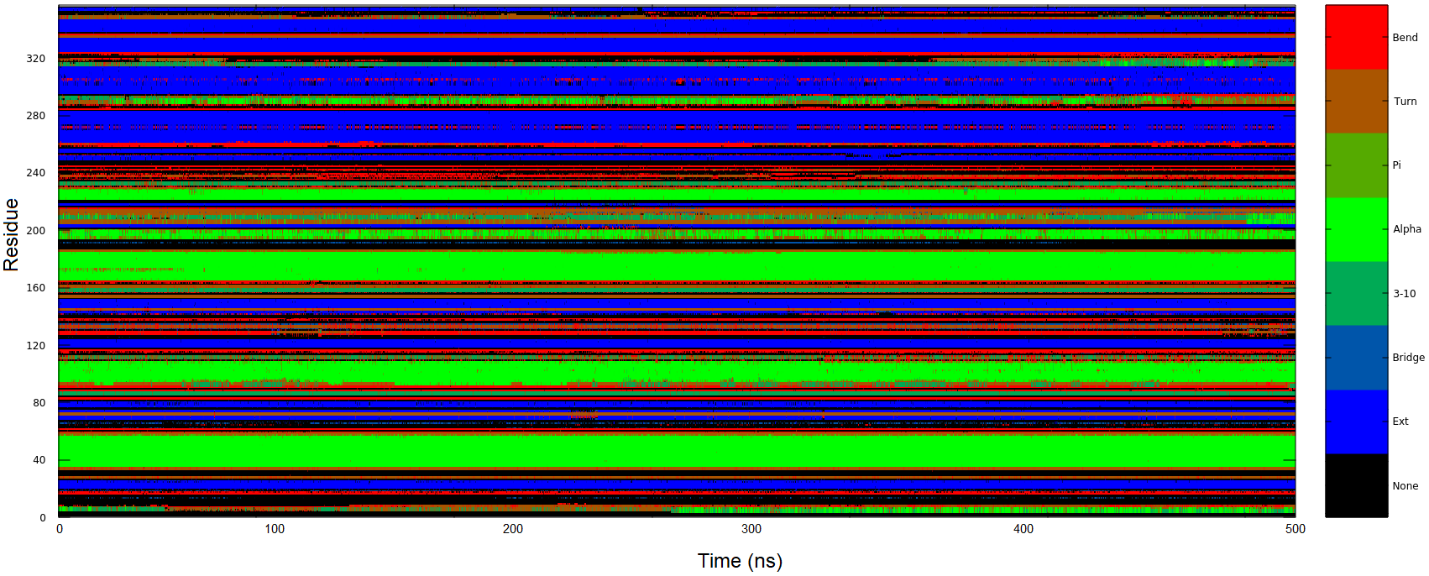
**

Figure S1. The calculated secondary structure of the free OgpA during 500 ns MD simulations in water.


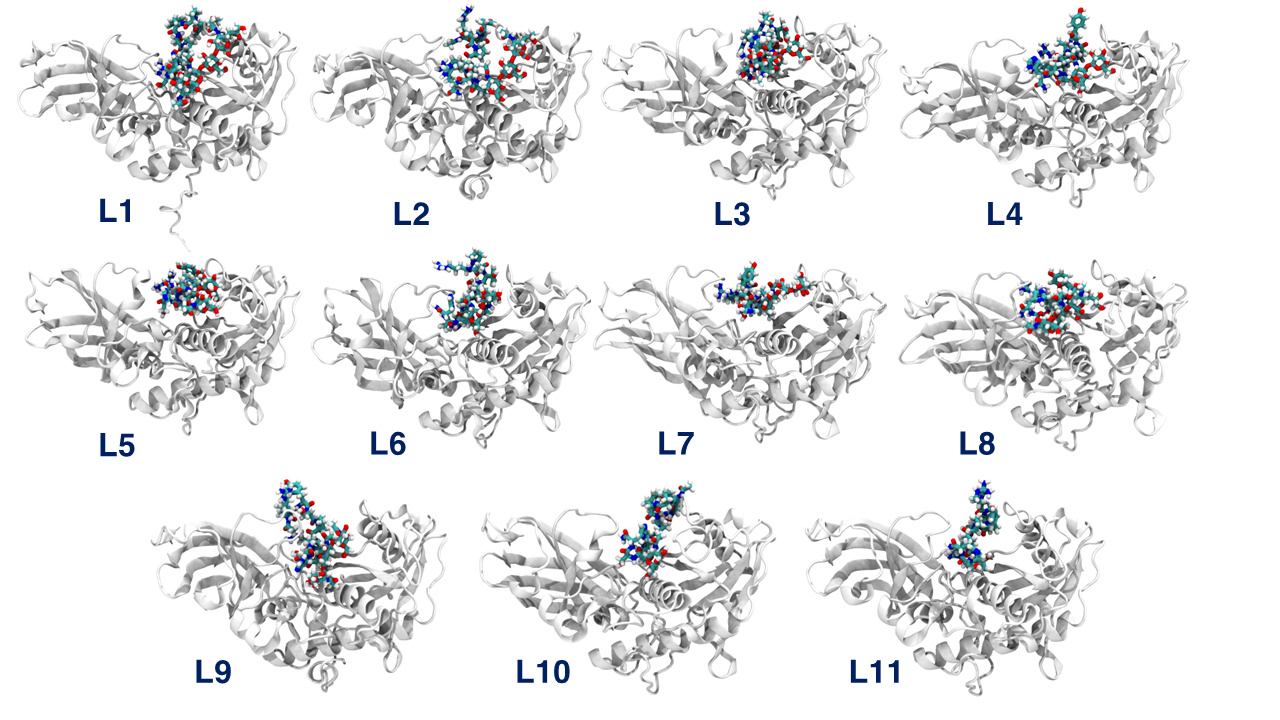


Figure S2. The obtained structures for the OgpA in complexation with different functionalized peptides after 500 ns MD simulations.


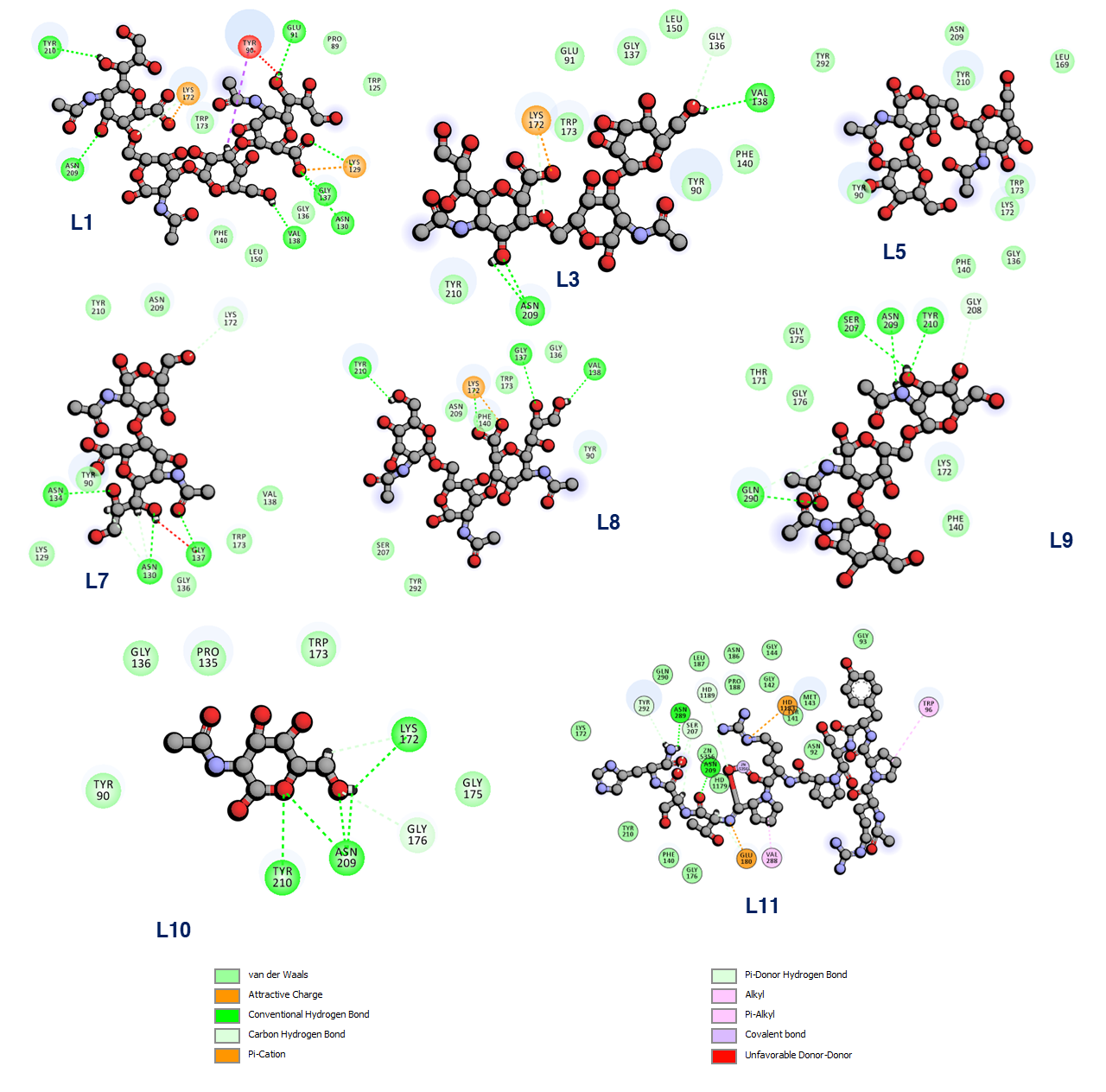


Figure S3. The binding interaction profiles between different functional groups of the peptide and OgpA. The name of each *O*-glycan is presented in Table 1. The L11 structure is the peptide without an *O*-glycan functional group.


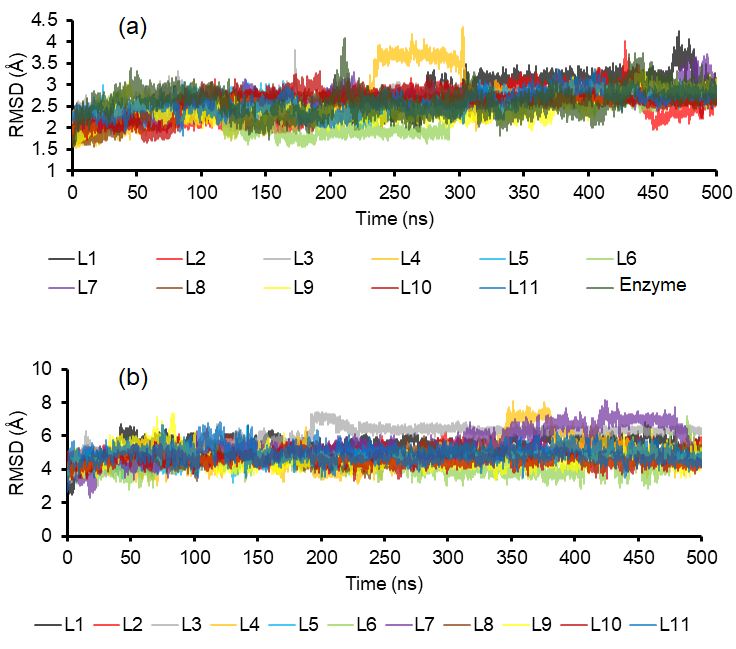


Figure S4. The calculated RMSD values of the enzyme in the presence and absence of the peptides (a) and RMSD values of the functionalized peptides in complexation with the enzyme (b).


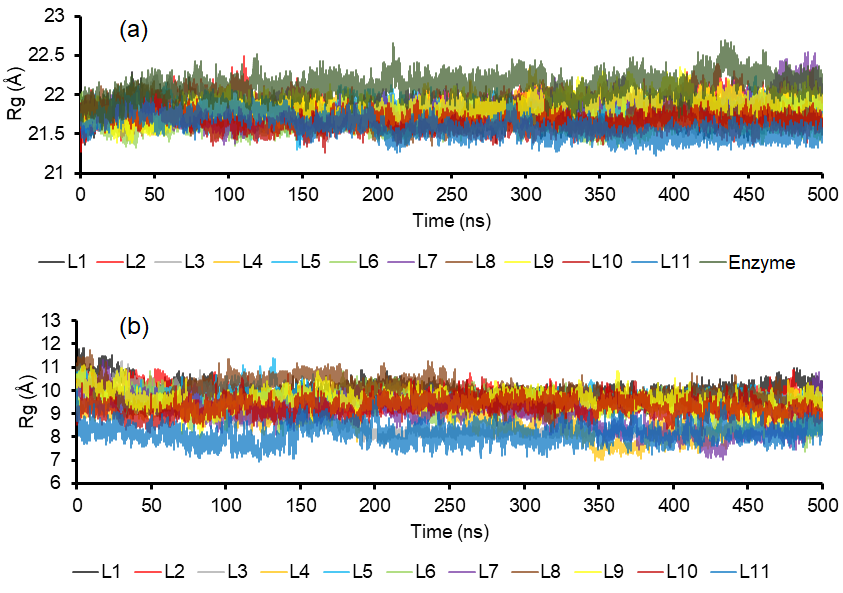


Figure S5. The calculated Rg values of the enzyme in the presence and absence of the peptides (a) and Rg values of the functionalized peptides in complexation with the enzyme (b).


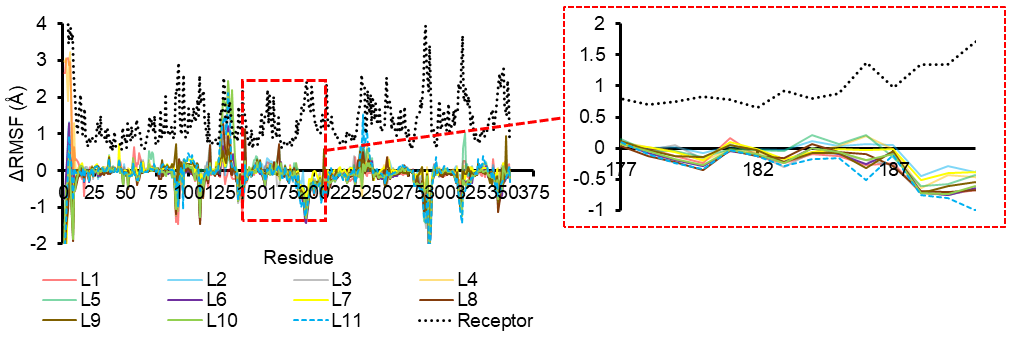


Figure S6. The calculated RMSF of the OgpA in complexation with the functionalized peptide relative to the RMSF values of the free enzyme. The involved residues in the active site show considerable stability in complexation with the peptide.


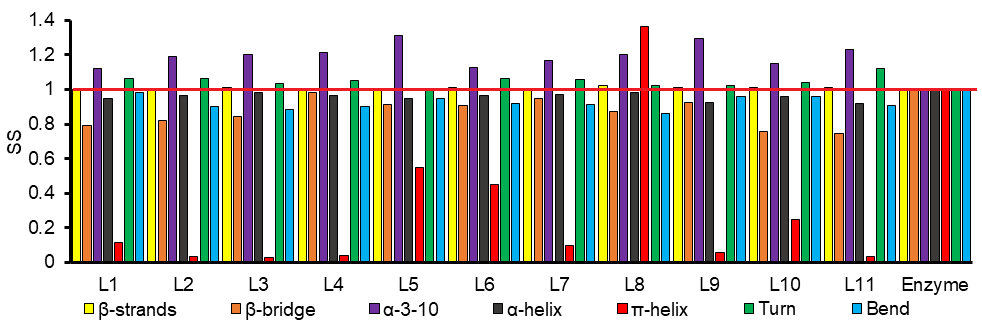


Figure S7. The calculated percentage of the SS of the enzyme in the presence and absence of the functionalized peptides. The corresponding values for the enzyme in complexation with the peptides were calculated relative to the SS of the free enzyme.


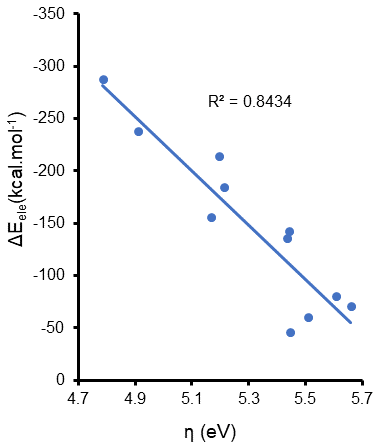


Figure S8. The linear correlation between electrostatic interactions obtained from the MM-GBSA method and the calculated chemical hardness using quantum chemistry calculations.

**XYZ coordinates of the optimized functionalized peptides at B3LYP-D3BJ/Def2-SVP**

L1

C 36.07522800 106.21939600 -2.58802500

O 36.98421900 106.25519900 -3.41768600

C 35.30527800 107.44468600 -2.15915800

H 35.84971100 107.94867100 -1.34302800

H 34.28885800 107.21890500 -1.80671200

H 35.25609400 108.14423400 -3.00543300

N 35.72554100 105.05971800 -1.97079400

C 36.42767900 103.82091600 -2.21559000

C 35.55882600 102.66308400 -1.73142000

O 34.75144500 102.83738400 -0.81256600

C 37.78834800 103.74107000 -1.47278400

C 37.67829700 104.02527700 0.02641500

C 38.93802300 103.60530700 0.77506000

N 38.82628900 103.93985900 2.19326200

C 39.51804600 103.37848100 3.18661500

N 40.35405500 102.36513400 2.94304000

N 39.38127400 103.83750700 4.43811000

H 34.97958800 105.02639900 -1.28252500

H 36.62766500 103.74493600 -3.29322600

H 38.20715800 102.73401600 -1.62555000

H 38.47400200 104.45749600 -1.94911700

H 37.48941300 105.09948500 0.18799300

H 36.82189800 103.48936700 0.45817100

H 39.06018200 102.51561500 0.66913200

H 39.83237700 104.08533300 0.34123800

H 38.20310100 104.70255700 2.43719300

H 40.54151400 102.05078500 2.00045800

H 40.88867200 101.93983300 3.69005400

H 38.64931100 104.50597000 4.64845100

H 39.74424100 103.30966000 5.22947800

N 35.80592600 101.44546900 -2.25287400

C 35.30709900 100.24778900 -1.57257000

C 36.18347500 100.00726400 -0.33315100

O 37.40971800 100.10724400 -0.40080500

C 35.48814800 99.14784800 -2.63011800

C 36.73576600 99.59554000 -3.40334900

C 36.63072500 101.12655600 -3.42955500

H 34.26410900 100.37806700 -1.26926200

H 35.58706700 98.14931600 -2.18178000

H 34.60263100 99.13781400 -3.28454000

H 37.63840900 99.30021700 -2.85225000

H 36.78909000 99.17022400 -4.41505500

H 37.61946200 101.60196500 -3.37520500

H 36.12851300 101.48695300 -4.34254200

N 35.52606500 99.70514100 0.80492600

C 36.22276500 99.48326200 2.05852000

C 35.19339600 99.09088900 3.12193700

O 33.98823900 99.11191400 2.89764700

C 37.05323900 100.71170100 2.51673200

C 36.26720700 101.88055400 3.05716700

C 35.25878700 102.52599400 2.32534900

C 36.55508200 102.37068600 4.33523800

C 34.54963700 103.60360000 2.86549000

C 35.86784200 103.44858600 4.88518800

C 34.83774400 104.04984400 4.16126400

O 34.11589100 105.02704300 4.79249900

H 34.51028800 99.59705200 0.79102300

H 36.93249100 98.64700800 1.93299500

H 37.77291000 100.37985500 3.27957100

H 37.65585200 101.00365600 1.64681700

H 35.01417800 102.20696300 1.31332700

H 37.32653700 101.87968100 4.92205500

H 33.77049300 104.08519000 2.27095500

H 36.08960100 103.80296100 5.88947500

H 33.39026100 105.33339500 4.22925200

N 35.69646500 98.74669000 4.33113600

C 34.81716300 98.53969900 5.47260400

C 34.33936400 99.91770200 5.97049500

O 35.17160600 100.76857600 6.30072400

C 35.51275000 97.71629000 6.54782000

O 36.71102300 98.35793500 6.96563200

H 36.69037800 98.83225200 4.50659000

H 33.96321800 97.95155700 5.11878000

H 34.82147100 97.59090200 7.39817300

H 35.73165000 96.71821800 6.13029700

H 37.11799800 97.82490400 7.66365200

N 33.02377600 100.18648200 5.94803800

C 32.53473800 101.56998000 6.06284600

C 32.89220300 102.21726900 7.40175300

O 32.70591200 101.64948300 8.47241700

C 31.00598200 101.42466800 5.89167600

C 30.72370900 99.97869700 6.30456800

C 31.92805300 99.22607800 5.74305000

H 32.96720200 102.16485400 5.24569800

H 30.44496100 102.16891300 6.46960600

H 30.76915300 101.58374300 4.83329000

H 30.71431700 99.88960100 7.40135600

H 29.77185600 99.59476900 5.91140500

H 32.12460100 98.28603000 6.27457800

H 31.81199500 99.00851700 4.66810400

N 33.36349300 103.48418700 7.29318800

C 33.77172300 104.28459500 8.43339900

C 33.33710100 105.74949200 8.24179500

O 34.11910200 106.67105900 8.47783000

C 35.29823700 104.23483000 8.66256300

C 35.86425500 102.81657600 8.62265500

C 37.34979000 102.75232200 8.25403600

N 37.61727600 101.49377300 7.55193900

C 38.78285000 101.16400400 7.00955900

N 39.85271300 101.98722400 7.11608700

N 38.87830100 100.04462400 6.28493600

H 33.52052300 103.88361500 6.37012800

H 33.25005400 103.86554500 9.30965100

H 35.76628400 104.84480500 7.87353900

H 35.53003700 104.74866100 9.60651900

H 35.67928600 102.28475700 9.56863900

H 35.33082900 102.26285700 7.84400200

H 37.60685500 103.59756300 7.59159900

H 37.99649900 102.83033000 9.14368500

H 36.79686600 100.95637700 7.23518300

H 39.82746400 102.72999000 7.80695400

H 40.77595800 101.59700600 6.95128500

H 38.12042100 99.35547700 6.34519400

H 39.77076100 99.75365400 5.90502700

N 32.09541100 106.00033600 7.76601800

C 31.74973900 107.38204800 7.44940200

C 31.63992600 108.25827200 8.70076700

O 31.11743800 107.86941400 9.73688000

C 30.36424500 107.25775200 6.77972700

C 29.76081100 106.02765000 7.46868500

C 30.95927800 105.08335800 7.57325600

H 32.50861800 107.80906800 6.77633400

H 29.77276200 108.17518200 6.90169400

H 30.48885800 107.06838300 5.70242900

H 29.39784900 106.29731000 8.47101600

H 28.94928200 105.56618400 6.89208500

H 30.88475700 104.36732900 8.40416900

H 31.06458700 104.51754400 6.63707300

N 32.08240300 109.53225100 8.52619400

C 31.59205800 110.62632800 9.32614300

C 30.16204200 110.99188900 8.87254700

O 29.71511500 110.56798300 7.81151000

C 32.52153200 111.85148600 9.13279700

O 32.62511800 112.04208700 7.71640400

C 33.90295200 111.64565300 9.73077800

H 32.39672500 109.79757800 7.59861700

H 31.57872400 110.33522400 10.38631000

H 32.04506800 112.73706100 9.58036600

H 34.52398200 112.53147400 9.53456500

H 34.38750100 110.76424000 9.28621200

H 33.83451900 111.49833400 10.81897200

N 29.48386800 111.87082100 9.65822400

C 28.57021100 112.82183600 9.04005400

C 29.38680700 114.05178200 8.65905800

O 30.27530200 114.45941300 9.40911800

C 27.43494100 113.30143800 9.96825900

O 26.62172000 114.24309500 9.31524700

H 29.98965700 112.24652500 10.45679800

H 28.12913800 112.34707900 8.15587900

H 26.81373800 112.43463300 10.24239300

H 27.88312600 113.70358200 10.89757300

H 27.09495800 115.09934100 9.22364700

N 29.06911000 114.67201800 7.50794300

C 29.56123200 116.01429100 7.27978500

C 29.04709400 116.92473700 8.40975300

O 27.96776700 116.71819500 8.97200100

C 29.17403600 116.51837600 5.87175900

C 27.75452200 116.24930600 5.48943500

N 27.40595900 115.17256000 4.69726700

C 26.55394400 116.86067500 5.80306600

C 26.05173400 115.18172200 4.56277100

N 25.50300400 116.18902100 5.22022200

H 28.21915000 114.39201500 7.02710300

H 30.65722500 116.01378800 7.33852300

H 29.38483600 117.59540500 5.81791700

H 29.83484500 116.01621300 5.15069000

H 28.09082600 114.50797600 4.30033600

H 26.40826400 117.74547900 6.42178000

H 25.52101700 114.43347800 3.97391300

N 29.85875000 117.95112700 8.70496100

H 30.82558500 117.95269400 8.36882300

H 29.59882200 118.57326300 9.46303100

O 33.43036200 114.19306900 7.23365800

C 32.34846300 113.30806100 7.16083200

C 31.93596500 113.05100100 5.70897800

N 30.75109900 112.23646700 5.64369100

C 33.14306900 112.42907100 5.00573300

O 32.80371900 112.09647600 3.66843100

C 34.30319500 113.42900100 5.03211900

O 33.99479300 114.52618700 4.19756400

C 34.59643600 113.86147600 6.47737500

C 35.57128600 115.05659000 6.62283600

C 29.68434500 112.54037800 4.87764500

O 29.62470100 113.57674500 4.20016300

C 28.56994500 111.52219400 4.88487200

O 34.95215200 116.27786100 6.95461700

H 31.55478700 113.80352200 7.72488500

H 31.70308400 114.01004600 5.23137500

H 30.70571900 111.39460700 6.21351800

H 33.43710900 111.51996900 5.54942700

H 35.21643000 112.92010300 4.66814700

H 33.68645100 114.15235000 3.35649200

H 35.07469100 112.98160000 6.94201500

H 36.24744100 114.85277700 7.46466100

H 36.19197200 115.15566500 5.71814500

H 27.61525500 112.01292600 4.65620000

H 28.51125900 110.99965700 5.84837200

H 28.79012000 110.77783800 4.10372400

C 33.28608100 110.88010300 3.19771700

C 32.14762500 109.99042600 2.69612300

C 32.74624300 108.68728900 2.17714700

C 33.84657900 108.92774400 1.12781100

C 34.84473700 109.95033100 1.68633600

C 35.88865400 110.39407600 0.66721400

O 31.15705600 109.74344600 3.67973800

O 31.67588600 107.93875800 1.65244100

O 33.29381400 109.36911600 -0.07842600

O 34.18921800 111.13150900 2.14821600

O 36.71195800 109.32580600 0.24732900

H 33.81957100 110.34984300 4.01542900

H 31.64674000 110.51043400 1.86731200

H 33.19056400 108.14860300 3.03713200

H 34.40657600 107.98322900 0.98575300

H 35.36552100 109.47479500 2.54364800

H 35.36753600 110.76939700 -0.22408500

H 36.47360400 111.22264000 1.10595300

H 31.57300500 109.35562500 4.46413900

H 32.82103100 108.58429300 -0.47363200

H 37.22233400 109.00970400 1.00623800

C 32.22411100 105.74598500 0.68014400

C 31.60838500 106.20887700 -0.66912600

O 32.14815000 107.23410700 -1.17965800

O 30.64544100 105.57001300 -1.12710800

C 31.67531000 106.55198700 1.86592300

C 30.23831600 106.16901300 2.19587300

C 30.16611400 104.67225300 2.48483200

C 30.76955300 103.87754300 1.31094600

C 30.94485400 102.39997200 1.65872300

C 31.58743300 101.59825200 0.51719700

C 31.74613200 100.13272400 0.89175800

O 32.48338400 99.42644100 -0.09582400

O 30.87824800 101.72085900 -0.71101100

O 31.69135300 102.21775400 2.85132400

O 32.06851300 104.36469300 0.98280800

N 28.81355700 104.24075900 2.76696400

C 28.36096500 103.95865400 4.01775500

C 26.87904600 103.65962500 4.11820900

O 29.08681300 103.95338700 5.00916400

O 29.79158500 106.90340300 3.31631100

H 33.31113200 105.90067200 0.61899800

H 32.29055700 106.31973400 2.75666800

H 29.61259200 106.39237300 1.30964100

H 30.75185100 104.46145500 3.38864600

H 30.12349500 103.96909800 0.42582900

H 29.94240500 101.99115700 1.86921100

H 32.58209700 102.02430700 0.31177600

H 32.29581700 100.03479100 1.83235200

H 30.74107500 99.68234200 1.02972000

H 32.10637200 99.67600400 -0.95422000

H 29.97696300 101.38992800 -0.57575800

H 32.58870400 102.56028200 2.71293900

H 28.13830900 104.28544900 2.01144300

H 26.73291000 102.81672000 4.80767000

H 26.41515000 103.42916100 3.14911100

H 26.37221500 104.53670700 4.55162600

H 30.09775500 107.82171900 3.19469800

C 34.96476400 117.57128400 4.84655200

C 36.19999700 118.34317200 5.38617700

O 36.00458000 119.51181300 5.80672300

O 37.27087500 117.69595300 5.38808800

C 34.15154300 116.92832600 5.97996300

C 33.35988600 117.96189900 6.76505700

C 32.47462800 118.79357100 5.84564200

C 33.28959700 119.35652400 4.66586300

C 32.38491000 119.87239100 3.54251600

C 33.16402300 120.77119800 2.57457500

C 32.34936600 121.17479000 1.34939700

O 33.07880700 122.07511400 0.54351700

O 33.64337300 121.94519100 3.22960900

O 31.79360700 118.77967900 2.86306500

O 34.06742700 118.34136000 4.04007600

N 31.84468200 119.86279600 6.60250300

C 30.54835300 120.24279600 6.46951500

C 30.09033900 121.35056900 7.39453700

O 29.77846000 119.71848500 5.66662100

O 32.54883500 117.30543600 7.73487100

H 35.31638900 116.77773100 4.17706900

H 33.44381300 116.21946900 5.52530100

H 34.08230000 118.63164700 7.26533800

H 31.67513300 118.15827700 5.44072600

H 33.94597800 120.15649500 5.03258200

H 31.56309700 120.46336300 3.97844000

H 34.06810900 120.23709400 2.24034300

H 32.10421100 120.28546700 0.75155300

H 31.38759000 121.61776100 1.68946800

H 33.50776500 122.68263500 1.16811900

H 32.89124900 122.37784300 3.66377900

H 32.49951000 118.11000200 2.82616000

H 32.43052700 120.37314100 7.25418900

H 29.36078000 120.92580000 8.10093000

H 29.56858800 122.11520200 6.80156200

H 30.90656300 121.81817800 7.96154500

H 33.03581100 116.50758700 8.00808300

L2

C 44.02276200 94.43349000 2.07889800

O 44.52403600 93.95475800 3.09415100

C 44.81305800 94.61567600 0.79892000

H 45.62328700 95.33752100 0.98512000

H 44.20381000 94.97064900 -0.04316600

H 45.28310800 93.65755300 0.53263200

N 42.73117400 94.84168700 2.03508700

C 41.85042000 94.74600600 3.17770100

C 40.41427800 94.81743400 2.65836300

O 40.17527200 95.36828400 1.57983400

C 42.08741000 95.88821300 4.19691100

C 41.78291400 97.27802500 3.64191100

C 41.96296900 98.36006100 4.70063100

N 41.49421600 99.64807800 4.18832900

C 41.02782800 100.65034800 4.93021600

N 41.11657100 100.61967800 6.25860200

N 40.39229800 101.68137500 4.33479800

H 42.04377200 93.79066500 3.68658100

H 41.46629300 95.71008900 5.08819200

H 43.13659000 95.81644900 4.52186000

H 42.43472400 97.48847800 2.77788800

H 40.74312900 97.31854200 3.28684800

H 41.36104000 98.10459800 5.58683900

H 43.01719100 98.43265600 5.01725600

H 41.50833800 99.77968600 3.18135000

H 41.64821300 99.90397600 6.73747900

H 40.66598100 101.32669100 6.82750400

H 40.39765600 101.75155800 3.32263500

H 40.29610300 102.56048300 4.83351300

H 42.31944400 95.22552100 1.18948400

N 39.42504300 94.37121500 3.45847100

C 38.03996100 94.70370200 3.11903200

C 37.88948800 96.22768400 3.09982700

O 38.40099200 96.91993400 3.99600400

C 37.21744900 94.04596000 4.24689700

C 38.20178800 93.96119100 5.41952400

C 39.53344700 93.65041600 4.73570900

H 37.77640000 94.30374300 2.12964800

H 36.30400400 94.61141500 4.47805700

H 36.91379400 93.03931300 3.92304100

H 38.27038600 94.93195300 5.93208700

H 37.92347600 93.19773700 6.15869200

H 40.39523600 93.98842900 5.32223000

H 39.64908900 92.56927600 4.54827800

N 37.17152400 96.72603500 2.08958900

C 37.01918100 98.13694100 1.81267900

C 35.53148200 98.48013700 1.70785100

O 34.69919300 97.66471000 1.32889900

C 37.80757000 98.54148400 0.53811300

C 37.43933900 97.72736300 -0.67614400

C 38.06607100 96.49615300 -0.92715900

C 36.42484800 98.14492900 -1.55230300

C 37.69237500 95.70659200 -2.01740700

C 36.04656800 97.36872800 -2.64594000

C 36.67913800 96.13907700 -2.88480000

O 36.27683700 95.41777500 -3.96154300

H 36.78438800 96.08864000 1.40035500

H 37.44219300 98.68586700 2.65789300

H 37.63608300 99.61310700 0.35643800

H 38.87543500 98.41546500 0.77166500

H 38.85347900 96.14373400 -0.25571700

H 35.92041100 99.09884900 -1.37657200

H 38.19144500 94.75059300 -2.20015200

H 35.26075600 97.69962300 -3.32804200

H 36.78813800 94.59763700 -4.01614400

N 35.24194100 99.75319600 2.05476500

C 33.91579100 100.33249400 1.98518800

C 34.11300600 101.85018200 2.08349800

O 35.17401400 102.29914000 2.52974700

C 33.04271500 99.83729500 3.15985600

O 33.48913700 100.32545200 4.40383000

H 35.97173100 100.41371900 2.31779300

H 33.43040900 100.04392900 1.03976200

H 32.00761900 100.18389700 3.03581300

H 33.03437000 98.73376500 3.12137600

H 34.43253000 100.12569700 4.49943500

N 33.08285200 102.64920400 1.75514700

C 33.18499500 104.09213200 2.01773700

C 33.25327000 104.32683800 3.53245500

O 32.32963500 104.00506300 4.27318000

C 31.89397100 104.67120100 1.40661500

C 30.92121500 103.48540800 1.39431200

C 31.82221600 102.29152300 1.08650100

H 34.08791900 104.49459700 1.53646100

H 31.50962400 105.52710600 1.97749900

H 32.10061400 105.01373500 0.38134700

H 30.48604900 103.36223000 2.39668000

H 30.10854500 103.60969700 0.66717500

H 31.41368700 101.34755400 1.46564800

H 31.99283500 102.18014300 0.00153500

N 34.37764500 104.94326100 3.96102900

C 34.67577000 105.18695400 5.36071700

C 34.99207300 106.67230700 5.61577700

O 36.00016100 107.01526600 6.23058200

C 35.80845400 104.27292900 5.83611200

C 35.39055900 102.80032400 5.89378300

C 36.59304500 101.87449100 5.75473600

N 36.20493500 100.47759100 5.95820100

C 36.94318200 99.42344300 5.57067100

N 38.04919500 99.58603100 4.84388600

N 36.56507300 98.18294600 5.88780800

H 35.13763500 105.04928800 3.29787000

H 33.75100200 104.94829000 5.91013000

H 36.66144300 104.40441600 5.14799700

H 36.15858800 104.61827000 6.81824800

H 34.87234800 102.60264800 6.84775900

H 34.68341900 102.55692800 5.08949700

H 36.98725000 101.98097300 4.73307900

H 37.39409000 102.15304900 6.46212800

H 35.51645900 100.30370600 6.68555700

H 38.40273900 100.50412200 4.59971600

H 38.47501900 98.74399400 4.44125400

H 35.67675800 97.99860900 6.33684600

H 37.09904000 97.40595600 5.50170900

N 34.10534400 107.58334800 5.14642400

C 34.30625500 108.98835000 5.46715400

C 34.04145400 109.24728100 6.95824800

O 33.46796400 108.44881400 7.68341700

C 33.24878500 109.70570200 4.60359300

C 32.10144000 108.69409800 4.56055700

C 32.81948600 107.34771000 4.47024300

H 35.33346700 109.30160000 5.23033400

H 32.94692800 110.66286200 5.04676500

H 33.66250700 109.87478900 3.59647200

H 31.50531000 108.76388500 5.48163600

H 31.42601500 108.82578600 3.70940200

H 32.25728400 106.52811300 4.93739400

H 32.99151100 107.07764100 3.41740400

N 34.44912700 110.47301000 7.39039300

C 33.87533800 111.03574700 8.58896200

C 32.35114200 111.19582700 8.41920700

O 31.81995400 111.18041700 7.31226300

C 34.48387800 112.42996000 8.85418500

O 34.16152500 113.19575900 7.68978800

C 35.98484200 112.39597700 9.10209600

H 34.72315200 111.15088900 6.68530600

H 34.07627800 110.37702500 9.44679600

H 33.96932100 112.87370200 9.72168600

H 36.37337900 113.41628000 9.22298100

H 36.51015100 111.92338400 8.25960000

H 36.20628300 111.82254200 10.01459500

N 31.67302500 111.43380800 9.56523400

C 30.38516700 112.08967600 9.57816300

C 30.58311300 113.58171100 9.86362500

O 31.58748900 113.98979400 10.44760200

C 29.46573400 111.46770600 10.66177700

O 28.23328900 112.13619300 10.77761600

H 32.21502100 111.52791100 10.41929200

H 29.92076800 111.94894700 8.59465800

H 29.27136200 110.42449300 10.36987200

H 30.01418000 111.44744400 11.62099500

H 28.33938700 112.89015100 11.38559200

N 29.55057200 114.38121900 9.51648900

C 29.40962000 115.73248000 10.04022000

C 28.60197800 115.70534300 11.35743100

O 27.65763200 116.44279300 11.56903000

C 28.81521800 116.69449400 9.00574700

C 27.59662300 116.17533600 8.30482400

N 27.63836600 115.80243800 6.97836200

C 26.29333200 115.92671200 8.70279900

C 26.40111900 115.35392900 6.63571900

N 25.55978800 115.41806900 7.65400100

H 28.69815400 113.93205800 9.19003900

H 30.42429600 116.06859600 10.30304400

H 28.59082300 117.63828200 9.52180400

H 29.58987400 116.89666800 8.25381900

H 28.50680800 115.79734800 6.41517700

H 25.86694700 116.09556900 9.68972100

H 26.17098700 114.99089600 5.63389100

N 29.01859300 114.74871200 12.25279100

H 29.99495500 114.45935900 12.22341400

H 28.61801000 114.81343800 13.18553900

O 34.84926100 115.40072300 8.02166600

C 33.76228400 114.53657700 7.84430600

C 32.97248800 114.88899100 6.57269200

N 31.85086300 113.99167300 6.43358100

C 33.92907700 114.84071400 5.37428700

O 33.23775000 115.28653200 4.21556700

C 35.13932100 115.75446100 5.61920500

O 34.72421400 117.10474000 5.62360200

C 35.79868000 115.40064300 6.94735900

C 36.89904200 116.35222000 7.38886000

O 37.41515600 115.96968800 8.64857500

C 30.58614800 114.37400500 6.17498700

O 30.25023900 115.56349800 6.07041200

C 29.57872000 113.25341900 6.04035200

H 33.13415100 114.64789400 8.74135900

H 32.58516000 115.91014600 6.67212300

H 32.04474100 112.99239300 6.48335800

H 34.27178300 113.80417900 5.24160000

H 35.88124500 115.57245000 4.81621400

H 34.15746000 117.21138300 4.84270100

H 36.22198500 114.38687700 6.84951700

H 37.72400300 116.32099200 6.66095900

H 36.49526300 117.38052300 7.40505000

H 36.64791600 115.89808200 9.23706000

H 28.79184200 113.39269500 6.79467000

H 30.03082900 112.26299900 6.16546500

H 29.09867300 113.31375200 5.05420800

C 33.55123100 114.65413600 3.00958900

C 32.38019800 113.75692600 2.59675000

C 32.57272100 113.16530100 1.19586400

C 32.95412300 114.28152100 0.21170400

C 34.08208800 115.14841500 0.76925200

C 34.39328800 116.35657300 -0.11236700

O 32.16008300 112.77100100 3.59969600

O 31.39269000 112.60243900 0.64997600

O 31.81879400 115.08359800 -0.03809200

O 33.76491500 115.66041100 2.05957700

O 34.74934100 115.98312300 -1.42456800

H 34.47138700 114.04154800 3.12248000

H 31.48139600 114.38584500 2.58428600

H 33.38240200 112.40972700 1.22561400

H 33.32105700 113.81509200 -0.72130700

H 34.98736100 114.51070900 0.84016400

H 33.48495600 116.97178200 -0.18274300

H 35.17595000 116.95750400 0.38568200

H 32.85784200 112.10031700 3.57045600

H 31.09070200 114.45269200 -0.16800200

H 35.60647600 115.53558700 -1.39235200

C 30.71826900 110.27554000 0.17582800

C 29.74850700 110.53153200 -1.02514700

O 29.17087900 109.50558100 -1.48662300

O 29.65303300 111.69566600 -1.44674900

C 30.87943800 111.40762200 1.20721200

H 31.59639300 111.02043500 1.94690900

C 29.54343900 111.64224600 1.93313300

H 28.79060900 111.92091000 1.17649900

C 29.13691900 110.31359700 2.59523400

H 29.91766900 110.04182200 3.31959400

C 29.07923000 109.21805000 1.51978500

H 28.33255500 109.52195800 0.76729200

C 28.66515500 107.82787400 1.99077300

H 27.60218800 107.89774500 2.29488400

C 28.76543700 106.84051900 0.81885400

H 29.84095600 106.64938300 0.64956300

C 28.07299800 105.51949300 1.12511500

H 28.55807000 105.02005300 1.97499500

H 27.02077500 105.72977700 1.40826200

O 28.13082900 104.66591500 -0.00156600

H 28.01251600 105.26771700 -0.75721900

O 28.15410300 107.32937900 -0.35577400

H 28.66663500 108.09771300 -0.73722400

O 29.45631700 107.33044700 3.05166500

H 29.21800700 107.85423400 3.84822500

O 30.36001900 109.11277100 0.91045200

N 27.90894300 110.47926300 3.34996400

H 27.27879200 111.22437000 3.07497400

C 27.71320900 109.88464200 4.54768900

C 26.53672200 110.37754000 5.35655200

H 26.91266000 111.07001000 6.12744200

H 26.06763700 109.52691500 5.86859400

H 25.79025100 110.90624700 4.74836400

O 28.47016600 109.00598000 4.98090100

O 29.58337100 112.69269300 2.86558300

H 30.39493600 112.57573700 3.39647100

H 31.71025200 110.06944500 -0.25895800

L3

C 44.19723200 101.35575300 -0.08792600

O 45.21167100 100.89964400 -0.61194000

C 44.02081100 102.83866600 0.17110200

H 43.01665600 103.10174900 0.53050000

H 44.23419900 103.38913400 -0.75676500

H 44.76348200 103.15676900 0.91932800

N 43.16971500 100.56585400 0.30591500

C 43.19143200 99.12899700 0.15163700

C 41.74941100 98.63185800 0.24459600

O 40.90172800 99.30969600 0.83310800

C 44.02765800 98.42825200 1.25199100

C 43.51040600 98.66544500 2.67285000

C 44.11098500 97.66021600 3.65289800

N 43.58066500 97.78182800 5.00853500

C 42.79885200 96.89256900 5.63157800

N 42.20201000 95.90666000 4.95592400

N 42.62755600 96.99240800 6.95676900

H 42.33242700 100.94955900 0.73470400

H 43.63771200 98.88912900 -0.82428400

H 44.03829100 97.34690800 1.04774800

H 45.06560800 98.77935100 1.14974200

H 43.74026000 99.69308500 2.99732200

H 42.41719500 98.56634900 2.69917000

H 43.91976700 96.63648500 3.30483300

H 45.20488100 97.77940100 3.70638100

H 43.89831000 98.57479600 5.55655100

H 41.93919400 96.01182700 3.96157300

H 41.69942900 95.20006400 5.48031000

H 43.21662900 97.59221000 7.52152700

H 41.94391700 96.41889700 7.43505800

N 41.47085700 97.38935400 -0.20017700

C 40.22711000 96.75312900 0.24304100

C 40.26260200 96.64815500 1.77266400

O 41.28924600 96.26135400 2.34712400

C 40.26027300 95.37262200 -0.44188500

C 41.75568100 95.09911800 -0.64221100

C 42.32170100 96.47926800 -0.98092400

H 39.35456100 97.34708500 -0.06235100

H 39.74874400 94.60305700 0.15254900

H 39.74543300 95.44524500 -1.41186500

H 42.20052700 94.73975700 0.29684000

H 41.95616900 94.36099600 -1.43085200

H 43.37970600 96.57637300 -0.71095700

H 42.22196800 96.70458900 -2.05643300

N 39.14075500 96.99518000 2.41105400

C 39.00618400 97.03309000 3.85188100

C 37.50753500 97.17668000 4.13977300

O 36.71567400 97.44633300 3.24083600

C 39.83244200 98.17540000 4.50883500

C 39.23141800 99.56004000 4.44586100

C 38.79506400 100.14036400 3.24418900

C 39.09462800 100.30568100 5.62493500

C 38.18173600 101.39560200 3.23452300

C 38.51149800 101.56986600 5.62526000

C 38.02338800 102.10471000 4.43166400

O 37.35392400 103.29821100 4.49960800

H 38.31489200 97.29620200 1.89640300

H 39.36326200 96.07914200 4.27023500

H 40.00603400 97.91269300 5.56199200

H 40.81876100 98.16236500 4.02599700

H 38.94821500 99.61882200 2.29982400

H 39.43898400 99.87473500 6.56622800

H 37.81982600 101.82284500 2.29524000

H 38.39268000 102.13722100 6.54760800

H 37.12887900 103.60822800 3.60947900

N 37.12916000 97.02337900 5.42488700

C 35.76553300 97.32223600 5.83719400

C 35.57140400 98.84744000 5.79747100

O 36.27746300 99.57612600 6.50225900

C 35.47902700 96.71198100 7.20315400

O 36.46105500 97.12322000 8.14341400

H 37.82401300 96.88369400 6.14979300

H 35.08957400 96.83951100 5.12212900

H 34.46919900 97.02238000 7.52195700

H 35.48345900 95.61350400 7.09528700

H 36.28755600 96.67335100 8.98262100

N 34.68365300 99.35818900 4.92912500

C 34.67261800 100.80117600 4.66412000

C 34.16399300 101.58773700 5.87583100

O 33.22780500 101.18883700 6.56032100

C 33.73751800 100.92966300 3.44502100

C 32.79217300 99.73355200 3.59127600

C 33.71381400 98.62414500 4.09961700

H 35.69303400 101.12526600 4.42823300

H 33.21924500 101.89781900 3.41684100

H 34.33637500 100.84157300 2.52551300

H 32.02631000 99.95492700 4.34909100

H 32.28972300 99.46007600 2.65331200

H 33.17571100 97.87412700 4.69452100

H 34.24222200 98.11538400 3.27778500

N 34.81045100 102.76093900 6.08983000

C 34.60818900 103.55298000 7.29378500

C 34.34767700 105.02558300 6.94906700

O 35.04627900 105.92769800 7.40893700

C 35.79272600 103.39964800 8.26039400

C 36.01220100 101.93046600 8.61065500

C 37.38659000 101.59024300 9.18519700

N 37.68330100 100.19157200 8.87965600

C 38.77402000 99.54277500 9.27982200

N 39.66354800 100.12748200 10.08915900

N 38.98097300 98.29195800 8.83727600

H 35.63183400 102.98014400 5.52305800

H 33.69939600 103.15029800 7.76730600

H 36.68976800 103.81774500 7.77378300

H 35.61038500 104.01859300 9.15104200

H 35.22065300 101.56839900 9.28627100

H 35.91833000 101.35170800 7.68601800

H 38.15645100 102.24277200 8.73374800

H 37.42388200 101.75175900 10.27518500

H 37.13760300 99.75733800 8.12131800

H 39.57900100 101.10113900 10.34947800

H 40.47309700 99.62231000 10.42631400

H 38.18589900 97.78839800 8.43498600

H 39.75246500 97.74613600 9.20095800

N 33.30755500 105.28982500 6.12294500

C 32.96272100 106.67887800 5.84969500

C 32.49085900 107.38334200 7.12597200

O 31.94783300 106.79117500 8.04885200

C 31.79480900 106.57284200 4.84522900

C 31.13172800 105.24625900 5.23316300

C 32.32570700 104.34947800 5.56590100

H 33.82549200 107.22271800 5.43722600

H 31.12072800 107.43766500 4.91060800

H 32.19864800 106.52319000 3.82223500

H 30.50901100 105.38720000 6.12911900

H 30.50365900 104.82562200 4.43552400

H 32.08355300 103.54768400 6.27582000

H 32.72966300 103.87653100 4.65558500

N 32.65679300 108.73122700 7.11549700

C 31.92717700 109.56001000 8.04044900

C 30.42296900 109.54077900 7.70762600

O 30.02293800 109.21285900 6.59596600

C 32.44482800 111.01471600 7.95650800

O 32.28618300 111.42857100 6.59183000

C 33.89650900 111.15769600 8.38392200

H 32.93512200 109.17677000 6.24650400

H 32.07376300 109.18412900 9.06384900

H 31.80746900 111.64409500 8.59588500

H 34.19614700 112.21300500 8.31312800

H 34.55613900 110.55738600 7.74095000

H 34.02546000 110.82552300 9.42481600

N 29.59905700 110.00302800 8.68854500

C 28.40542600 110.75661800 8.33532700

C 28.80633600 112.22635300 8.25684500

O 29.64362800 112.68344800 9.03592100

C 27.26460300 110.65072100 9.37087000

O 26.17369800 111.45592700 9.00064600

H 30.05805100 110.34892600 9.52815400

H 28.04336300 110.39088600 7.36717600

H 26.92660300 109.60379100 9.41552800

H 27.66584100 110.91843600 10.36746100

H 26.38311000 112.40409300 9.15181300

N 28.18597700 112.98334500 7.33240600

C 28.27392600 114.42450900 7.44304400

C 27.69142900 114.84507700 8.80435200

O 26.77958100 114.21127000 9.34371500

C 27.58234200 115.11687400 6.24754900

C 26.22589500 114.57786800 5.92987700

N 26.03655400 113.57033600 5.00265500

C 24.96540600 114.83323100 6.43741500

C 24.70761700 113.27454300 4.98416600

N 24.02957500 114.01797100 5.84120400

H 27.37105700 112.59647500 6.86495100

H 29.33014500 114.72276300 7.43296600

H 27.52126500 116.19352700 6.45870300

H 28.23515200 114.98520200 5.37221800

H 26.79428700 113.16636600 4.42614400

H 24.70066300 115.56263300 7.20233500

H 24.29136900 112.51291300 4.32466900

N 28.24631100 115.94832200 9.32763600

H 29.13158800 116.30102900 8.95430000

H 27.93486300 116.26326800 10.24035700

O 32.36633500 113.76749000 6.59416700

C 31.58753200 112.63004300 6.36072300

C 31.06573900 112.59278100 4.92480600

N 30.17580100 111.47298500 4.73314600

C 32.24103400 112.62153200 3.95163000

O 31.67596400 112.59476600 2.64550400

C 33.07024700 113.89713600 4.21998900

O 32.35332100 115.03658400 3.80793700

C 33.46218800 113.97210800 5.70304000

C 34.13959100 115.29651800 6.13191000

C 28.91701200 111.61100300 4.26900300

O 28.44578200 112.70767100 3.93630800

C 28.10660900 110.34063800 4.16079300

O 33.30529500 116.17479200 6.85302100

H 30.75794600 112.72191300 7.06502500

H 30.48111400 113.50481800 4.75307700

H 30.46197700 110.56476900 5.09610800

H 32.87798800 111.73122500 4.09647000

H 34.02309000 113.82714300 3.66397300

H 32.31686900 114.98150400 2.83505200

H 34.19162300 113.15735500 5.85293900

H 34.95595200 115.05889900 6.82823700

H 34.58552600 115.79850900 5.25887700

H 28.03034500 110.06426400 3.09722000

H 27.08776400 110.53394500 4.52610400

H 28.54983900 109.51166400 4.72503600

C 32.54340400 112.63797300 1.57604700

C 31.87801600 112.01947700 0.34770900

C 32.78550200 112.18607300 -0.87395200

C 33.15081900 113.65646600 -1.08609900

C 33.71414500 114.23833200 0.20945600

C 33.93889700 115.74794300 0.14880800

O 31.50017100 110.67023600 0.51882700

O 32.12822300 111.73202800 -2.03987600

O 32.00167200 114.37972400 -1.47443000

O 32.82370000 114.00866900 1.29858500

O 34.83556800 116.11239700 -0.87507100

H 33.49604300 112.11412200 1.80849200

H 30.94993800 112.58389400 0.17362900

H 33.71787600 111.61092700 -0.69880000

H 33.94163500 113.72658200 -1.85681800

H 34.67964000 113.73448700 0.41521600

H 32.97519700 116.22854700 -0.07286300

H 34.26936900 116.09041400 1.14692700

H 32.28938600 110.14510500 0.72208800

H 31.53793800 113.80639800 -2.10637800

H 35.71865900 115.80029900 -0.63286000

H 31.62745300 110.93965500 -1.78838700

C 32.72957700 117.93092400 5.20920400

C 33.82445700 118.82465900 5.85246200

O 33.43604600 119.75765700 6.60038300

O 35.00192000 118.48983100 5.59156800

C 32.24975900 116.83229800 6.16929400

C 31.36502500 117.39189200 7.27149800

C 30.18966300 118.16208000 6.68324900

C 30.68212500 119.20693800 5.66399500

C 29.53780500 119.74702200 4.80047900

C 29.94904700 121.03926800 4.08390800

C 28.90357600 121.52591500 3.08519700

O 29.29058300 122.76208700 2.52463100

O 30.23038200 122.08265800 5.01611900

O 29.12682500 118.75774300 3.87443200

O 31.57892800 118.62897100 4.72156000

N 29.42200500 118.78828900 7.74614900

C 28.06573500 118.81184900 7.80417700

C 27.47230400 119.48351600 9.02450300

O 27.35227300 118.30968600 6.93781100

O 30.87554800 116.32512900 8.07853000

H 33.15197900 117.45188100 4.31849400

H 31.66100900 116.11071200 5.58492000

H 31.97514200 118.08101700 7.88281400

H 29.52228200 117.46137300 6.16329200

H 31.18124100 120.02500700 6.20014300

H 28.67002300 119.96873100 5.44276500

H 30.89728500 120.86495200 3.55023900

H 28.78740000 120.79239200 2.27479800

H 27.92265400 121.60154700 3.60388600

H 29.65223600 123.27391700 3.26654700

H 29.46135400 122.18188600 5.59931300

H 29.95611000 118.32740700 3.60004000

H 29.94297200 119.26155800 8.47615900

H 26.97169300 118.71249300 9.62987900

H 26.70264300 120.19711400 8.69741900

H 28.21384200 120.00198100 9.64712300

H 31.56684300 115.63944000 8.07717600

L4

C 44.20004900 95.26614400 7.78275000

O 43.76672700 94.67069900 8.76702400

C 45.64984800 95.69312800 7.67219900

H 45.90030800 96.14060900 6.70082100

H 46.29205600 94.81763300 7.84834800

H 45.86390800 96.42195300 8.46913900

N 43.41049200 95.59187800 6.73067000

C 42.00278900 95.26399700 6.68760900

C 41.56721000 95.31518900 5.22250200

O 42.20688100 95.99088800 4.41087100

C 41.14911500 96.24513700 7.52769200

C 41.18491200 97.68454700 7.01915000

C 40.26326900 98.58661600 7.83181800

N 40.19333800 99.91533600 7.22404500

C 39.20491900 100.78999600 7.40381700

N 38.25151500 100.56513300 8.30585200

N 39.13120000 101.88356600 6.61901800

H 41.86821700 94.25500200 7.10430400

H 40.10638500 95.89313200 7.54444800

H 41.51891700 96.18591000 8.56273500

H 42.21615500 98.07240600 7.05913800

H 40.86044300 97.71701800 5.96893500

H 39.24765600 98.15907100 7.83986300

H 40.60688500 98.65972000 8.87781400

H 40.95748000 100.19034000 6.61452500

H 38.31375800 99.79470700 8.95917200

H 37.44411800 101.17322700 8.37556100

H 39.89765700 102.10032200 5.99073500

H 38.56839000 102.67449100 6.91492000

H 43.77821900 96.07066800 5.91385300

N 40.42392700 94.69230300 4.87281100

C 39.86210400 94.94918600 3.54730300

C 39.59858600 96.45050000 3.39561100

O 39.17668500 97.12017900 4.34760600

C 38.55800000 94.12102300 3.53114200

C 38.19565500 93.97900900 5.01408600

C 39.55834700 93.82923700 5.68996300

H 40.56075300 94.62906900 2.76076300

H 37.77227900 94.59610200 2.92727700

H 38.76788000 93.13635500 3.08807000

H 37.70332600 94.89311900 5.37875300

H 37.53226500 93.12658400 5.21361200

H 39.54478900 94.14276500 6.73951200

H 39.91740500 92.78682900 5.64734900

N 39.79915700 96.94968100 2.16480900

C 39.89344800 98.36921100 1.86462200

C 38.94501000 98.79694100 0.73194500

O 39.29354600 99.56215400 -0.15697000

C 41.35836000 98.77949000 1.61025300

C 42.08968200 97.87114000 0.64812200

C 42.07244400 98.09628300 -0.73678700

C 42.77395300 96.73774000 1.12414800

C 42.71945200 97.22948600 -1.61918100

C 43.41871700 95.86105200 0.25095400

C 43.39638000 96.10247100 -1.13028200

O 44.03920800 95.22381500 -1.94101900

H 40.21224200 96.32765700 1.47512300

H 39.52968600 98.90327300 2.75504700

H 41.35270800 99.81455500 1.24447400

H 41.86862800 98.76756800 2.58436100

H 41.53029600 98.95875500 -1.12731000

H 42.79258700 96.53208000 2.19766700

H 42.69957700 97.42452200 -2.69527200

H 43.94799200 94.98237300 0.62567500

H 43.94708500 95.50139500 -2.86384700

N 37.67245900 98.32802200 0.82941500

C 36.63061400 99.00481800 0.08336500

C 36.57343500 100.48436200 0.52266000

O 36.99218600 100.82051900 1.62872300

C 35.28271300 98.30393000 0.24363300

O 34.81111500 98.40661700 1.58629600

H 37.40146700 97.89867600 1.70852000

H 36.89046300 98.96189400 -0.98416100

H 34.55745100 98.76529900 -0.44306000

H 35.40339600 97.24597000 -0.04614700

H 33.89789200 98.08577100 1.60946200

N 36.03780300 101.37512000 -0.33447400

C 35.86983900 102.75877700 0.10311600

C 34.76951400 102.83689400 1.17060300

O 33.80074400 102.08258900 1.15311100

C 35.46421800 103.49668700 -1.18862800

C 34.75107100 102.41341600 -2.00503600

C 35.57084800 101.15467600 -1.71070400

H 36.81065600 103.14135600 0.52377500

H 34.83975700 104.37754600 -0.98486100

H 36.37276200 103.83962700 -1.70698800

H 33.72550700 102.27797200 -1.63162100

H 34.70488800 102.63898800 -3.07921600

H 34.96852100 100.24173900 -1.79781800

H 36.43280800 101.06345800 -2.39408600

N 34.94336600 103.81431100 2.08593900

C 33.93797600 104.18414200 3.06331200

C 33.97766800 105.70650200 3.20534300

O 35.04230400 106.30994000 3.05919900

C 34.17099800 103.55703800 4.45643900

C 34.21337600 102.02182600 4.44522200

C 35.63296600 101.47267200 4.30284900

N 35.59311800 100.04303500 4.02357500

C 36.60134500 99.19798900 4.23809200

N 37.70816300 99.56328500 4.89721100

N 36.49593200 97.92603900 3.82779600

H 35.74822900 104.43409300 2.03822900

H 32.96929100 103.83991100 2.67663600

H 35.10472800 103.97083400 4.87296300

H 33.36797600 103.90789500 5.12060900

H 33.77166300 101.62319300 5.37144600

H 33.59871700 101.64920700 3.61106700

H 36.17700400 101.98581100 3.49684100

H 36.17778900 101.63754500 5.24487800

H 34.95299600 99.74911800 3.28580100

H 37.91600600 100.54182900 5.05651800

H 38.47638300 98.89164600 4.91724700

H 35.71957600 97.65524700 3.22899500

H 37.24420800 97.27970300 4.06479300

N 32.83578600 106.33042700 3.55514300

C 32.87414600 107.74137200 3.91263500

C 33.59934900 107.92440300 5.25200100

O 33.72207000 107.01930400 6.06584800

C 31.38502700 108.12171700 4.04207800

C 30.74067900 106.81520900 4.51968500

C 31.50610900 105.73211400 3.75216200

H 33.39489600 108.32470500 3.13977800

H 31.23520200 108.95833400 4.73740000

H 30.99854500 108.41303200 3.05345400

H 30.90666300 106.69095200 5.60020400

H 29.65921500 106.77143300 4.33165700

H 31.56483700 104.79142500 4.31544800

H 31.03758600 105.51879500 2.77653400

N 34.04860600 109.18653700 5.47617800

C 34.39940000 109.61040900 6.80755600

C 33.11637500 109.85747700 7.63134600

O 32.01090500 109.91236700 7.09905500

C 35.21925500 110.91466300 6.75699500

O 34.32515100 111.89658400 6.22391800

C 36.48608100 110.80410500 5.92285000

H 33.74783700 109.92121600 4.84295600

H 34.99079300 108.81635900 7.28554700

H 35.48232000 111.20145400 7.78831700

H 37.00012300 111.77404300 5.88323400

H 36.24973400 110.49400300 4.89509300

H 37.16650700 110.05931100 6.36232000

N 33.32133000 110.07204000 8.94987600

C 32.35150600 110.70700000 9.81246300

C 32.85748600 112.10094500 10.18477200

O 34.06210300 112.36384400 10.16407400

C 32.14000800 109.87604000 11.10752600

O 31.28683300 110.50983300 12.02913000

H 34.27903300 110.10478200 9.28756500

H 31.39990600 110.77177500 9.27074100

H 31.68579200 108.91752300 10.81365800

H 33.12980300 109.65473800 11.54631700

H 31.81181500 111.12430000 12.57252800

N 31.91919200 112.96059600 10.62921200

C 32.25715900 114.18428600 11.34002600

C 32.39968300 113.90372100 12.85388700

O 31.88570600 114.60481300 13.70551000

C 31.25391300 115.30558600 11.04620100

C 29.81942800 114.89993500 11.19822500

N 29.02333300 114.64096100 10.10247600

C 29.00260100 114.66984600 12.29267400

C 27.79494800 114.27927000 10.56108200

N 27.74516100 114.28780000 11.88260000

H 30.97522600 112.60563500 10.75952400

H 33.25468200 114.48626700 10.98456000

H 31.48673500 116.14368300 11.71657200

H 31.41941200 115.63942700 10.01289800

H 29.36688000 114.67456000 9.12674700

H 29.27473900 114.76419400 13.34221800

H 26.97612900 114.02198500 9.88890500

N 33.12875700 112.77765900 13.15132100

H 33.84079900 112.48132400 12.48498100

H 33.37665300 112.65852200 14.13050400

O 35.34425600 113.98839300 6.41730600

C 34.29411700 113.16214500 6.83729700

C 32.94137900 113.78625300 6.48655300

N 31.85697900 112.91155400 6.87131400

C 32.87093400 114.12121000 4.99343400

O 31.63732700 114.80680500 4.83304500

C 34.08660100 114.96063700 4.55193300

O 34.01853800 116.26439500 5.08220300

C 35.36335800 114.26062300 5.00924300

C 36.63876000 115.05326600 4.77318600

O 37.76364000 114.35386300 5.26928900

C 30.68749900 113.34478500 7.38916000

O 30.51682000 114.50366700 7.78671500

C 29.57747700 112.31974600 7.42111200

H 34.40684800 113.06078500 7.92759100

H 32.84116300 114.72616100 7.04293900

H 31.91319000 111.94180000 6.56392600

H 32.85416900 113.18444000 4.40748000

H 34.11124600 114.99026200 3.44658400

H 33.25710900 116.67892000 4.63962600

H 35.43344400 113.30210000 4.46818900

H 36.78162000 115.20203600 3.69206900

H 36.53207500 116.04697900 5.24345000

H 37.55454700 114.14065700 6.19174600

H 28.90044700 112.55163700 6.58273300

H 29.00788300 112.41092600 8.35573600

H 29.95108900 111.29427900 7.30453000

C 31.24769700 115.15505700 3.56060200

C 29.72927700 115.33038700 3.53532200

C 29.28805300 115.85200200 2.16634500

C 30.03524400 117.14031800 1.81374800

C 31.54092300 116.92089400 1.95807700

C 32.35696800 118.20154500 1.79455700

O 29.00919500 114.16957000 3.89750800

O 27.90489100 116.14373200 2.17222100

O 29.61940600 118.18030000 2.67422400

O 31.86132900 116.40149200 3.24577900

O 32.15886400 118.80311600 0.53566600

H 31.56946400 114.39420600 2.81607700

H 29.49029900 116.09094700 4.29303800

H 29.52458900 115.08394100 1.40165200

H 29.83453400 117.39790500 0.75657000

H 31.85041100 116.19161600 1.18274100

H 32.02245600 118.92002300 2.55664200

H 33.41864000 117.96420800 1.99240400

H 29.24194700 113.44783900 3.29342500

H 27.47375800 115.44843300 2.69413400

H 28.65710300 118.07760000 2.75556500

H 32.55260600 118.23400300 -0.14044500

L5

C 43.75041900 92.01704100 0.54493200

O 43.15177700 91.11750900 -0.04198000

C 45.17757500 91.85191900 1.02823000

H 45.59558600 92.76399300 1.47548100

H 45.80551600 91.53978600 0.18056800

H 45.20578600 91.04099100 1.77225800

N 43.17768700 93.21594100 0.80711800

C 41.81754600 93.52853200 0.42977000

C 41.67928600 95.05045000 0.42046000

O 42.43085000 95.74452400 1.10973900

C 40.77987700 92.93187800 1.41319000

C 40.94354800 93.40063400 2.85998500

C 39.73105100 93.00163700 3.70163800

N 39.79293400 93.45657400 5.08668900

C 39.24778200 94.58478300 5.55541900

N 38.69168400 95.47273100 4.72937900

N 39.23264600 94.80578300 6.87751100

H 43.68071700 93.95568300 1.28841900

H 41.62793700 93.10472300 -0.56691100

H 39.77076100 93.19569100 1.06144600

H 40.86151000 91.83577100 1.35064000

H 41.85785300 92.97147600 3.30019600

H 41.05444000 94.49336500 2.89320300

H 38.80974700 93.40215700 3.25290300

H 39.61911500 91.90719900 3.72240400

H 40.30480300 92.88019600 5.74567900

H 38.95141200 95.55433700 3.73643300

H 38.25067800 96.30280800 5.11748800

H 39.48597500 94.07880300 7.53512700

H 38.91198100 95.68938600 7.25376200

N 40.64793800 95.59346100 -0.26152800

C 40.27974100 96.97781900 0.02557600

C 39.90849100 97.09459000 1.50627600

O 39.37295900 96.15672400 2.10185300

C 39.05593600 97.23124100 -0.88028200

C 38.45265600 95.83387700 -1.06341400

C 39.68341100 94.92944600 -1.15066700

H 41.11101600 97.66274500 -0.19498700

H 38.35949100 97.95443000 -0.43401600

H 39.39939600 97.63881900 -1.84293700

H 37.85868300 95.56202400 -0.17827400

H 37.81071700 95.75438500 -1.95137300

H 39.47183900 93.90279500 -0.83022000

H 40.08397800 94.89047800 -2.17810600

N 40.16536300 98.27865300 2.08863100

C 39.82744000 98.51714700 3.47472900

C 38.29501900 98.56603000 3.64655600

O 37.52012000 98.61693100 2.70866700

C 40.54573600 99.75803700 4.04611100

C 39.87128500 101.10098300 3.85188600

C 39.04846400 101.39728600 2.75718100

C 40.04804800 102.09531300 4.82712600

C 38.37167600 102.61697300 2.66929800

C 39.39212200 103.31949900 4.74635900

C 38.52158500 103.56873600 3.68111400

O 37.79749500 104.73089000 3.71176300

H 40.62931600 99.00421100 1.55407200

H 40.17824900 97.64674000 4.05049900

H 40.68637400 99.59675200 5.12470000

H 41.56607800 99.75873400 3.62461700

H 38.88426500 100.66056400 1.97076400

H 40.70001500 101.89695400 5.68142100

H 37.70727400 102.81496200 1.82375200

H 39.51726300 104.08158100 5.51663400

H 37.32751100 104.85578200 2.87345500

N 37.87045200 98.49724600 4.94955900

C 36.48158900 98.81966100 5.24993800

C 36.31607700 100.34793500 5.20836900

O 37.18836300 101.06618500 5.70787600

C 36.06260900 98.22361900 6.58887000

O 36.90539300 98.73116300 7.61374100

H 38.54981000 98.74657400 5.66503500

H 35.87271800 98.35105400 4.46876700

H 35.01038200 98.48899800 6.78387800

H 36.13413600 97.12351900 6.52416000

H 36.54476200 98.48289600 8.47664300

N 35.24249200 100.86809700 4.59744000

C 35.13946600 102.32159900 4.41166900

C 34.97400300 103.04145100 5.75518700

O 34.21237000 102.62225800 6.62014400

C 33.90008200 102.47339000 3.50662800

C 33.05643600 101.23726500 3.83601200

C 34.10463000 100.13850800 4.01707500

H 36.05126700 102.68151900 3.92132300

H 33.37109900 103.42028900 3.67970000

H 34.22115400 102.45512300 2.45394000

H 32.52248900 101.39386000 4.78446000

H 32.32417900 100.99055600 3.05527900

H 33.76257200 99.33851600 4.68685300

H 34.39607200 99.68878700 3.05258600

N 35.70266200 104.17901600 5.87591300

C 35.79757200 104.92019400 7.12548400

C 35.55904400 106.41834500 6.89137100

O 36.39475800 107.26033200 7.21533200

C 37.14158400 104.66572300 7.82746100

C 37.33261600 103.17433500 8.08566400

C 38.77013300 102.70696400 8.31813100

N 38.83272000 101.29322200 7.95585500

C 39.84419400 100.46441800 8.18091900

N 40.97423800 100.88520200 8.75353400

N 39.70917600 99.17748600 7.78926600

H 36.36918000 104.41752900 5.14013400

H 34.98198800 104.54106300 7.76041700

H 37.94581500 105.05526600 7.18204900

H 37.17770000 105.25863900 8.75298700

H 36.69016200 102.83628500 8.91445700

H 36.99304100 102.64381900 7.19042100

H 39.46417700 103.29196900 7.68937000

H 39.08618500 102.83675400 9.36579300

H 38.13846500 100.98259300 7.26078900

H 41.14072200 101.86905500 8.92389600

H 41.69994500 100.23369900 9.02605600

H 38.74888700 98.81228800 7.76028100

H 40.44733400 98.51548900 8.00079200

N 34.38465100 106.77067400 6.31558000

C 34.06969500 108.18314400 6.16306300

C 33.84630200 108.84333200 7.53220300

O 33.57882100 108.21512200 8.54575400

C 32.75977800 108.17257200 5.34633200

C 32.08636600 106.87783000 5.81284900

C 33.26047200 105.90327800 5.93546100

H 34.88386600 108.71222400 5.64589400

H 32.15085200 109.06730100 5.52768600

H 33.00202800 108.12386500 4.27329000

H 31.61975400 107.03581900 6.79666200

H 31.31550900 106.51487200 5.11886800

H 33.09172100 105.10980800 6.67609100

H 33.46988300 105.41780200 4.96871800

N 33.91186200 110.20079900 7.49720100

C 33.29098900 111.00067000 8.52556600

C 31.78481300 110.68460600 8.61515700

O 31.19183200 110.14309000 7.68553800

C 33.45750400 112.49517100 8.15550500

O 32.92947700 112.60321800 6.82990000

C 34.89944200 112.97574000 8.20716100

H 33.93821300 110.63946200 6.58139500

H 33.76163500 110.81031700 9.50215200

H 32.83266500 113.09215800 8.83625500

H 34.94313500 114.02829400 7.89299900

H 35.53678900 112.38141000 7.53628000

H 35.29635100 112.89560000 9.23023800

N 31.14908300 111.12895300 9.72809300

C 29.74691100 111.49751500 9.66267900

C 29.64314600 112.93207000 9.14259800

O 30.58169000 113.71666300 9.26677100

C 29.05182200 111.41935000 11.04393000

O 27.69105100 111.75410000 10.96555200

H 31.70511500 111.66551600 10.38804500

H 29.24029600 110.80603000 8.97795600

H 29.14018900 110.38253100 11.40458900

H 29.59976700 112.06832100 11.75370000

H 27.58462300 112.73230200 10.95184800

N 28.45222700 113.28828500 8.60880900

C 28.14356600 114.69277300 8.48464500

C 27.68781800 115.24744400 9.84430900

O 27.33595500 114.51001900 10.77005900

C 27.15832400 114.98558400 7.33470800

C 25.99297600 114.05144300 7.27614900

N 25.90884100 113.08127500 6.30133000

C 24.86551400 113.86965100 8.05804200

C 24.77016000 112.37097800 6.52226700

N 24.11223000 112.82195200 7.57686700

H 27.67048700 112.65041000 8.73802400

H 29.08545400 115.19386300 8.25432000

H 26.82611100 116.02928600 7.42472600

H 27.71708400 114.90068200 6.39295900

H 26.64947400 112.93017300 5.59770400

H 24.56852900 114.44174000 8.93684400

H 24.47023600 111.53818600 5.88624200

N 27.71718500 116.58500800 9.92007300

H 28.15326200 117.13268700 9.17258900

H 27.44977200 117.04225800 10.78520200

O 32.94875200 114.81078500 6.04893100

C 32.16266700 113.73270200 6.47593500

C 31.20944300 113.26071000 5.36862700

N 30.38396000 112.16676300 5.81800500

C 32.05750600 112.85788000 4.16156600

O 31.20161700 112.38372400 3.13253900

C 32.86508200 114.06193000 3.67948400

O 32.00094200 115.02908400 3.12222800

C 33.68709600 114.62608800 4.84604500

C 34.42770900 115.94230200 4.52642500

C 29.06285500 112.06422200 5.57206100

O 28.40135500 112.96194000 5.03208100

C 28.41236100 110.77598200 6.02307600

O 33.85079500 117.14604200 5.00347700

H 31.60684600 114.09538200 7.35037500

H 30.55727300 114.09419600 5.08216200

H 30.84802900 111.38396100 6.28086600

H 32.74848200 112.05605900 4.46279800

H 33.58951000 113.71084400 2.92071300

H 31.43091800 114.55686000 2.49533300

H 34.46897600 113.86631300 5.02678300

H 35.41223200 115.91334000 5.01227000

H 34.59557500 115.99642400 3.43645100

H 27.89951700 110.33107800 5.15866500

H 27.64576500 111.00788400 6.77603000

H 29.12965000 110.06434400 6.44777100

C 31.56460700 111.17956600 2.55595300

C 30.33481900 110.50472100 1.94957700

C 30.75459300 109.23411600 1.20725100

C 31.85322100 109.53570800 0.18962700

C 32.99678100 110.27811600 0.88088300

C 34.08076300 110.74146800 -0.08950800

O 29.32538400 110.21428300 2.89739400

O 29.65678100 108.67977300 0.51069800

O 31.33017200 110.32910200 -0.85600700

O 32.52619100 111.43980900 1.55323000

O 34.64367700 109.66800700 -0.81066600

H 32.01279000 110.51067100 3.32250100

H 29.89539100 111.20779300 1.22687600

H 31.15261300 108.51292900 1.95083000

H 32.25800400 108.58307400 -0.20133600

H 33.44696500 109.58109200 1.61812000

H 33.61936800 111.42013800 -0.82112900

H 34.83714100 111.31380200 0.47834100

H 29.69021300 109.62029800 3.57097200

H 30.45935800 109.94980400 -1.05709500

H 35.13841500 109.11237600 -0.19211700

H 28.88183000 108.76825300 1.08756500

C 32.64521300 117.63429200 4.46581700

H 32.49001300 117.24827600 3.45003300

C 32.75207600 119.17389900 4.45775800

H 31.89118600 119.55759400 3.88694300

C 32.66296500 119.76094800 5.87491200

H 33.57444800 119.46862100 6.43136700

C 31.46330300 119.15461000 6.58923600

H 30.55205700 119.43875900 6.03018000

C 31.56381700 117.63545600 6.56989100

H 32.52673400 117.31928100 6.99717000

C 30.44054600 116.94043000 7.34979900

H 30.85831700 116.50411500 8.27291500

H 30.07655200 116.11482900 6.72337900

O 29.32334100 117.76808500 7.65488500

H 29.69123600 118.56521400 8.08232100

O 31.52214700 117.22926900 5.20122200

O 31.37080700 119.59943800 7.93457500

H 31.40452700 120.56887400 7.91034700

O 32.53209200 121.16767600 5.88350000

H 32.66224300 121.49928300 4.96772800

N 33.96358700 119.56282300 3.75540300

H 34.72521500 118.89250900 3.75754500

C 34.13177600 120.77954200 3.19716200

C 35.39878800 120.99333900 2.40488100

H 35.12420500 121.21945500 1.36316700

H 35.92324600 121.87395400 2.80392400

H 36.07106800 120.12559100 2.41904500

O 33.28587300 121.67623700 3.31790300

L6

C 43.61144100 94.23144900 6.94327300

O 42.91245700 93.58998700 7.72519100

C 45.11633200 94.32247400 7.09672400

H 45.60356100 94.89270900 6.29431700

H 45.53214900 93.30429000 7.12532600

H 45.34344100 94.79587300 8.06421100

N 43.08629500 94.90821900 5.89326000

C 41.66801700 94.91965800 5.60689900

C 41.51207300 95.32853300 4.14085800

O 42.39168500 95.98567500 3.58108400

C 40.90229600 95.88870700 6.53770700

C 41.27777600 97.35536200 6.34178800

C 40.66896600 98.23527100 7.42590300

N 40.96499200 99.64173900 7.16581700

C 40.55542700 100.65844000 7.92800700

N 39.77863400 100.43075800 8.98965800

N 40.90741900 101.90983500 7.61321600

H 41.27021100 93.90786800 5.77462700

H 39.81850000 95.75996200 6.39467100

H 41.12131700 95.56847500 7.56770400

H 42.37403800 97.46614000 6.36061100

H 40.93475800 97.70197800 5.35383600

H 39.57615400 98.09031300 7.45390300

H 41.06767400 97.94643600 8.41322700

H 41.54657600 99.86063700 6.36376400

H 39.49014800 99.49460100 9.24186700

H 39.48466700 101.18765700 9.59504800

H 41.53764200 102.09693500 6.84296700

H 40.62428600 102.69573800 8.18562200

H 43.67194900 95.41625900 5.23673100

N 40.36718200 94.99572200 3.50923000

C 40.15368600 95.46640300 2.14799500

C 39.95207600 96.97735900 2.11982900

O 39.55226100 97.60747200 3.10982000

C 38.86399300 94.73948600 1.70095600

C 38.12406400 94.49086500 3.02009700

C 39.24937500 94.18137400 4.01066500

H 41.01433200 95.21865400 1.50967100

H 38.28702200 95.33917700 0.98409000

H 39.13892900 93.78875600 1.22078600

H 37.58755800 95.40030100 3.33138400

H 37.39258700 93.67405700 2.95498700

H 38.98428900 94.45060100 5.04036100

H 39.51953200 93.11189400 3.99247800

N 40.14917700 97.57326500 0.93669500

C 39.59001400 98.88495400 0.68102600

C 38.05318600 98.79897900 0.77541800

O 37.44600400 97.79008300 0.42784900

C 40.04667900 99.42690000 -0.69328100

C 39.71544600 98.52801300 -1.85924300

C 38.46049700 98.58212200 -2.48469600

C 40.64317400 97.58432300 -2.33069900

C 38.13602500 97.72477400 -3.53491000

C 40.33113000 96.71491000 -3.37664600

C 39.07000000 96.77856800 -3.98525500

O 38.80971600 95.91789700 -5.00005100

H 40.36183400 96.99117700 0.13076300

H 39.96007600 99.58031100 1.44778100

H 39.57967300 100.41415000 -0.82245900

H 41.13378800 99.58809600 -0.63673200

H 37.72031400 99.31209800 -2.14865200

H 41.63970100 97.53511900 -1.88195900

H 37.15358300 97.78642000 -4.01099200

H 41.05833400 95.98597100 -3.73942400

H 37.91419000 96.06615800 -5.33666900

N 37.45554100 99.91892900 1.21838900

C 36.02205700 100.12628700 1.22770000

C 35.81929900 101.60179200 1.58638000

O 36.72447500 102.23360800 2.13985900

C 35.28568700 99.20323300 2.22551700

O 35.64015900 99.45786900 3.56499700

H 37.99201400 100.70660700 1.57780300

H 35.61346800 99.91388500 0.22549300

H 34.20163200 99.36208400 2.11666800

H 35.50182200 98.16578100 1.93030300

H 36.25658500 98.77474000 3.86391300

N 34.62535700 102.16297100 1.31718300

C 34.39176200 103.55656700 1.71832800

C 34.26086600 103.63286500 3.24513000

O 33.36110600 103.05065900 3.84250100

C 33.07955300 103.92470600 1.00246400

C 32.36726500 102.57867600 0.82544600

C 33.51311700 101.61066600 0.52806000

H 35.23299000 104.18064600 1.38570300

H 32.48845600 104.65662600 1.56924700

H 33.31877500 104.36815600 0.02409000

H 31.88029800 102.29222700 1.76797900

H 31.61467400 102.58845300 0.02510800

H 33.27677500 100.58134100 0.82259400

H 33.77544700 101.61033000 -0.54409000

N 35.18396600 104.41177500 3.85530900

C 35.25727600 104.57633900 5.29706200

C 35.25252200 106.07126100 5.65999500

O 36.17721500 106.58521400 6.28685700

C 36.46255400 103.85833800 5.90548300

C 36.45677900 102.35247900 5.64688000

C 37.59382700 101.66292500 6.40102300

N 37.57301000 100.20737100 6.28435500

C 38.12569100 99.49060900 5.30415800

N 38.95678000 100.02315500 4.41197000

N 37.84849600 98.17541100 5.20191400

H 35.96727600 104.74050200 3.30085800

H 34.33451700 104.12397800 5.69082800

H 37.38910400 104.31310400 5.51703900

H 36.45990300 104.05936900 6.98817000

H 35.49831800 101.91639700 5.97371900

H 36.54643600 102.14296100 4.57024000

H 38.57428000 102.03745800 6.07163100

H 37.53628500 101.89074000 7.47509700

H 36.91189800 99.71959400 6.88047000

H 39.15255500 101.01442400 4.38577700

H 39.35622900 99.39727300 3.70727100

H 37.33326500 97.70143300 5.93458500

H 38.40486000 97.61951100 4.54330500

N 34.18486800 106.79739300 5.25226100

C 34.12477500 108.21296800 5.58745400

C 34.00230700 108.44022000 7.10061300

O 33.48281000 107.64136300 7.86529300

C 32.84407800 108.70527600 4.87565500

C 31.97088600 107.44770900 4.82560100

C 32.98405500 106.33912400 4.54159800

H 35.02545800 108.73196500 5.22589700

H 32.37486300 109.53508000 5.41899600

H 33.09850800 109.05197700 3.86369700

H 31.49936100 107.28197300 5.80610500

H 31.18151700 107.49809900 4.06279900

H 32.65964500 105.34933100 4.88825300

H 33.18865800 106.26450800 3.46030000

N 34.45832900 109.66173400 7.48415400

C 34.02118000 110.31019700 8.69741100

C 32.49206000 110.49464400 8.69378800

O 31.83443400 110.42208000 7.65871200

C 34.66893100 111.71845800 8.73907600

O 34.29903100 112.28166300 7.48086400

C 36.18025200 111.68203200 8.91398300

H 34.70206100 110.30910200 6.74097300

H 34.32145400 109.73076700 9.58367600

H 34.19949800 112.30413300 9.54341900

H 36.59584000 112.69324200 8.80326700

H 36.64771600 111.03360200 8.15861400

H 36.44039600 111.29878900 9.91204000

N 31.94280900 110.82892400 9.88767500

C 30.68377100 111.53429200 9.93244000

C 30.94314400 113.05449500 9.90492500

O 32.08923000 113.51272700 9.88647700

C 29.79774300 111.09799800 11.10425700

O 28.49070000 111.57884600 10.83632100

H 32.57246200 110.96800100 10.67207700

H 30.14167200 111.27278900 9.01337300

H 29.82100400 109.99548200 11.15669400

H 30.19088200 111.49510600 12.05729300

H 27.96809400 111.56168600 11.64946100

N 29.83965900 113.81842700 9.87730500

C 29.87043500 115.27036800 9.76688400

C 30.10627300 115.91352000 11.15376000

O 29.24040800 116.55599900 11.74914000

C 28.59631200 115.77626900 9.07062000

C 27.33523600 115.16160300 9.58517400

N 26.89616500 115.32758300 10.88038700

C 26.41021900 114.31039400 9.00707700

C 25.76022400 114.59500500 11.03304900

N 25.43295900 113.96928700 9.91616100

H 28.94434300 113.35298600 10.04602600

H 30.73435900 115.52514200 9.13522500

H 28.56853900 116.87219500 9.17216600

H 28.69564700 115.54726800 8.00167800

H 27.42620300 115.87334400 11.56380300

H 26.40540400 113.94084400 7.98166700

H 25.21447000 114.55530800 11.97573600

N 31.34036700 115.70452500 11.64320400

H 31.99284000 115.10234900 11.14189600

H 31.56152200 116.02283200 12.58079400

O 35.19765900 114.39536900 7.09121600

C 34.03341200 113.65347200 7.33998100

C 33.05136300 113.75577400 6.15844000

N 31.81854400 113.07048000 6.45538400

C 33.77014700 113.20039800 4.91484900

O 33.02527800 113.28548700 3.70388100

C 35.05458100 114.00176500 4.67446100

O 34.73879200 115.33535300 4.32629500

C 35.92612300 113.98496900 5.92786100

C 37.13568000 114.90471700 5.86945100

O 37.86429600 114.84630400 7.07914000

C 30.61313300 113.69246800 6.49152600

O 30.49242500 114.91731100 6.41254300

C 29.41281100 112.77928400 6.60205600

H 33.58517100 114.05808500 8.25727800

H 32.81244700 114.81283900 5.99394000

H 31.86143900 112.07154900 6.65544200

H 34.03499200 112.14712600 5.09843400

H 35.62219200 113.50745500 3.86211700

H 34.08716200 115.28733600 3.61033700

H 36.27538000 112.94929200 6.07668400

H 37.80302600 114.58208200 5.05559200

H 36.79254800 115.93039700 5.64361400

H 37.22071300 115.02280600 7.78240900

H 28.77465100 113.10318300 7.43440600

H 29.69539200 111.72755800 6.74057000

H 28.84290300 112.87746200 5.66600300

C 31.93283800 112.40344400 3.53487100

H 31.98751300 111.59973900 4.28203100

C 32.00084200 111.79037200 2.12951200

H 31.22140000 111.01271800 2.08744500

C 31.66795000 112.83355200 1.06301700

H 32.43967000 113.62595400 1.08359700

C 30.32497000 113.48143700 1.40519400

H 29.54786600 112.69265100 1.40107400

C 30.38065600 114.07544400 2.81117600

H 31.16230300 114.85573400 2.83387100

C 29.08850800 114.71063800 3.28038800

H 28.85410300 115.53566700 2.58350300

H 29.26154200 115.13055400 4.28583700

O 28.06235900 113.73204300 3.29762300

H 27.26018900 114.14851800 3.63820300

O 30.71065100 113.03558200 3.73581900

O 30.01874200 114.49968000 0.47773400

H 30.15166100 114.10699900 -0.39900600

O 31.61676900 112.29256500 -0.24242600

H 31.71717800 111.31969100 -0.17858600

N 33.27765300 111.12683900 1.94106900

H 34.07813100 111.48535800 2.45000200

C 33.42778500 110.09030200 1.08275700

C 34.74238900 109.35117200 1.14479700

H 35.53384400 109.92641000 1.64386500

H 34.58506900 108.41622700 1.70746300

H 35.05740600 109.08111300 0.12807400

O 32.51615100 109.74254600 0.32414800

L7

C 46.22531100 96.54266200 1.10749200

O 46.30999000 95.61796600 1.91371200

C 47.43776500 97.08974800 0.38062000

H 47.18788100 97.84113200 -0.38066600

H 47.97585200 96.25466600 -0.09145700

H 48.11753300 97.54113300 1.11996200

N 45.04718100 97.14465000 0.81901600

C 43.80262300 96.76195500 1.44685200

C 42.66214000 97.30264300 0.58370500

O 42.85237300 98.26885600 -0.15932200

C 43.65880100 97.34243300 2.87537400

C 43.73145900 98.87023700 2.92228100

C 43.11489400 99.41215300 4.20341100

N 43.17063600 100.87430600 4.21416100

C 42.51837600 101.64865200 5.07277900

N 41.81717400 101.11603700 6.07330900

N 42.50969900 102.99203800 4.90503100

H 43.76766400 95.66508000 1.51613300

H 42.69436400 97.01385100 3.28928100

H 44.44637200 96.89409300 3.49927000

H 44.77873900 99.20301900 2.83509700

H 43.17818200 99.30024800 2.07559600

H 42.05892700 99.10282600 4.24241200

H 43.63639000 99.01181600 5.09036100

H 43.75248600 101.32765100 3.51689000

H 41.83411800 100.12100300 6.25799700

H 41.28136800 101.69952800 6.70476300

H 43.12451100 103.39801300 4.20633800

H 42.36264800 103.57549100 5.72373500

H 44.98297500 97.90552500 0.14864000

N 41.43583200 96.76702200 0.75508300

C 40.27433400 97.49666800 0.26173800

C 40.10965600 98.80220500 1.05576100

O 40.58701400 98.95842400 2.17375100

C 39.09594600 96.54033900 0.53800400

C 39.56442000 95.76555600 1.77470400

C 41.06376300 95.57429200 1.53124100

H 40.38216700 97.73449500 -0.80632600

H 38.15865100 97.08771100 0.70249900

H 38.96532500 95.86642400 -0.32234400

H 39.40997200 96.37627800 2.67686300

H 39.03991700 94.81001700 1.91206900

H 41.62903700 95.50152900 2.46822000

H 41.26478100 94.66414900 0.94085300

N 39.35476800 99.74369300 0.44041600

C 38.82159100 100.85981600 1.18809100

C 37.75711900 100.35512700 2.18562900

O 37.37245600 99.19310000 2.20506100

C 38.25612900 101.96613700 0.25191700

C 36.75081100 101.95949800 0.08257000

C 36.04658700 100.80138600 -0.27980100

C 36.00775900 103.11499800 0.36539900

C 34.65064100 100.78478800 -0.32591300

C 34.61655700 103.12135100 0.30885100

C 33.93493800 101.94696100 -0.02183200

O 32.56575200 101.99247100 -0.03259200

H 38.92291900 99.50798100 -0.44600500

H 39.64645700 101.27945700 1.78325400

H 38.55834100 102.94671600 0.64430600

H 38.76674100 101.86517200 -0.71970200

H 36.58165700 99.87702000 -0.50439800

H 36.52656800 104.02793200 0.66595400

H 34.11988300 99.86839500 -0.59589200

H 34.04951400 104.02649800 0.51991300

H 32.19038200 101.13717200 -0.29017100

N 37.25848900 101.30756400 3.00793500

C 36.04256600 101.08466400 3.75974300

C 35.15675000 102.32654600 3.62304100

O 35.67812400 103.44471600 3.60316500

C 36.32817300 100.77654300 5.23497700

O 37.17377100 101.78822600 5.77539900

H 37.53278800 102.28028900 2.89789700

H 35.55679900 100.19741300 3.33594100

H 35.37734100 100.72695900 5.79211900

H 36.82564500 99.79465200 5.29152400

H 37.40359300 101.54995100 6.68541100

N 33.82564500 102.13903900 3.57684400

C 32.91771200 103.28566400 3.64984400

C 32.95124600 103.83967700 5.08525000

O 33.15327000 103.10389400 6.04654300

C 31.55654100 102.67832900 3.27589600

C 31.65086100 101.25023100 3.82389500

C 33.10654500 100.85420400 3.55165600

H 33.22857100 104.06755300 2.94766500

H 30.71847800 103.25949500 3.68157800

H 31.47201400 102.66689300 2.17961900

H 31.46616300 101.24955100 4.90781600

H 30.94115600 100.55748900 3.35098700

H 33.49085600 100.15969900 4.31074000

H 33.22137400 100.38413100 2.56056100

N 32.76644900 105.17861200 5.19311800

C 33.08984400 105.88477500 6.42898200

C 32.07141700 106.99952900 6.66287900

O 32.33544200 108.15476500 6.31124800

C 34.51729400 106.45287200 6.38993100

C 35.54197800 105.33973800 6.23226400

C 36.96019200 105.79518700 5.91595400

N 37.73849200 104.59907100 5.60770300

C 38.88704800 104.56447800 4.94197500

N 39.57090200 105.68477400 4.68899700

N 39.34370600 103.37827600 4.49887300

H 32.59274400 105.71170200 4.33555700

H 33.02306200 105.13720000 7.22932200

H 34.58504500 107.15810100 5.54703800

H 34.69392900 107.03631500 7.30750800

H 35.55464100 104.70268400 7.13215000

H 35.24422000 104.70132900 5.39239000

H 36.95268200 106.47003000 5.04200700

H 37.41320900 106.34581900 6.75879500

H 37.25662900 103.69791900 5.68128800

H 39.26562600 106.57842600 5.05201800

H 40.42554800 105.66549200 4.14707000

H 38.84657600 102.54157100 4.80826300

H 40.32312200 103.28352600 4.24360300

N 30.89013000 106.67911900 7.22392000

C 29.77905200 107.65487300 7.26226400

C 29.91016800 108.67449800 8.41882400

O 29.16687100 108.66510600 9.39526000

C 28.53961700 106.76868400 7.37900400

C 29.04508800 105.54816200 8.15570700

C 30.44521100 105.32076100 7.58269800

H 29.79614200 108.22410700 6.32391900

H 27.71658600 107.28948600 7.88278200

H 28.22347100 106.48366200 6.36526000

H 29.11203900 105.79357300 9.22654800

H 28.40600600 104.66134700 8.04387700

H 31.13213700 104.85680400 8.30402200

H 30.41727100 104.68770200 6.68112800

N 30.88980900 109.58280400 8.22132600

C 31.11268200 110.72914700 9.06060200

C 30.64739100 112.00298500 8.31215800

O 30.17006900 111.96264400 7.18545100

C 32.61703100 110.85638900 9.40362700

O 33.22885300 111.03386700 8.13201700

C 33.17671400 109.63803400 10.12186800

H 31.44317800 109.49897600 7.36417000

H 30.52147400 110.60545700 9.98011700

H 32.78746200 111.75474700 10.02034700

H 34.25838400 109.75249000 10.27896400

H 33.00579600 108.73237300 9.52229100

H 32.68876500 109.50936700 11.09984800

N 30.84983100 113.16017200 8.99122800

C 30.89685400 114.45576200 8.35600900

C 32.28394800 115.05295200 8.58887100

O 32.97879300 114.68521000 9.53912600

C 29.82027200 115.40716700 8.94689300

O 29.90979900 116.72000800 8.44489600

H 31.34571300 113.12132800 9.87661900

H 30.70543000 114.32511700 7.28384000

H 28.83568000 114.99739000 8.67429800

H 29.89820500 115.37947600 10.04860900

H 30.55569700 117.21593300 8.97864200

N 32.63543800 116.06104800 7.76391600

C 33.74662700 116.95231700 8.06173100

C 33.26600800 118.14195500 8.92360800

O 33.56865200 119.29585600 8.68229600

C 34.47416200 117.40645700 6.79142200

C 33.57351300 117.94086200 5.71989000

N 33.25724100 117.19268900 4.60580200

C 32.88375800 119.13064400 5.55625300

C 32.41649600 117.93442000 3.83576900

N 32.17047500 119.11493000 4.37882100

H 31.93020000 116.40919000 7.11943500

H 34.44356100 116.37660500 8.69028800

H 35.20984700 118.16712600 7.08548300

H 35.02119000 116.54080500 6.39369500

H 33.57551700 116.21718600 4.46319300

H 32.87648100 119.98065600 6.23578600

H 32.01477600 117.56508800 2.89201800

N 32.44380000 117.78606900 9.96509200

H 32.55058800 116.85230500 10.35884500

H 32.24420700 118.51949400 10.64081300

O 35.54637000 111.30569600 8.31057500

C 34.32214500 111.88685400 7.93295200

C 34.31352600 112.17283700 6.42116400

N 33.07979900 112.80101500 6.01485100

C 34.47472100 110.80733600 5.73874800

O 34.32627200 110.90306500 4.32960900

C 35.82468000 110.20560000 6.12217800

O 36.89600400 110.99556600 5.64128900

C 35.87612800 110.07773100 7.64314400

C 37.22284200 109.66626400 8.21116600

O 37.16415300 109.58222300 9.62144900

C 33.02414800 113.84575600 5.16784800

O 34.00474900 114.55153900 4.88835800

C 31.65995300 114.11308000 4.56991800

H 34.22181800 112.80414900 8.53385200

H 35.14859900 112.83733900 6.17087900

H 32.27687900 112.16957200 5.99034200

H 33.69444500 110.15168600 6.13640800

H 35.88133600 109.18101700 5.70584500

H 36.70119900 111.17017700 4.70723600

H 35.12786800 109.31742400 7.92341200

H 37.49383800 108.67241600 7.82238900

H 37.99017100 110.38584000 7.87177300

H 36.78104400 110.42285400 9.91655900

H 31.38291400 115.16512400 4.72597900

H 30.88476700 113.45710600 4.98417700

H 31.72610100 113.95103400 3.48272500

C 34.00313900 109.27185700 2.49160800

C 33.92535800 110.17923800 1.22299800

O 33.72405700 109.57278400 0.13338000

O 34.09794600 111.39948200 1.38826600

C 33.51946200 109.84589500 3.82812600

H 33.58647900 109.00429700 4.53108300

C 32.02730400 110.19644900 3.73901300

H 31.88746300 110.95830600 2.94829500

C 31.28413800 108.90514300 3.37108400

H 31.42014000 108.21339200 4.20885400

C 31.89338100 108.27222100 2.11311500

H 31.73795200 108.95676100 1.26355000

C 31.29172700 106.92987200 1.72437000

H 30.24761600 107.10966100 1.40743400

C 32.05929000 106.33809000 0.52572900

H 33.00225300 105.91943900 0.93014400

C 31.24473600 105.20925300 -0.09463300

H 30.91526700 104.53900900 0.71615300

H 30.33716800 105.66562700 -0.53771900

O 31.95640100 104.50876400 -1.09372400

H 32.04648600 103.58580700 -0.80547500

O 32.30942700 107.30626600 -0.46322500

H 32.91376000 108.01666200 -0.12590300

O 31.31635000 106.00223900 2.80019300

H 30.57214000 106.25462400 3.39333200

O 33.28264600 108.05858800 2.31281100

N 29.85526200 109.15419400 3.25877300

H 29.54372100 109.99419300 2.78093700

C 28.92137500 108.25433500 3.64844400

C 27.48076300 108.68872400 3.55345900

H 26.89165800 107.87582800 3.10699400

H 27.34308700 109.61123200 2.97437100

H 27.10334000 108.85079300 4.57552200

O 29.22489800 107.14085800 4.09840100

O 31.57466200 110.69599800 4.98365100

H 30.60881600 110.67209200 5.03661100

H 35.06060800 108.98559900 2.61605400

L8

C 41.42615300 93.90393800 -2.64556000

O 40.52718800 93.21249000 -3.12100700

C 42.85436100 93.82178500 -3.14665600

H 43.52333900 94.55848100 -2.68161700

H 42.85629300 93.96312000 -4.23755300

H 43.24013900 92.81007800 -2.94713100

N 41.20807600 94.78031700 -1.63630900

C 39.90415900 94.97584100 -1.04082000

C 39.93213500 96.32779800 -0.32560300

O 41.00327900 96.80017200 0.06224900

C 39.54936500 93.85101400 -0.03835900

C 40.47252100 93.79996900 1.17620300

C 40.16756500 92.60161100 2.06547000

N 41.03627500 92.61281900 3.24050100

C 40.89673400 91.80997300 4.29754000

N 39.94510500 90.87227600 4.30426600

N 41.70126200 91.94840400 5.35672900

H 41.95220200 95.36100000 -1.26014300

H 39.15122400 94.96653900 -1.84227100

H 38.50882700 93.97715300 0.29819900

H 39.58975900 92.90396000 -0.59845600

H 41.52200400 93.74682100 0.84188900

H 40.35380000 94.71822100 1.77118000

H 39.11617900 92.64325800 2.39706900

H 40.30448000 91.66189600 1.50149700

H 41.81495700 93.26314100 3.24183300

H 39.38784100 90.68225800 3.48194000

H 39.80402100 90.27831400 5.11191500

H 42.38382000 92.69461300 5.40436700

H 41.64555900 91.31248600 6.14246000

N 38.75347100 96.93106500 -0.07410900

C 38.72367600 98.07981300 0.82298900

C 39.05934100 97.63712200 2.25313900

O 38.89672700 96.48550800 2.63927200

C 37.26343700 98.57164900 0.73765000

C 36.48451400 97.28622200 0.43744500

C 37.41064200 96.51963300 -0.51038300

H 39.44547700 98.84479600 0.50185600

H 36.94479200 99.06216700 1.66671800

H 37.16998200 99.29035400 -0.09072200

H 36.34559800 96.71008900 1.36450500

H 35.49618600 97.47113800 -0.00529100

H 37.27268000 95.43402300 -0.43619800

H 37.24725800 96.81418500 -1.56117600

N 39.49266800 98.63314000 3.06384000

C 39.50035300 98.46395500 4.49795600

C 38.05310500 98.49458600 5.04068300

O 37.08370400 98.70011100 4.31829900

C 40.45633300 99.44833500 5.18461000

C 40.13977800 100.93131700 5.10885200

C 38.96381300 101.46460800 4.56783400

C 41.07066200 101.83522300 5.65322900

C 38.70860400 102.83978600 4.59005300

C 40.83111800 103.20599400 5.68184700

C 39.63150900 103.71782700 5.16204700

O 39.41508000 105.05689200 5.26524400

H 39.52054100 99.57871800 2.69822600

H 39.87197600 97.44751100 4.69909700

H 40.54428700 99.16523500 6.24577500

H 41.45689300 99.26899300 4.75867500

H 38.20110100 100.81591400 4.14074700

H 41.99976600 101.45242400 6.08405400

H 37.77595300 103.22605600 4.17018800

H 41.55610000 103.89530800 6.11935100

H 38.55493900 105.28232600 4.88190700

N 37.91136800 98.26451300 6.36872500

C 36.61663800 98.49088000 6.98550800

C 36.25137700 99.97829200 6.81409800

O 37.12721600 100.84210500 6.91992800

C 36.62192900 98.05820900 8.44584300

O 37.57613500 98.83015600 9.16255400

H 38.73382900 98.25101600 6.96035400

H 35.87753300 97.87197200 6.46019100

H 35.61252200 98.20647200 8.86228000

H 36.86105600 96.98140200 8.49635700

H 37.45464700 98.68197100 10.11111300

N 34.98329100 100.29875000 6.51250500

C 34.65324700 101.67662700 6.12771600

C 34.79546500 102.63609600 7.31404800

O 34.32710400 102.37491900 8.41551500

C 33.19186200 101.56994700 5.64176000

C 32.64655000 100.34981100 6.39147000

C 33.83524700 99.38862600 6.39583100

H 35.32905200 101.99361200 5.32039100

H 32.62167400 102.48904300 5.83338100

H 33.18678800 101.39142000 4.55597800

H 32.39740300 100.62903000 7.42553800

H 31.75658000 99.91245000 5.91889000

H 33.80205900 98.68093900 7.23462400

H 33.89753100 98.81543300 5.45450900

N 35.43020900 103.79974800 7.02208300

C 35.79630000 104.78430300 8.03060400

C 35.37440900 106.19236800 7.58762600

O 36.19102800 107.10741000 7.48841300

C 37.29398100 104.71478300 8.36006900

C 37.66926300 103.31824600 8.84739900

C 39.15834500 102.98152600 8.77813100

N 39.29372600 101.53335700 8.64495500

C 40.39077100 100.82865200 8.89331200

N 41.54104500 101.43077600 9.21419400

N 40.32398100 99.48546400 8.81430300

H 35.86669500 103.88892000 6.11069900

H 35.21472800 104.51819800 8.92656800

H 37.86501800 104.96870600 7.45197800

H 37.53087500 105.49236600 9.10122700

H 37.28550900 103.14058300 9.86488800

H 37.16805000 102.59757500 8.19314200

H 39.61747300 103.47866200 7.90843300

H 39.69723100 103.31798200 9.67806400

H 38.52586800 101.05177600 8.15700100

H 41.66186700 102.42703400 9.08531500

H 42.35306800 100.89383700 9.49159900

H 39.40078300 99.05728100 8.94918100

H 41.14076700 98.92865000 9.03488800

N 34.06281700 106.38235600 7.31465800

C 33.60333700 107.72125400 6.96870800

C 33.76331300 108.68183600 8.15149800

O 33.68360200 108.31677800 9.31739600

C 32.10227800 107.52361100 6.65947500

C 31.72549400 106.33400000 7.54908200

C 32.95458500 105.42846700 7.45538400

H 34.16282000 108.11116000 6.10694100

H 31.51825800 108.42767100 6.87381500

H 31.97666400 107.27931500 5.59471500

H 31.59005900 106.67357500 8.58662700

H 30.80669700 105.82499600 7.22576100

H 33.08639600 104.78019200 8.33200500

H 32.89956400 104.77950200 6.56565500

N 33.89406700 109.98262000 7.79156100

C 33.56903100 111.04106200 8.71240300

C 32.13819000 110.87287800 9.25840400

O 31.26524300 110.26153900 8.65237800

C 33.75003100 112.42943000 8.01208600

O 33.53015000 112.33201200 6.60620100

C 35.15823700 112.96875000 8.18990400

H 33.87128300 110.21873600 6.80271500

H 34.24919900 111.01045200 9.57785800

H 33.01554600 113.12829400 8.44431400

H 35.26641700 113.92130500 7.65168300

H 35.89364000 112.25288600 7.79137700

H 35.37354200 113.13843100 9.25466900

N 31.89604100 111.50011900 10.43310800

C 30.53229800 111.68092900 10.88092700

C 29.81603500 112.69939000 9.97297200

O 30.39793700 113.69303500 9.55039400

C 30.49465000 112.16000600 12.34046200

O 29.18185300 112.19330400 12.84622300

H 32.62218700 112.07736700 10.84453400

H 30.01616500 110.71245700 10.81938200

H 31.06541300 111.44812000 12.95606300

H 30.99495500 113.14559200 12.40900000

H 28.68855300 112.89111200 12.39210700

N 28.51429700 112.43350500 9.74976300

C 27.65962800 113.22526400 8.89158800

C 26.22444200 112.72305300 9.12136100

O 26.00661500 111.66950900 9.71315700

C 28.08414900 113.18539800 7.40792700

C 28.20087200 111.82039100 6.80651600

N 28.91127800 111.62705600 5.64080800

C 27.77349400 110.55464200 7.17027000

C 28.89910600 110.29381900 5.35711100

N 28.21906900 109.61461300 6.26374500

H 28.08546200 111.58160900 10.10523800

H 27.71074700 114.28119500 9.20471300

H 27.39313900 113.81113100 6.81981900

H 29.05964900 113.68320200 7.32888800

H 29.39298500 112.35795300 5.10502600

H 27.18086600 110.28252100 8.04085000

H 29.43677500 109.87485100 4.50579100

N 25.25029600 113.50080300 8.61363400

H 25.43760600 114.41017400 8.21180600

H 24.28269000 113.21775800 8.72508200

O 31.40085800 113.25249100 6.35540200

C 32.20304500 112.11355400 6.17487100

C 32.28699900 111.66564200 4.70707300

N 33.23142200 110.58506500 4.51152200

C 32.58286400 112.83762100 3.77213300

O 32.44503800 112.33690100 2.45588700

C 31.59160100 113.96112800 4.06823600

O 30.26584800 113.48717500 3.82979400

C 31.75562500 114.35785400 5.52819600

C 31.06252800 115.62910500 6.00319400

C 32.87598700 109.47126000 3.78974100

O 31.70714000 109.15104400 3.61077600

C 34.02846800 108.68733600 3.21060800

O 29.65268100 115.69319000 5.89755800

H 31.72134200 111.32812400 6.77038200

H 31.30908600 111.26063800 4.43453600

H 34.20940300 110.85559200 4.47759100

H 33.60125500 113.21623000 3.94134800

H 31.80583400 114.82870700 3.43026600

H 30.26578200 113.04084500 2.96853800

H 32.83026500 114.57763000 5.66465000

H 31.27702200 115.72873500 7.07755300

H 31.53939900 116.47385200 5.48717400

H 34.17394200 109.07186800 2.18610700

H 33.76636800 107.62245600 3.16165000

H 34.96355800 108.82005100 3.77370800

C 32.20714600 112.45614200 0.07521500

C 33.18167900 111.29957600 -0.35056400

O 33.24560400 111.08957100 -1.59255000

O 33.77209900 110.67237100 0.54700500

C 32.52918500 113.23306300 1.36481500

H 31.74585600 114.00866500 1.45532600

C 33.85243900 113.98286700 1.19080900

H 34.65548700 113.23966000 1.02821600

C 33.71353900 114.88888700 -0.04626800

H 32.92948900 115.62816900 0.16765700

C 33.27196800 114.07401400 -1.26805300

H 34.05493400 113.33196500 -1.49721800

C 33.04296000 114.88784500 -2.53959900

H 34.03647200 115.22852200 -2.89426800

C 32.43636100 113.97306900 -3.62398600

H 31.37773300 113.81679900 -3.33128700

C 32.45232200 114.67069600 -4.97778400

H 32.09853700 115.70837800 -4.83837500

H 33.49525000 114.71313600 -5.33466100

O 31.71368100 113.98894600 -5.97513600

H 30.79354500 113.94953100 -5.67949000

O 33.12742800 112.75402800 -3.73160000

H 33.01615000 112.20975700 -2.90950500

O 32.17439700 115.98299700 -2.33602600

H 32.67473300 116.65347900 -1.82536200

O 32.05552000 113.41170600 -0.95821600

N 34.94778000 115.62489900 -0.26140300

H 35.82490700 115.11518100 -0.22300400

C 34.97450800 116.91492100 -0.67847300

C 36.33831500 117.54429600 -0.82807500

H 37.16370700 116.85213100 -0.61627600

H 36.40181200 118.40292800 -0.14257600

H 36.43703200 117.93357600 -1.85203100

O 33.94314000 117.55381300 -0.91536500

O 34.13187100 114.76392400 2.34247400

H 34.93226100 115.27904900 2.16490900

H 31.21807100 111.99001100 0.21488100

C 29.00839400 116.03946100 4.68353400

H 29.27739900 115.34010800 3.88658600

C 29.24715500 117.49607600 4.21081100

H 28.57850700 117.60728600 3.33777800

C 28.78908700 118.44199000 5.31691400

H 29.37247500 118.24214300 6.22961400

C 27.31717800 118.17933000 5.62470000

H 26.72592300 118.43479200 4.72342900

C 27.07778400 116.69789100 5.93237100

H 27.56042000 116.46945000 6.89815100

C 25.60554600 116.35769200 6.05381300

H 25.21384600 116.89709800 6.93474300

H 25.51962300 115.27036500 6.24784600

O 24.92722600 116.71624300 4.86917000

H 23.97817100 116.63556100 5.02901600

O 27.63565300 115.87285900 4.90710300

O 26.89528400 118.93700700 6.73781800

H 27.21083700 119.84148500 6.58909100

O 29.01668000 119.81073800 5.02354900

H 28.55757100 120.02977400 4.19739900

N 30.60052300 117.81450500 3.79363100

H 31.01752500 118.63802900 4.21536400

C 31.16415400 117.31527200 2.65998600

C 32.53987600 117.83712900 2.31926200

H 33.25261600 117.01618500 2.49040200

H 32.58790500 118.07823000 1.24821700

H 32.83710100 118.71011300 2.91589600

O 30.61176900 116.44387500 1.98741000

L9

C 43.75097800 99.50304800 -0.32023200

O 43.86372300 98.29377400 -0.14468400

C 43.81018000 100.12670500 -1.69803700

H 43.90854900 101.22062700 -1.67721300

H 42.88810200 99.86397500 -2.24053800

H 44.65427700 99.69053500 -2.25013200

N 43.52193600 100.36854800 0.70669200

C 43.42631300 99.93117100 2.08762300

C 42.14852200 100.45924100 2.75981000

O 42.18676400 101.06604900 3.83043700

C 44.68653300 100.24771900 2.90210300

C 45.09548000 101.71996200 2.89527900

C 46.32896200 101.95992900 3.75669500

N 46.71536200 103.36766400 3.71979300

C 47.87976900 103.84919000 4.16155200

N 48.77489500 103.03084800 4.72178100

N 48.15578200 105.15107900 4.03186800

H 43.43790100 101.35711400 0.50119400

H 43.33088600 98.83552700 2.03513800

H 44.51509200 99.91459100 3.93675000

H 45.50732600 99.63548000 2.49441500

H 45.31499100 102.04790500 1.86601500

H 44.26103400 102.33094900 3.27287000

H 46.12739200 101.64851500 4.79707600

H 47.16822700 101.35086800 3.38228700

H 46.01734500 104.03640900 3.41087400

H 48.54486100 102.07131600 4.94438600

H 49.68386800 103.37061300 5.01056700

H 47.53338300 105.76873600 3.52601700

H 49.00403100 105.54975700 4.41473200

N 40.97276200 100.20686700 2.13731200

C 39.73387600 100.59159000 2.80428400

C 39.55748700 99.81319700 4.11253600

O 40.00736100 98.68546900 4.27197200

C 38.63682400 100.17594500 1.80140300

C 39.26351900 98.96958500 1.09335200

C 40.73175400 99.37609300 0.94781500

H 39.72540400 101.67079800 3.01720800

H 37.69187800 99.94177400 2.30971800

H 38.45948300 100.99890400 1.09190300

H 39.18473100 98.07954600 1.73476500

H 38.79628000 98.74388300 0.12464400

H 41.41418700 98.51650000 0.91162800

H 40.88919900 99.96857300 0.03158700

N 38.79556900 100.43953200 5.04329300

C 38.25219600 99.70694500 6.16043100

C 37.17598700 98.71126300 5.67605100

O 36.72128100 98.73032900 4.53749200

C 37.77578800 100.65240900 7.27572500

C 36.73176700 101.70569000 6.94608600

C 35.86884200 101.65236400 5.84220400

C 36.58002600 102.78741700 7.83266200

C 34.87693100 102.61872200 5.64573300

C 35.58878400 103.75029800 7.65496500

C 34.70920300 103.66427200 6.56325900

O 33.73517100 104.59933900 6.44457300

H 38.37042800 101.33060800 4.81085000

H 39.06241600 99.09120100 6.58075400

H 37.40972600 100.03065800 8.10739500

H 38.67248700 101.15677000 7.66850500

H 35.94909900 100.84464600 5.11739500

H 37.23979400 102.86255800 8.70168200

H 34.23146000 102.57010500 4.76569900

H 35.46714500 104.56945800 8.36661100

H 32.98824800 104.26074100 5.89954800

N 36.74599800 97.80783200 6.59406700

C 35.52530500 97.06791200 6.31771400

C 34.40576800 98.09957700 6.06441100

O 34.38562200 99.14983200 6.71364300

C 35.19473400 96.09674200 7.44266900

O 34.92580000 96.82335800 8.63456500

H 37.04342300 97.90720000 7.55830100

H 35.68139500 96.47492700 5.40613900

H 34.31523500 95.50220800 7.14866600

H 36.04541200 95.40595300 7.57860500

H 34.52505800 96.22814600 9.28399800

N 33.51377600 97.86334300 5.09095300

C 32.67975200 98.96449200 4.58941700

C 31.71290400 99.49555800 5.65343700

O 30.97185300 98.75739600 6.29191300

C 31.94070400 98.33724500 3.38637000

C 31.98119600 96.83125300 3.66934800

C 33.36257700 96.63992700 4.29397100

H 33.34206100 99.78335400 4.27158500

H 30.92153200 98.73177100 3.27618200

H 32.49286300 98.56941400 2.46345700

H 31.20791600 96.56843500 4.40639300

H 31.83713600 96.21623800 2.77058400

H 33.42427400 95.74417700 4.92543700

H 34.15060800 96.58144700 3.52234300

N 31.73402400 100.84263700 5.77236900

C 30.93322600 101.60377100 6.70563000

C 30.63525100 102.95087800 6.05398500

O 31.50265300 103.47657500 5.33269800

C 31.69847800 101.94070300 8.01748700

C 32.40030600 100.73594400 8.63907000

C 33.65164500 101.11676300 9.43626700

N 34.59635400 100.00458200 9.40479600

C 35.62160200 99.84073900 10.23464200

N 35.89204100 100.74052300 11.18647700

N 36.39717000 98.75061000 10.09337600

H 32.41485800 101.37967300 5.24637300

H 30.02945300 101.02077400 6.92284300

H 32.44735400 102.70396000 7.76089300

H 31.00471700 102.42749700 8.71982900

H 31.71018100 100.13951800 9.25532400

H 32.74053000 100.08916800 7.82497800

H 34.12013000 102.00777100 8.98570900

H 33.40658100 101.35946000 10.48228100

H 34.59505800 99.44736900 8.53906800

H 35.43925000 101.64510200 11.19734600

H 36.60951000 100.56802900 11.87959500

H 35.99639500 97.93981300 9.60621700

H 37.17159200 98.59537800 10.72697500

N 29.50380100 103.59126500 6.36555500

C 29.38102000 105.00817700 6.01559800

C 30.48709300 105.78486500 6.74119600

O 30.84232200 105.48989300 7.87592800

C 27.98513000 105.39257200 6.54007900

C 27.77619000 104.43942200 7.72335500

C 28.41969600 103.13241000 7.25029000

H 29.47888300 105.14663700 4.93007500

H 27.93495400 106.45449600 6.81813200

H 27.23863000 105.21022000 5.75215000

H 28.31581100 104.81479400 8.60441000

H 26.71875300 104.30792700 7.99021600

H 28.81021000 102.53452000 8.08331400

H 27.70977300 102.51089500 6.68000100

N 31.02149200 106.80989700 6.04210900

C 31.95316800 107.71804700 6.65685500

C 31.19413700 108.72323100 7.55069900

O 29.97448700 108.81033200 7.51680500

C 32.77430800 108.45582200 5.57520200

O 31.89540200 109.33032100 4.84785600

C 33.46481400 107.51491900 4.60307600

H 30.57953600 107.10276400 5.17747200

H 32.64495000 107.12691400 7.27596400

H 33.53808100 109.06448700 6.08370500

H 34.11045900 108.09401900 3.92712400

H 32.72688600 106.96795400 3.99881100

H 34.07005900 106.77615500 5.14674600

N 31.97355300 109.55068500 8.29604500

C 31.52625700 110.88328700 8.66532400

C 32.59729700 111.86700600 8.20562800

O 33.78406100 111.62305700 8.42752500

C 31.29735600 111.11205400 10.17424700

O 30.71238900 112.36661600 10.41069700

H 32.98379200 109.43182900 8.28764800

H 30.57051700 111.07326600 8.16967000

H 30.60035500 110.33887200 10.53391900

H 32.25876100 110.97596800 10.70733300

H 31.31816600 113.09243600 10.14722000

N 32.15953200 112.99392400 7.61159600

C 33.05529900 114.11780000 7.49719100

C 33.10777000 114.85344300 8.85242700

O 32.28328600 114.63239600 9.74371900

C 32.72477600 115.01441600 6.29281100

C 31.29482600 115.42592300 6.15938600

N 30.57827900 115.14675400 5.01537500

C 30.40654000 116.10076600 6.97713000

C 29.32165700 115.64178900 5.18269900

N 29.18404100 116.23140800 6.35579600

H 31.15376400 113.12888400 7.46051400

H 34.05988600 113.71646700 7.31908700

H 33.39503200 115.88595200 6.32951300

H 33.01607300 114.45492700 5.39728400

H 30.93248200 114.59360300 4.22760900

H 30.59093900 116.48656000 7.97931500

H 28.55701500 115.55065100 4.41361100

N 34.11426300 115.73075600 8.97232900

H 34.87960100 115.74539100 8.29097400

H 34.22985900 116.21792800 9.85472100

O 33.17083300 111.21860600 4.33194300

C 32.09176200 110.70330100 5.06616000

C 30.79238900 111.44828800 4.75139000

N 29.66149700 110.92512600 5.47333500

C 30.53429700 111.49179700 3.24294400

O 29.37924600 112.27733800 3.06338900

C 31.74833500 112.12311900 2.54567300

O 31.78657400 113.48634800 2.93180700

C 32.98152500 111.27191300 2.92439200

C 34.34054900 111.60888300 2.27273600

C 28.97063100 111.64621000 6.39289800

O 29.34543400 112.75582200 6.78048400

C 27.68601600 111.02562000 6.88385100

O 35.24398000 112.39120500 3.03014400

H 32.36981400 110.87927000 6.10824700

H 30.92751400 112.47920000 5.09377400

H 29.40491700 109.95588900 5.32267000

H 30.37245300 110.47115100 2.85827600

H 31.62215200 112.04887700 1.45142200

H 32.23590600 114.03221700 2.23744100

H 32.73185800 110.26094500 2.55265800

H 34.87815000 110.66501600 2.11109300

H 34.15731000 112.04963100 1.27801000

H 26.85776900 111.42754700 6.27552800

H 27.52004900 111.32041700 7.92820200

H 27.69439900 109.93167500 6.79971200

C 34.86003600 113.68288500 3.42534700

H 33.77425000 113.78640000 3.40250100

C 35.52150200 114.75156500 2.52217700

H 35.09958400 115.72526500 2.82791900

C 37.02945300 114.74821100 2.76931900

H 37.42787900 113.75890700 2.49199500

C 37.30928000 114.97420100 4.25107400

H 36.88686000 115.96261200 4.52860800

C 36.59581800 113.90867300 5.08257200

H 37.04359400 112.92590200 4.84897900

C 36.72577600 114.13750000 6.58236900

H 37.78494500 114.03026200 6.86427700

H 36.15050000 113.36110300 7.10864300

O 36.20672600 115.39488900 6.99603000

H 36.87034000 116.08296400 6.85065700

O 35.20890000 113.90431000 4.76356800

O 38.68918200 114.91379400 4.52247000

H 39.12969100 115.43471000 3.83293400

O 37.73178200 115.67453800 1.96111200

H 37.36836800 116.56106400 2.11475800

N 35.27555100 114.55676500 1.10143600

H 36.09764100 114.48679600 0.51016700

C 34.08362400 114.73161100 0.50247500

C 34.05716000 114.65591400 -1.00267800

H 35.04357500 114.47378300 -1.44791900

H 33.36751900 113.85124700 -1.29855100

H 33.64784400 115.59982700 -1.39260100

O 33.04277500 114.93351300 1.14915400

C 28.61105600 112.08484700 1.89974000

H 29.25309900 111.93445200 1.01672400

C 27.79715100 113.37731900 1.77424000

H 27.23008800 113.37514200 0.83341500

C 26.81544400 113.44874200 2.95149900

H 27.40015800 113.54350100 3.88406900

C 25.99317100 112.17021500 3.05014700

H 25.35139800 112.09999100 2.15223400

C 26.89723200 110.93724000 3.08951300

H 27.47588400 110.96556200 4.02452900

C 26.13352100 109.63027400 3.05900600

H 25.54162800 109.57175600 3.98998200

H 26.87097500 108.80463300 3.07246600

O 25.31430500 109.58149800 1.90864300

H 24.74862000 108.80180400 1.97624900

O 27.80276300 110.94877500 1.98022400

O 25.21127500 112.17534400 4.23018400

H 24.79069500 113.04773800 4.27468300

O 25.89311000 114.51604000 2.83362200

H 26.37773600 115.35185400 2.87744400

N 28.71339000 114.50086500 1.74736500

H 29.56458200 114.37601600 2.28980900

C 28.67047800 115.49795100 0.81408100

C 29.84830700 116.45061100 0.87219900

H 30.78887200 115.91884300 0.65500900

H 29.70024100 117.25420400 0.14087800

H 29.94497500 116.88365700 1.88021700

O 27.75204800 115.60986700 0.01123700

L10

C 40.51336700 102.33791100 11.71592300

O 40.36960900 102.52034100 12.92258900

C 41.66003000 102.94890900 10.93687500

H 41.72414200 102.58870800 9.90113100

H 42.60197900 102.73093800 11.46075300

H 41.53482900 104.04291900 10.92743400

N 39.64606800 101.58737500 10.99199700

C 38.48569800 100.96289400 11.58802800

C 38.07070000 99.79877100 10.68573100

O 38.30961500 99.83014600 9.47212800

C 37.30491400 101.95301500 11.77059500

C 36.95126300 102.68183900 10.47768900

C 35.67084400 103.50549600 10.55838300

N 35.35384400 104.02190600 9.22960700

C 34.31213100 104.80906200 8.92827900

N 33.50473300 105.26053100 9.89030500

N 34.09504100 105.14656000 7.65328300

H 38.77233100 100.59738100 12.58331900

H 36.43023600 101.39681100 12.14465300

H 37.59164200 102.66938600 12.55515000

H 37.78398000 103.33423700 10.17282700

H 36.83409000 101.94430400 9.67315300

H 34.83907300 102.87775000 10.92649500

H 35.79494400 104.34254100 11.26574300

H 35.85175300 103.60745400 8.44626800

H 33.68349300 105.02467400 10.85763200

H 32.78101600 105.96755500 9.70405700

H 34.66889000 104.73070700 6.92672800

H 33.24284100 105.63502200 7.34978900

H 39.77674000 101.43822500 9.99629200

N 37.39320500 98.78293000 11.25591100

C 36.92914600 97.64278200 10.44097900

C 35.59167100 98.03515200 9.79651000

O 34.51859200 97.82542500 10.37575400

C 36.79490100 96.51179900 11.46347400

C 36.42265000 97.23553300 12.76461900

C 37.21793000 98.54428700 12.70176900

H 37.66994000 97.43423700 9.65735200

H 36.04982100 95.76634300 11.15792000

H 37.76843200 96.00861000 11.56291700

H 35.34576600 97.44939900 12.77170800

H 36.66869400 96.65554300 13.66446500

H 36.68896400 99.37816700 13.18320000

H 38.20516000 98.44037500 13.17998200

N 35.68164400 98.67177900 8.60708700

C 34.51935900 99.34934300 8.06587700

C 34.07238000 98.88976100 6.67509900

O 34.78701800 98.91585700 5.68700400

C 34.62726300 100.89341500 8.11000600

C 35.48350100 101.58186600 7.07151000

C 36.88705800 101.53022300 7.08180900

C 34.86742300 102.34658900 6.06488300

C 37.64587700 102.22486800 6.13605400

C 35.61253200 103.04921700 5.11853800

C 37.01511700 102.99369400 5.14784000

O 37.69831700 103.69424600 4.21190300

H 36.61155000 98.89486500 8.26091200

H 33.72727100 99.08467800 8.76413500

H 33.60411400 101.27492000 8.01149600

H 34.96747400 101.13827300 9.12703300

H 37.41679300 100.95716600 7.84454000

H 33.77815700 102.38321700 6.03276000

H 38.73767400 102.17423600 6.16977500

H 35.12176100 103.64165200 4.34314800

H 38.65063900 103.57622700 4.34079000

N 32.75289900 98.53449700 6.65876000

C 31.87979200 98.76320100 5.52959600

C 31.71225400 100.29306200 5.36063500

O 31.99689500 101.03988800 6.29904600

C 30.54877400 98.05708000 5.80152800

O 30.05407800 98.48011400 7.07501800

H 32.27949800 98.64334500 7.54566900

H 32.32607600 98.33770400 4.61957000

H 29.82067800 98.29857700 5.01367400

H 30.71990000 96.96789500 5.79868600

H 29.14116800 98.17133300 7.16689100

N 31.26688600 100.78436300 4.19130100

C 31.16067900 102.24301400 4.02492700

C 30.23383000 102.83588400 5.09626800

O 29.10299400 102.39914200 5.27909600

C 30.59990900 102.40614300 2.59556300

C 29.92594400 101.06072700 2.30125500

C 30.85450400 100.05734100 2.98406600

H 32.16006000 102.69370800 4.11756800

H 29.91468300 103.26106800 2.51690700

H 31.43377500 102.57878300 1.89882200

H 28.93688300 101.02533600 2.78137400

H 29.80663400 100.86097500 1.22756600

H 30.35305500 99.11441300 3.23082600

H 31.73282600 99.82660000 2.35608500

N 30.76313200 103.87365900 5.78219800

C 30.11624500 104.51919300 6.90524800

C 30.57630000 105.97455500 6.94150500

O 31.72525000 106.26870100 6.57480400

C 30.52853500 103.88957700 8.25778000

C 30.10741400 102.43289900 8.41835600

C 30.84635400 101.76058400 9.57183600

N 30.73363400 100.30709500 9.45470500

C 31.58672300 99.44265900 10.00855100

N 32.48911800 99.83113000 10.91358200

N 31.54896300 98.15248100 9.63966100

H 31.72017300 104.15781600 5.60200300

H 29.03182700 104.43168500 6.75982100

H 31.62510600 103.95604800 8.31807300

H 30.14130900 104.50941100 9.08180200

H 29.01758800 102.35460700 8.55992500

H 30.36598300 101.88768700 7.50382900

H 31.91262300 102.04084700 9.52244300

H 30.46166800 102.09732300 10.54886800

H 30.18389100 99.94502300 8.67629900

H 32.57031000 100.80124400 11.18903600

H 33.26220900 99.19781900 11.12574900

H 30.91350700 97.85966100 8.89904600

H 32.27144200 97.51817700 9.96856900

N 29.74724400 106.88872400 7.46176700

C 30.23329900 108.24065200 7.71057300

C 31.34135700 108.21852200 8.77461400

O 31.54128700 107.24018100 9.49542500

C 28.97555300 108.99091600 8.19497900

C 28.16143200 107.89057600 8.88374900

C 28.38843600 106.66922700 7.98917400

H 30.65186100 108.67544100 6.79145500

H 29.22262000 109.83485600 8.85306800

H 28.43616300 109.38796200 7.32204900

H 28.56424400 107.69437200 9.88912700

H 27.09649200 108.13919600 8.98435800

H 28.32418600 105.72669600 8.54730700

H 27.66409600 106.63210300 7.15911700

N 32.03734700 109.36603400 8.86224100

C 32.99498200 109.65437600 9.90312800

C 32.67179100 111.04563600 10.46561200

O 31.96882800 111.82778900 9.82573800

C 34.45466300 109.66356000 9.38063100

O 34.58742800 110.81510400 8.54024800

C 34.82095500 108.39204900 8.63843500

H 31.76106000 110.16403900 8.29613400

H 32.90424500 108.87956000 10.67754800

H 35.12144700 109.78444300 10.25095200

H 35.85455800 108.44541700 8.27242600

H 34.15401800 108.23932500 7.77817200

H 34.72988300 107.52944000 9.31127300

N 33.27796200 111.36746800 11.62579000

C 33.37515000 112.72677400 12.11340300

C 34.81170400 113.21880800 11.94709500

O 35.75457500 112.42589900 11.97751000

C 32.99213600 112.81052800 13.61510500

O 33.18243600 114.10112100 14.14064200

H 33.91671500 110.69814100 12.04548800

H 32.68030500 113.34584100 11.53368700

H 31.92637400 112.55141700 13.70385800

H 33.57027900 112.04811700 14.16807000

H 34.11332600 114.20459000 14.40730800

N 34.97166200 114.55601600 11.86708400

C 36.27549500 115.18531400 12.02510500

C 36.56850100 115.44078600 13.52136900

O 36.95681700 116.51470700 13.94282000

C 36.40526100 116.45127500 11.17149200

C 35.26298900 117.41358300 11.30046600

N 34.39586100 117.65412300 10.25427000

C 34.80082500 118.21410900 12.33194600

C 33.47326700 118.55939200 10.67993300

N 33.68955400 118.92175600 11.93201100

H 34.15999600 115.14411100 12.04079100

H 37.01737600 114.44649400 11.68514500

H 37.35040600 116.93906600 11.44830600

H 36.48616700 116.14059900 10.12082300

H 34.42775300 117.16094500 9.35144200

H 35.22747200 118.30500200 13.32867000

H 32.66875600 118.91529600 10.03631300

N 36.32020100 114.36198100 14.33272700

H 36.38333200 113.43048700 13.92372200

H 36.64697300 114.44246200 15.29233300

O 36.89427700 111.05340300 8.28109900

C 35.71803700 111.62119400 8.77377100

C 35.45287800 112.99906700 8.14463600

N 34.14324100 113.46884900 8.57451600

C 35.60102800 113.00607900 6.61423000

O 35.71124700 114.32982000 6.11999900

C 36.87396600 112.26380600 6.17697800

O 38.00484900 113.03708400 6.51547100

C 36.94609300 110.90513700 6.85614800

C 38.22074200 110.12316300 6.58196200

O 38.24319200 108.91521100 7.31785300

C 33.79743500 114.76336500 8.62437900

O 34.58151900 115.66540800 8.26361700

C 32.42084200 115.08101600 9.14680500

H 35.88196900 111.73267600 9.85566400

H 36.21779200 113.67829000 8.54946900

H 33.42596100 112.76632800 8.75774000

H 34.72267700 112.49572400 6.17739800

H 36.81612200 112.10191600 5.08152700

H 37.73869700 113.95130800 6.31414700

H 36.07786800 110.30833800 6.52763300

H 38.27019400 109.86818900 5.51256900

H 39.08812000 110.76400600 6.82242300

H 38.09094800 109.16606700 8.24196100

H 32.52081600 115.67591300 10.06689600

H 31.83377500 114.17933500 9.35591300

H 31.90092200 115.71275200 8.41215800

H 35.38508300 114.95503500 6.80102500

L11

C 44.06732100 94.65746300 7.28953800

O 43.60011700 93.93167200 8.16485300

C 45.53106900 95.04894300 7.26290000

H 45.81798500 95.60218300 6.35835900

H 46.14334900 94.13917200 7.34613200

H 45.74573300 95.67067300 8.14601200

N 43.30495600 95.17008400 6.29320200

C 41.88872900 94.89936900 6.18279700

C 41.48078500 95.18366100 4.73647800

O 42.15590900 95.94840200 4.04114000

C 41.05076000 95.76633500 7.15319900

C 41.14718200 97.26683600 6.88565300

C 40.23082300 98.05213700 7.81705700

N 40.23475400 99.47090300 7.46359100

C 39.25459700 100.33184400 7.73260300

N 38.21688300 99.97381800 8.48628600

N 39.27960400 101.56276300 7.18354500

H 41.71649800 93.84332500 6.43819500

H 39.99618400 95.45667100 7.09514700

H 41.39320200 95.52878700 8.17195400

H 42.18847500 97.60573600 7.01319700

H 40.85512500 97.48143100 5.84733100

H 39.20092800 97.67565600 7.71322400

H 40.53312200 97.91903500 8.86998400

H 41.06011900 99.83339200 6.99537900

H 38.19259100 99.08248000 8.96447200

H 37.41893800 100.58771000 8.59944800

H 40.10710900 101.86774500 6.68243100

H 38.71534700 102.30183400 7.59037600

H 43.70152600 95.75374200 5.56255500

N 40.32588700 94.65462000 4.28437400

C 39.80076700 95.11297900 3.00077900

C 39.56813400 96.62622000 3.05761300

O 39.19877500 97.17824700 4.10191900

C 38.48397700 94.32239200 2.83394000

C 38.08052700 93.97941300 4.27258700

C 39.42203900 93.70703500 4.95320900

H 40.51307900 94.89665900 2.19126400

H 37.72322700 94.89562900 2.28597300

H 38.68933300 93.40640000 2.26037800

H 37.59278900 94.84198200 4.75150300

H 37.39778500 93.12112400 4.33364900

H 39.38609600 93.87277100 6.03549400

H 39.76159800 92.67235500 4.77562700

N 39.73626300 97.27145800 1.89129800

C 39.84260200 98.71519000 1.75783500

C 38.87411900 99.27601400 0.70181800

O 39.22061600 100.09789700 -0.13527900

C 41.30679200 99.13959900 1.52262500

C 42.02181800 98.31952500 0.47334800

C 41.99195000 98.67017900 -0.88473200

C 42.70161600 97.14255300 0.83651000

C 42.62432900 97.88400100 -1.84962700

C 43.33183700 96.34569000 -0.11962100

C 43.29864400 96.71330900 -1.47267900

O 43.92861300 95.90894700 -2.36631000

H 40.10524100 96.73184800 1.11312200

H 39.50345500 99.14602800 2.71113800

H 41.30433300 100.20422700 1.25466700

H 41.82909500 99.03511700 2.48486800

H 41.45110900 99.56760100 -1.18877100

H 42.72739300 96.83661000 1.88588700

H 42.59443600 98.17689800 -2.90308200

H 43.85777000 95.43268900 0.16747600

H 43.83058100 96.27051100 -3.25893500

N 37.58769900 98.84769900 0.81413000

C 36.54087600 99.63245700 0.19047000

C 36.57070800 101.06403000 0.77013000

O 37.07317200 101.27683700 1.87195700

C 35.17419900 98.97123800 0.36218800

O 34.78215800 98.96625300 1.73425200

H 37.33907700 98.35906600 1.66893500

H 36.74250600 99.68266800 -0.88935100

H 34.43268900 99.52205800 -0.23502800

H 35.23312400 97.94118700 -0.02928500

H 33.85455200 98.69308600 1.78176200

N 36.01754000 102.05191600 0.04018300

C 35.93991800 103.39227400 0.61658000

C 34.93398500 103.40602000 1.77595000

O 33.93448500 102.69283600 1.77167700

C 35.46199700 104.26380700 -0.56272600

C 34.65298300 103.28526700 -1.42094100

C 35.45130700 101.98383700 -1.31445600

H 36.92607700 103.70298100 0.99020900

H 34.88486000 105.13627700 -0.22642900

H 36.33906300 104.63272800 -1.11614300

H 33.65490700 103.13958200 -0.98231300

H 34.53258000 103.61696200 -2.46131700

H 34.81746600 101.09876800 -1.44979000

H 36.26115000 101.94329500 -2.06338200

N 35.22534400 104.28565600 2.75783200

C 34.32039900 104.60803500 3.84406800

C 34.45269700 106.10838400 4.11334700

O 35.53133700 106.67377400 3.92228200

C 34.62943500 103.84411100 5.15117300

C 34.57547100 102.31625700 5.00154100

C 35.94312300 101.70916600 4.68887900

N 35.79739000 100.31797700 4.27907700

C 36.75855600 99.39704500 4.34980800

N 37.91497500 99.62036400 4.98632400

N 36.55734000 98.18839500 3.80553700

H 36.05089200 104.87654700 2.69699000

H 33.30676800 104.34641100 3.51111600

H 35.61900000 104.16627400 5.51672200

H 33.90865700 104.17517100 5.91269300

H 34.18664900 101.85736100 5.92360800

H 33.87390800 102.05650900 4.19352800

H 36.45244700 102.26952000 3.89197000

H 36.56857200 101.75320700 5.59273600

H 35.09983900 100.13813700 3.55700900

H 38.18027900 100.55490200 5.27169700

H 38.63985900 98.90725600 4.89765200

H 35.73625700 98.02964700 3.22586900

H 37.26488500 97.47186900 3.94703500

N 33.38045900 106.74949500 4.61654500

C 33.52219400 108.12334400 5.07943800

C 34.37812300 108.16765100 6.35142200

O 34.51387600 107.19918700 7.08868800

C 32.07295400 108.55232800 5.38836300

C 31.41241400 107.23685400 5.81648000

C 32.04486100 106.19555700 4.88753500

H 33.99270000 108.74780600 4.30645300

H 32.03598800 109.32733000 6.16541000

H 31.60750800 108.94668400 4.47206600

H 31.67679100 107.01102400 6.86026400

H 30.31694700 107.25474700 5.73526700

H 32.10492800 105.20531500 5.35792300

H 31.47761100 106.09414900 3.94674900

N 34.92076700 109.38093300 6.61540400

C 35.39485300 109.70288100 7.93748000

C 34.20288000 109.83168500 8.90687600

O 33.04388800 109.92392100 8.52362000

C 36.20739200 111.00877200 7.88598400

O 35.34080700 111.96494100 7.29020900

C 37.50912900 110.85298000 7.11079000

H 34.63431700 110.18222600 6.06026000

H 36.04193500 108.88910900 8.29597100

H 36.43671300 111.30265600 8.92670400

H 38.04252100 111.81565100 7.06631700

H 37.31193300 110.51248200 6.08363200

H 38.16977000 110.12335300 7.60311700

H 35.82907400 112.78966900 7.16356700

N 34.53260900 109.85043500 10.22057400

C 33.53939500 110.14143500 11.22401800

C 32.92848700 111.52875500 11.01557500

O 33.60849400 112.48901200 10.65457300

C 34.19933200 110.13618200 12.62620600

O 33.30907100 110.49182900 13.65364100

H 35.50734900 109.79629900 10.49514500

H 32.74181600 109.38163000 11.19802700

H 34.56491200 109.11758600 12.82955100

H 35.07516100 110.81073400 12.59049600

H 33.21621700 111.46224200 13.67778000

N 31.65087200 111.63249500 11.43054000

C 30.97632100 112.89475200 11.69338300

C 31.44628300 113.55723900 13.01593000

O 30.66583700 114.07703500 13.79111300

C 29.46033600 112.71462500 11.66753400

C 28.94877900 111.66426400 12.60881300

N 27.62153800 111.29376900 12.62596000

C 29.53856000 110.87834800 13.58556500

C 27.46205400 110.33404500 13.58242800

N 28.60274300 110.05805900 14.17906200

H 31.18669300 110.79241400 11.76127600

H 31.26591800 113.59451200 10.89225000

H 29.00367200 113.68569100 11.91150700

H 29.15835400 112.46646100 10.63696800

H 26.89154200 111.67339700 12.03207700

H 30.58550400 110.85172100 13.88550700

H 26.49413600 109.87910200 13.79175500

N 32.79898800 113.50089200 13.23557100

H 33.40582400 113.43256100 12.41520900

H 33.14236000 114.07212900 14.00377600
